# Hit-to-Lead Optimization of Energy-Coupling Factor (ECF) Transporter Inhibitors as Novel Antibiotic

**DOI:** 10.1021/acs.jmedchem.5c02721

**Published:** 2026-06-06

**Authors:** Ioulia Exapicheidou, Aleksei Tsarenko, Lena Zeller, Hamza Ibrahim, Carole Baumann, Atanaz Shams, Patrick A. Hoffmann, Yue Li, Andreas M. Kany, Jennifer Hermann, Dirk J. Slotboom, Rolf Müller, Andrea Volkamer, Mostafa M. Hamed, Eleonora Diamanti, Anna K. H. Hirsch

**Affiliations:** † 9296 Helmholtz Institute for Pharmaceutical Research (HIPS) - Helmholtz Centre for Infection Research (HZI), Campus Building E 8.1, Saarbrücken, Saarland D-66123, Germany; ‡ Saarland University, Department of Pharmacy, Campus E8.1, Saarbrücken, Saarland 66123, Germany; § Saarland University, PharmaScienceHub (PSH), Campus E2.1, Saarbrücken 66123, Germany; ∥ Groningen Biomolecular Sciences and Biotechnology Institute, 3647 University of Groningen, Nijenborgh 4, Groningen 9747AG, The Netherlands; ⊥ Data Driven Drug Design, Center for Bioinformatics, Saarland Informatics Campus, Saarland University, Saarbrücken 66123, Germany

## Abstract

Multiparameter optimization of a previously identified class of inhibitors of the energy-coupling factor (ECF) transporters enabled the confirmation of *in vivo* efficacy. ECFs are a class of transmembrane proteins that play a vital role in the active translocation of essential nutrients across cell membranes and are therefore important in the fight against antimicrobial resistance. Aiming to improve the drug-like properties of our inhibitory class, we performed a focused structure–activity relationship study around the Eastern part of our starting molecule **3** by exploiting click chemistry. Our multiparameter optimization resulted in compounds with enhanced metabolic stability and solubility, potent activity against both a panel of Gram-positive bacteria, and against the ECF transporters. We further demonstrate rapid bacterial killing using *Enterococcus faecium* as a model organism and confirmed *in vivo* efficacy of the best compounds in *Galleria mellonella* larvae and *Danio rerio* (zebrafish) infection models, highlighting the therapeutic potential of our approach.

## Graphical abstract

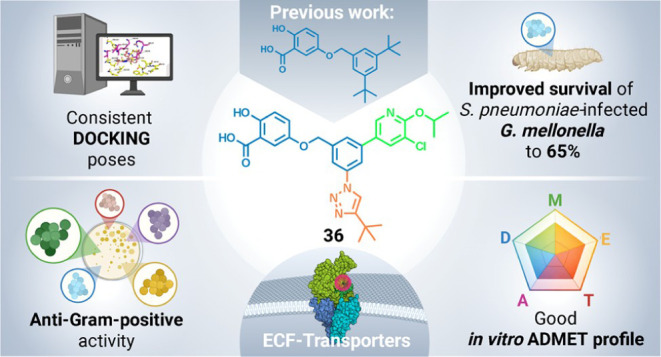

## Introduction

The emergence of antimicrobial resistance (AMR) poses a significant and growing health threat to the effective treatment of common bacterial infections worldwide. Over the past decades, the development of novel antibacterial agents has received limited attention, mainly due to economic constraints. However, the continued emergence of new resistant strains underscores the need for a coordinated strategy to combat AMR, the exploration of new targets, and the development of new drug candidates with unprecedented modes of action.^1,2^

Herein, we address these challenges by targeting the energy-coupling factor (ECF) transporters, a relatively underexplored class of transmembrane proteins involved in the uptake of vitamins in a wide range of bacteria, predominantly in Gram-positive species (*e.g*., *Enterococcus faecalis*, *E. faecium*, and *Streptococcus pneumoniae*).^3^ Due to their critical role in regulating the homeostasis of vitamins in bacteria as well as their absence in human cells, ECF transporters are considered a promising antimicrobial target. They consist of a substrate-binding portion called S-component and a tricomponent energizing module (ECF-module), comprising the transmembrane protein EcfT and the two cytosolic nucleotide-binding domains EcfA and EcfA’. ECF transporters are classified in two groups. In group I, a single S-component interacts exclusively with a dedicated ECF-module, whereas in group II, different S-components compete for binding to the same ECF-module (Figure 1), allowing to block the uptake of several vitamins with a single inhibitor simultaneously.^4–6^

**Figure 1:**
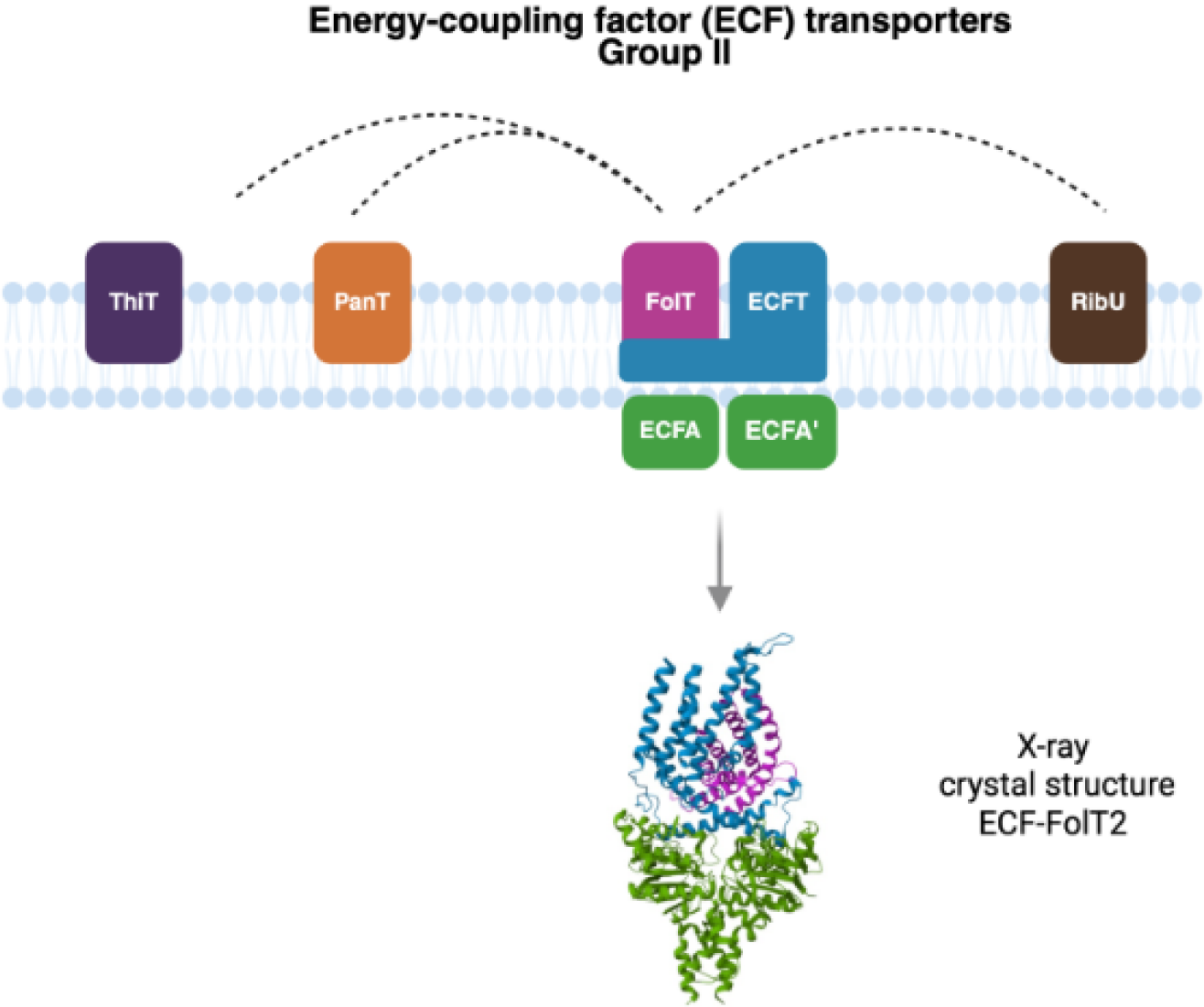
Schematic representation of Group II Energy-Coupling Factor (ECF) transporters. Multiple S-components (shown in different colors) interact with a shared ECF module composed of the membrane-embedded protein EcfT (shown in blue) and the two ATPase subunits EcfA and EcfÁ (shown in green). The X-ray crystal structure is of *Lactobacillus delbrueckii* ECF-FolT2 (PDB ID: 5JSZ).

With the aim to identify ECF transporter inhibitors, we recently performed structure-based virtual screening (SBVS) campaigns utilizing the X-ray crystal structure of the folate-specific transporter ECF-FolT2 from *Lactobacillus delbrueckii* (PDB ID: 5JSZ) and both the Princeton Express and our in-house libraries. These *in silico* screenings resulted in three chemically different hits.^7–9^

To further optimize the hits coming from the SBVS, we pursued a target-directed dynamic combinatorial chemistry (tdDCC) approach using ECF-PanT in *S. pneumoniae*.^10^ Although, we demonstrated for the first time the applicability of the challenging tdDCC to a transmembrane protein, this work did not increase the activity of our hits. It was therefore essential to carry out a comprehensive structure–activity relationship (SAR) study. Initially, we showed that the replacement of the phenolic OH of compound **1** with a Boc-protected amine enhanced activity due to an additional interaction furnished by the Boc moiety as in compound **2**, Figure 2.^9^ Then, as a continuation of our study around this chemical class, a SAR investigation of around 70 derivatives of **1**, led to the successful replacement of the naphthalenyl moiety of **1** with a bis-*tert*-butyl group (compounds **3** and **4**, Figure 2). In parallel to the synthetic chemistry work, we also gained valuable insights into the target validation in *S. pneumoniae* using CRISPR screening technology. Overall, these findings paved the way for further research into this class of inhibitors, this time with a primary focus on improving the drug-like properties.

**Figure 2:**
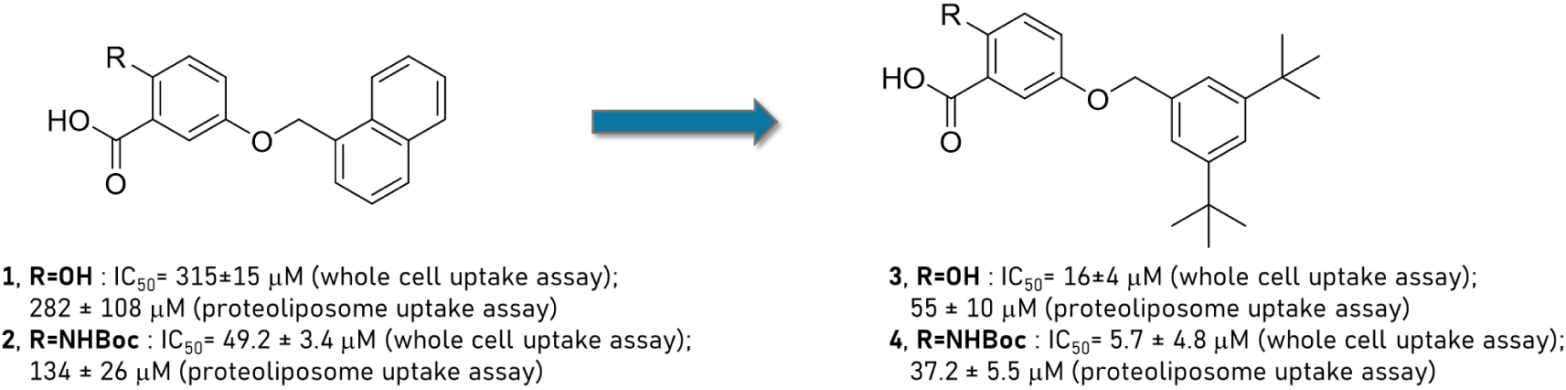
Illustration showing the chemical structure evolution from **1** and **2** to compounds **3** and **4**. IC_50_ values measured with the *Lactobacillus casei* whole-cell uptake assay and *Lactobacillus delbrueckii* proteoliposome uptake assay.

## Results and Discussion

Multiparameter optimization is a critical aspect of the drug-discovery pipeline. Accordingly, the optimization of absorption, distribution, metabolism, excretion, and toxicity (ADME-T) has become increasingly important alongside to the efficacy in the hit-to-lead and lead-optimization phases of drug discovery.^11^

Encouraged by the improved potency of compounds **3** and **4**, we envisaged that this chemical series might hold a potential for further optimization. Our goal was not only to further enhance the potency against ECF transporters, but also to provide the basis for a future lead compound with a balanced profile. We therefore decided to evaluate compounds **1**, **3,** and **4** for their physicochemical properties before proceeding with subsequent rounds of design, synthesis, and testing of new derivatives.

We initially compared the kinetic solubility (S) in 1% DMSO/PBS and logD_7.4_ of **1**, **3**, and **4**. Interestingly, S was >200 μM for both **1** and **3** and logD_7.4_ was 1.69 and −0.43, respectively, whereas **4** showed comparatively lower solubility (S = 67 ± 32 μM) and higher lipophilicity (logD_7.4_ = 4.33). This result is likely attributed to the presence of the NH-Boc group, which significantly affects the solubility of this chemical series. For this purpose, we selected compound **3** as a starting point in the present SAR study.

Building on **3**, we decided to focus on the design and synthesis of novel disubstituted salicylic acid derivatives, with the aim of exploring and optimizing the Eastern part of the molecule in a multiparameter fashion (Figure 3).

**Figure 3:**
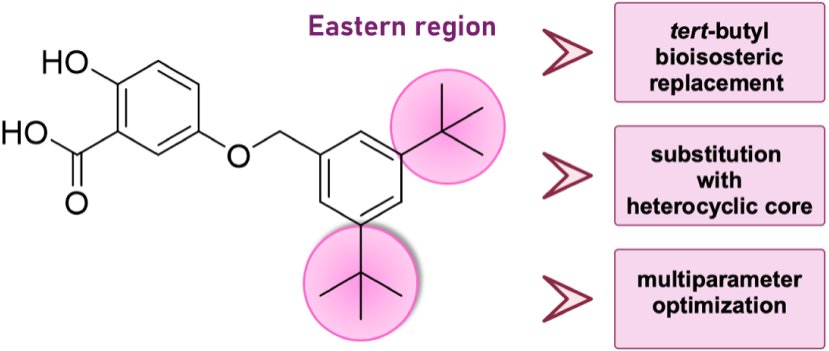
Schematic representation of the structure–activity relationship strategy and its objectives, using compound **3** as a starting point for the design of the new derivatives.

### SAR Study

Considering the metabolic liability of the *tert*-butyl moiety,^12,13^ we first synthesized seven aliphatic and halogenated derivatives. These compounds bear a chlorine (**5**) and/or a bromine (**6**, **7**) atom, as well as the classical *tert*-butyl bioisosteres such as trifluoromethyl (**8**), cyclopropyl (**9**), and 2,2,2-trifluoroethyl (**10**) substituents.^13^ The synthesis is depicted in Schemes 1 and 2. Etherification of 2,5-dihydroxybenzoate (**37**) with the alkyl bromide derivatives **38**–**41** using K_2_CO_3_ in acetone released the methyl ester intermediates (**42**–**45**) that upon hydrolysis in the presence of 10% aq. NaOH afforded compounds **5**–**8** in optimum yield (Scheme 1). We followed a classical nucleophilic substitution (S_N_2) between the alkoxide **37**, generated in the presence of K_2_CO_3_, and differently substituted alkyl halides (**38**–**41**) to afford the final ether products. The regioselectivity has been already discussed previously by us.^9^

**Scheme 1:**
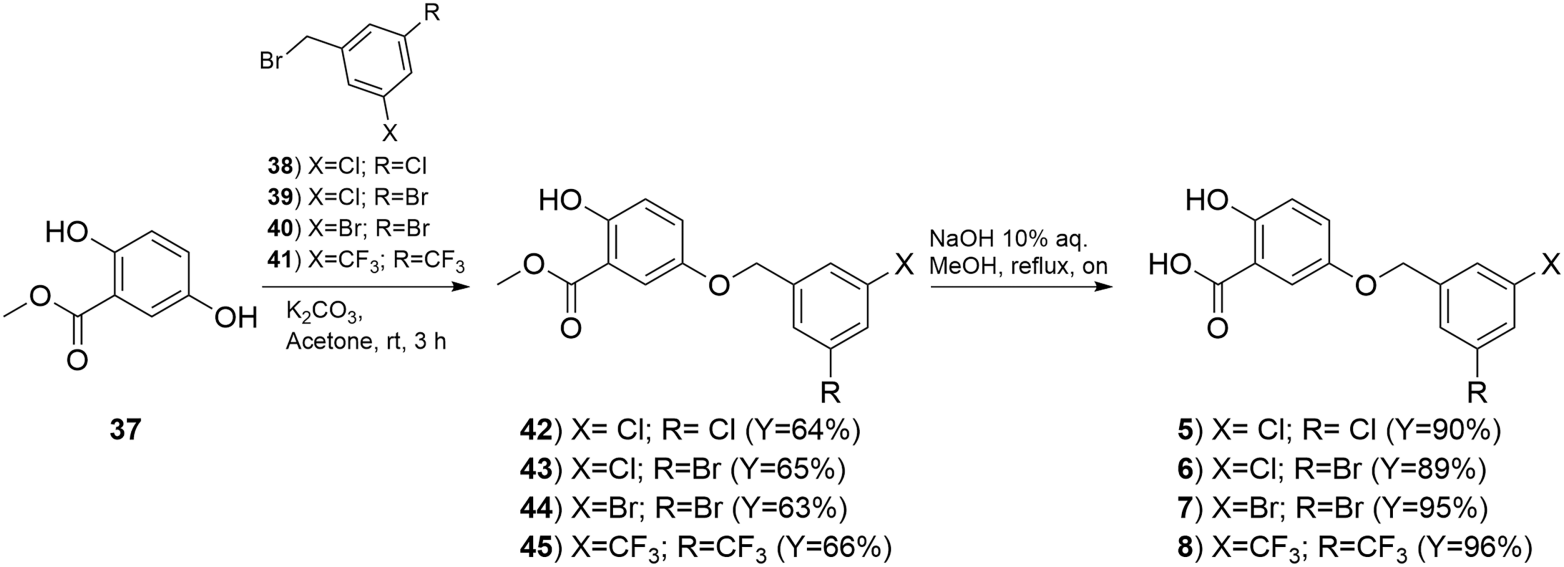
Synthetic Scheme of Disubstituted Derivatives **5–8**

All the surrogate derivatives were tested in our whole-cell uptake assay at a concentration of 50 μM using *L. casei* as a model organism.^7^ Unfortunately, this set of analogues (**5**–**10**) showed a decrease in potency compared to **3** (Table 1).

**Table 1:**
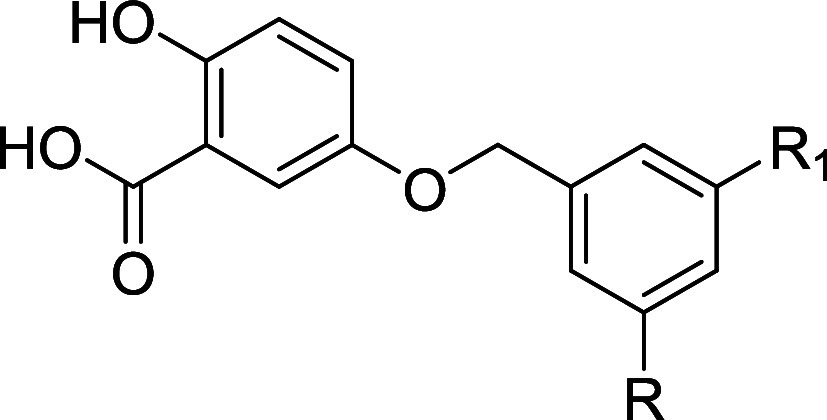
Inhibitory Potency of **3**, **5**–**10** in the Whole-Cell Uptake Assay with *Lactobacillus Casei* and Radioactively Labeled Substrate

Compound	R^1^	R	% Inh. at 50 μM ± S.D.^a^
**3**	*tert*-butyl	*tert*-butyl	73 ± 4
**5**	Cl	Cl	n.i.
**6**	Br	Cl	64 ± 5
**7**	Br	Br	42 ± 8
**8**	CF_3_	CF_3_	32 ± 29
**9**	cyclopropyl	cyclopropyl	68 ± 3
**10**	2,2,2-trifluoroethyl	2,2,2-trifluoroethyl	n.i.

a S.D.: standard deviation derived from two independent experiments; n.i. no inhibition.

In response to the previous results, where the bioisosteric replacement of the *tert*-butyl group was not tolerated, we decided to pursue an alternative strategy and turned to the use of heterocyclic derivatives. The idea of incorporating more drug-like moieties, which could potentially improve both metabolic stability and solubility while maintaining activity, led us to explore a subset of heterocyclic compounds. Heterocycles are widely utilized in medicinal chemistry due to their diverse biological activities, particularly as antibacterial and antiviral agents, and often favourable pharmacological properties.^14,15^ Having this in mind, we introduced various differently substituted five- and six-membered heterocyclic rings (**11–36**) through a multistep synthetic route (Schemes 2–4).

**Scheme 2:**
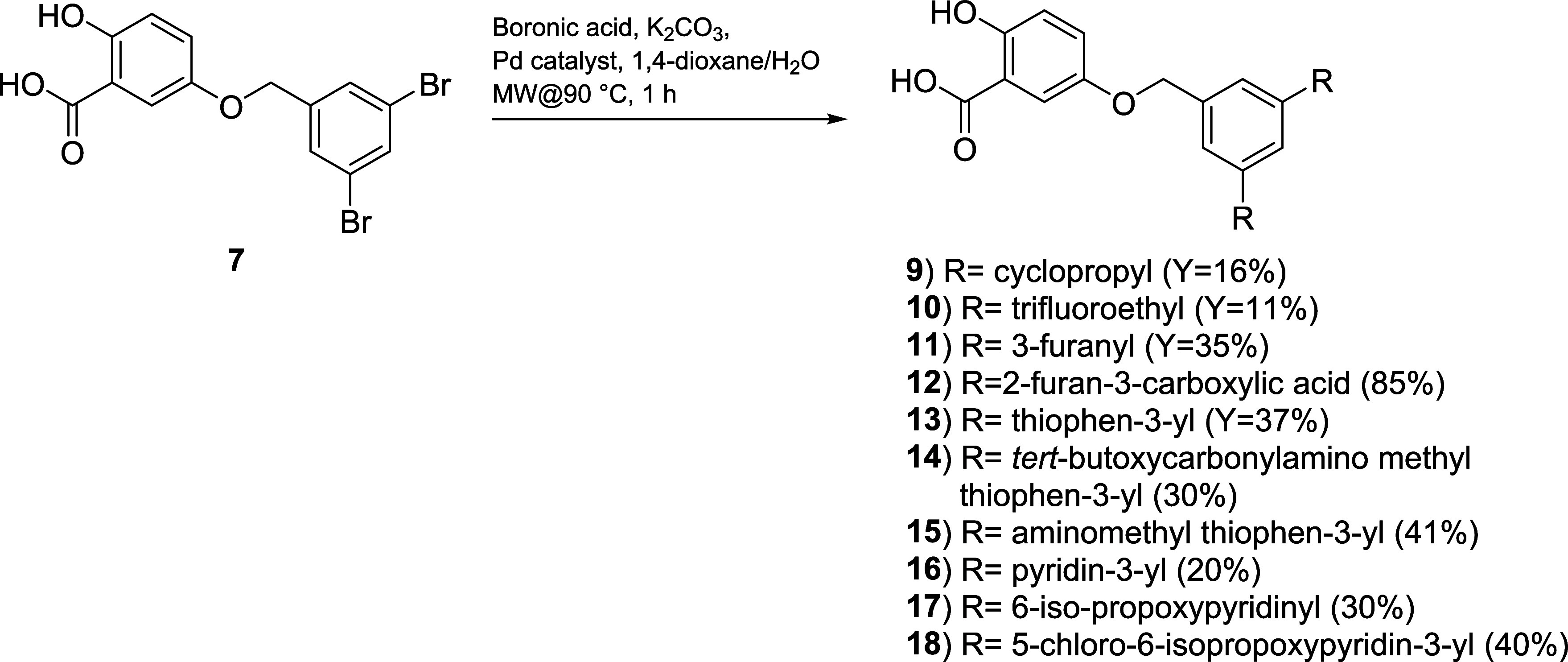
Synthetic Scheme of Compounds **9–18**

**Scheme 3:**
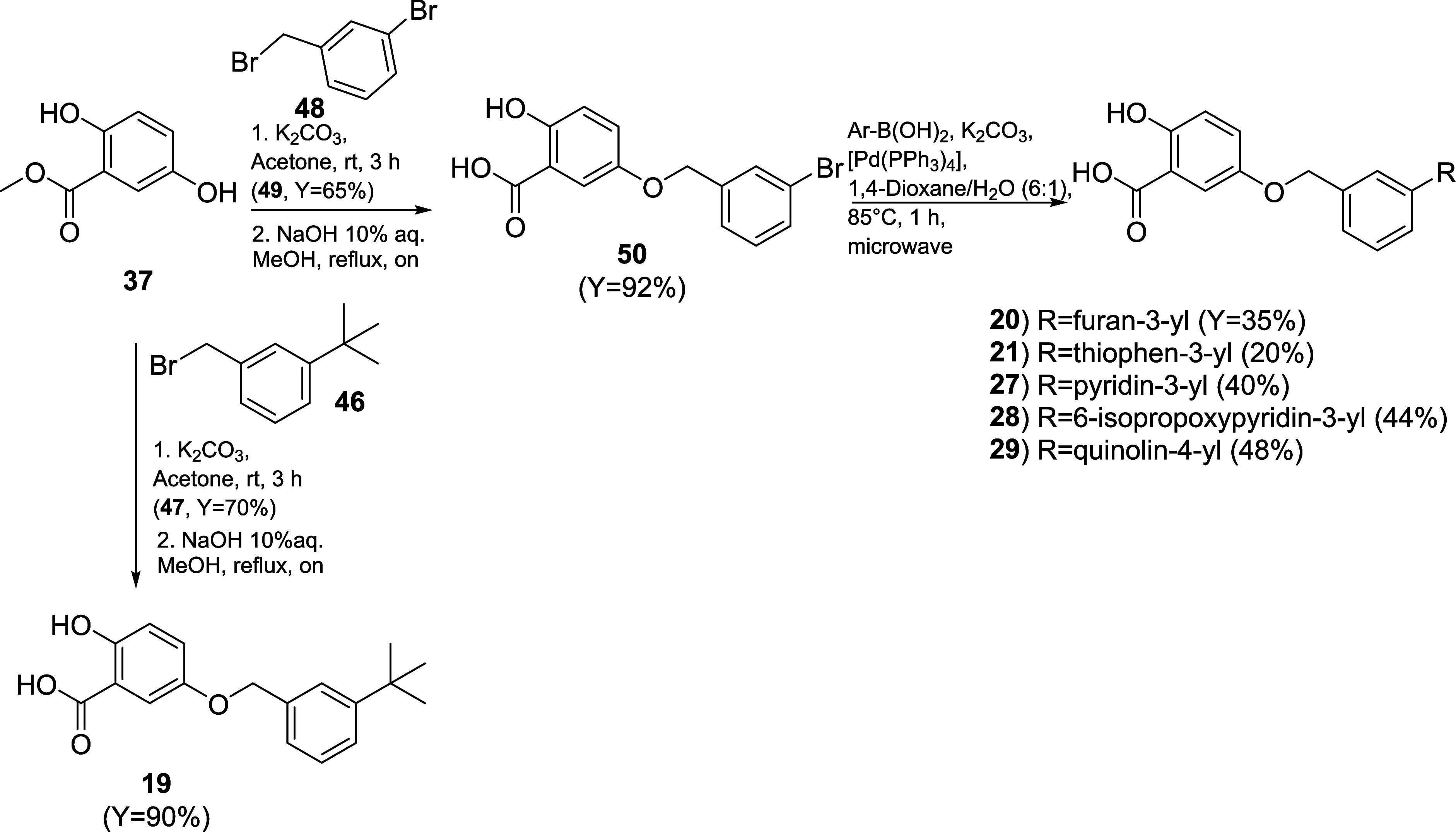
Synthetic Scheme for the Monosubstituted Derivatives **19–21** and **27–29**

**Scheme 4:**
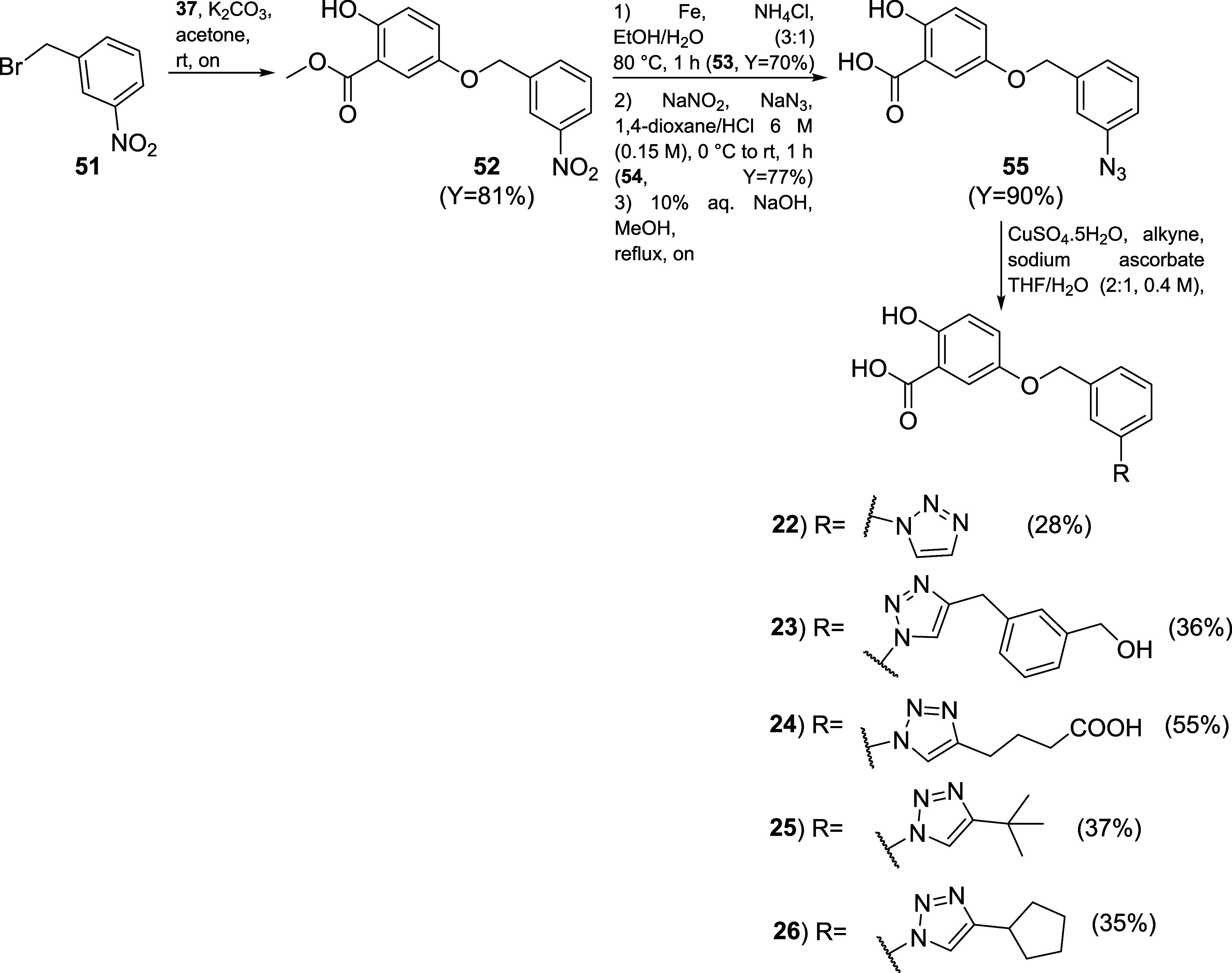
Synthetic Scheme for the Monosubstituted Derivatives **22–26**

Treatment of compound **7** with various aryl boronic acids afforded the desired final compounds **9**–**18** through a Suzuki cross-coupling reaction (Scheme 2).

In a similar manner, we prepared the monosubstituted derivatives **20**, **21,** and **27**–**29** (Scheme 3), while we employed a different synthetic strategy for the 1,4-triazole series (**22**–**26**) (Scheme 4). The triazole formation occurred between azides and terminal alkynes in the presence of a Cu­(II) source and sodium ascorbate as reducing agent. We followed standard conditions for this series of derivatives.

An eight-step pathway has been designed and optimized to get the disubstituted series (**30**–**36**, Scheme 5). By reacting **57** with **37**, we obtained the intermediate **58** that underwent a Suzuki cross-coupling reactions to introduce the 5-chloro-6-isopropoxypyridin-3-yl group (**59**). Then, nitro reduction in the presence of iron and ammonium chloride in ethanol/H_2_O yielded the corresponding amine **60**, that upon treatment with sodium nitrite and sodium azide, resulted in the azide **61**. Saponification of **61** with 10% aq. NaOH in methanol yielded the desired intermediate **62** that has been subjected to click reaction with differently substituted alkynes to afford the desired final target molecules (**30**–**36**).

**Scheme 5:**
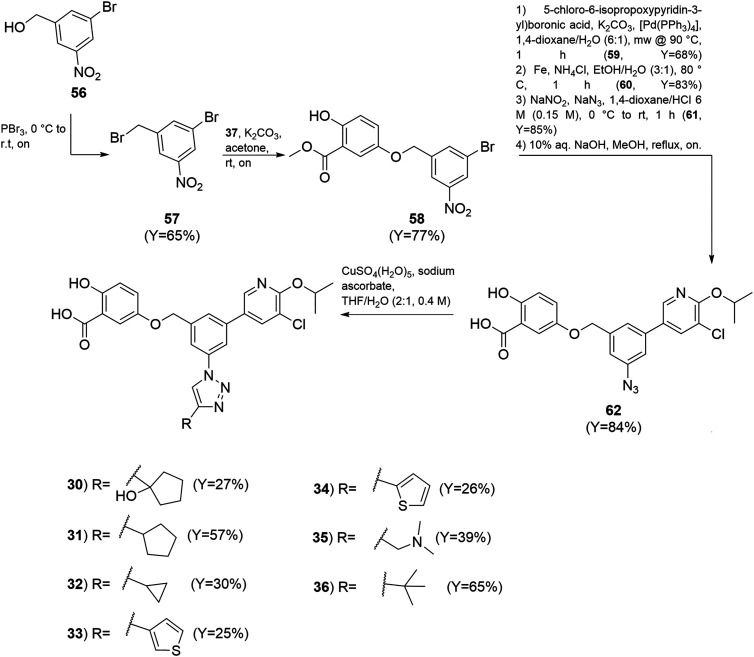
Synthetic Scheme of the Triazolyl Derivatives **30–36**

Compound **13** proved to be the most potent unsubstituted 5-membered ring (IC_50_ = 26 ± 2 μM, *L. casei* whole-cell uptake assay, Table 2) compared to the furan (**11**), which showed just 50% inhibition at 50 μM. Introduction of a Boc-amine in position 4 of the thiophenyl ring resulted in compound **14** showing a 3-fold boost in potency (IC_50_ = 8 ± 2 μM), whereas its deprotection (**15**) was found to be detrimental. To further enrich our library, we shifted toward pyridine (**16**) serving as a six-membered heterocycle, which unfortunately did not display any potency on target. However, there is a significant increase in potency when an *iso*propoxy group is added to **16** (**17**, IC_50_ = 9 ± 2 μM, Table 2). A further boost in the activity has been obtained by the introduction of a chlorine atom at position 5 of the same ring (**18**, IC_50_ of 4 ± 1.5 μM). Among all the disubstituted derivatives tested at two different concentrations (50 and 100 μM) in the whole cell-based assay, compounds **14**, **17,** and **18** emerged as the most potent analogues within this series.

**Table 2:**
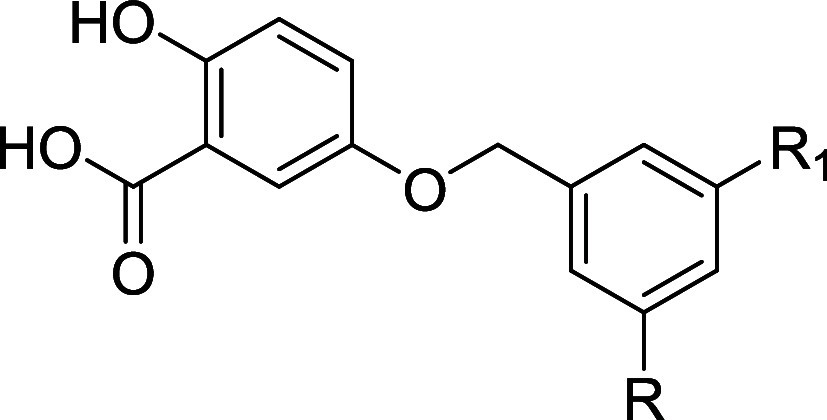

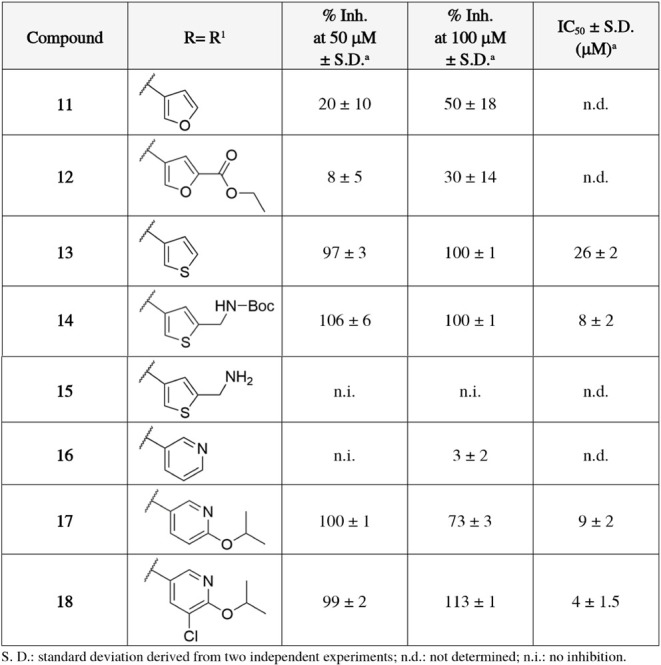
Inhibitory Potency and IC_50_ Determination of **11–18** in the Whole-Cell Uptake Assay with *Lactobacillus Casei* and Radioactively Labeled Substrate

To further validate the pivotal role of the disubstitution pattern, we synthesized a small set of monosubstituted derivatives (**19**–**29**, Table 3). This subset of compounds was specifically selected for direct comparison with their respective disubstituted counterparts (**19** *vs* **3**, **20** *vs* **11**, **21** *vs* **13**, **27** *vs* **16** and **28** *vs* **17**) alongside exploring new moieties with compounds **22**–**26** and **29**. We initially screened our derivatives at 200 and 100 μM and determined the IC_50_ values for compounds that showed >70% inhibition at 200 μM. Among the new moieties introduced, the triazolyl core caught our attention for several reasons. First, this modification may enhance the solubility of the parent molecule. Second, it contributes to the expansion of our compound library. Third, it enables the application of click chemistry, a highly efficient and straightforward synthetic strategy. Furthermore, the 1,2,3-triazolyl ring is widely utilized in anti-infective drug discovery.^16,17^ Hence, we synthesized the unsubstituted 1,4-triazole (**22**) and four differently substituted triazoles in position 4 (**23**–**26**) and checked their inhibition of ECF-FolT2. Notably, the introduction of polar groups like in **23** and **24** was not tolerated, while aliphatic chains (**25**) or cycloalkyl systems (**26**) slightly enhanced the activity. In summary, this series of monosubstituted compounds displays a reduction in potency.

**Table 3:**
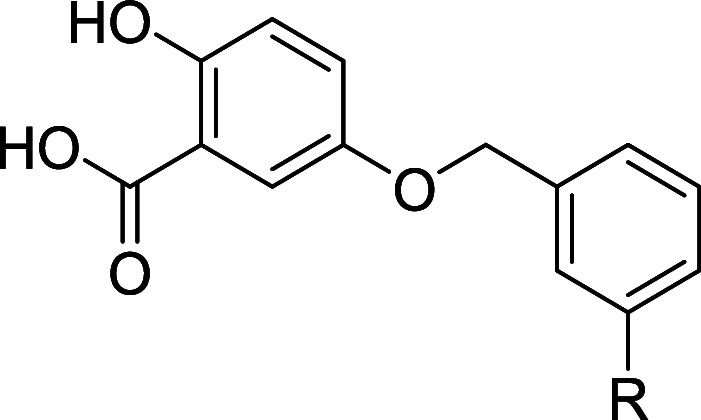

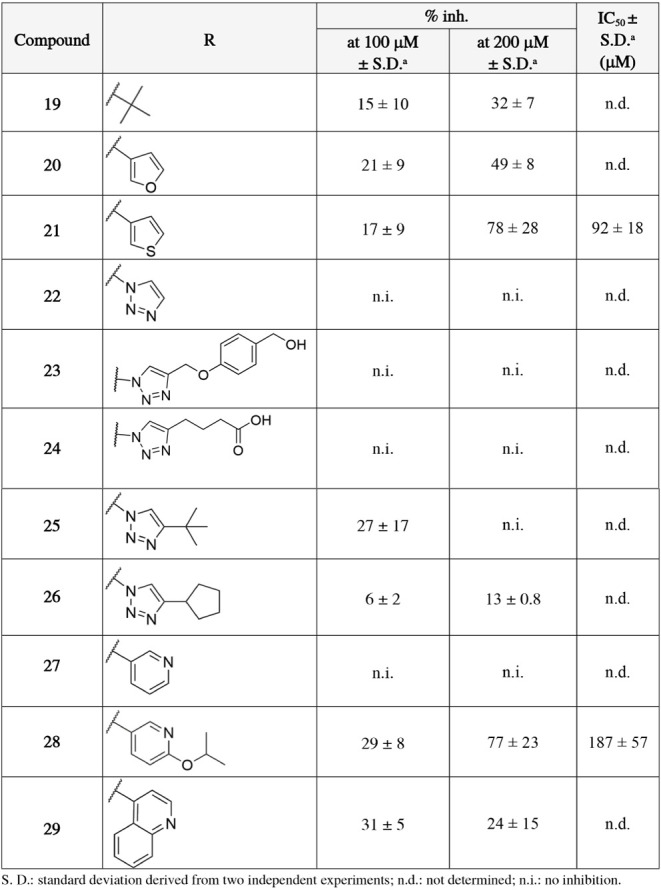
Inhibitory Potency and IC_50_ Determination of **19–29** in the Whole-Cell Uptake Assay with *Lactobacillus Casei* and Radioactively Labeled Substrate

### Exploration of 1,4-Triazole-Pyridinyl Hybrids

The second round of the SAR exploration was around compound **18** as the most active disubstituted derivative with an IC_50_ of 4 ± 1.5 μM. In analogy to what we did earlier, we experimentally assessed the kinetic solubility of **18** at pH 7.4, which showed a slightly worse profile than **3** (**18**, S = 108 ± 3 μM, logD_7.4_ 4.66 vs **3**, S > 200). To address this issue, we decided to explore an asymmetric disubstitution pattern where we kept the 3-chloro-2-isopropoxypyridine constant on one side while introducing a differently substituted triazole on the other side.^18^ Despite the limited activity observed for compounds **22**–**26** containing a triazolyl core (Table 3), we decided to reintroduce this ring owing to its favorable characteristics. The 1,2,3-triazolyl moiety is less lipophilic than many other aromatic or heteroaromatic rings, possesses good metabolic stability, and allows further functionalization, thereby promoting structural diversity.^17,19,20^

The library of asymmetrically substituted derivatives that we made comprises compounds **30**–**36** functionalized at position 4 of the triazolyl ring. Specifically, we introduced alicyclic (**30**–**32**), a tertiary amine (**35**), a branched alkyl group (**36**) as well as heteroaromatic rings (**33** and **34**) to gain insights into the impact of the substituents on the metabolic stability and the physicochemical properties. We screened our 1,4-triazole-pyridinyl derivatives at a concentration of 12.5 μM in the whole-cell-uptake assay, and the IC_50_ value was determined for compounds showing >60% inhibition at this concentration (Table 4). We were pleased to see that derivatives **30**–**34** and **36** exhibited IC_50_ values in the single-digit micromolar range, with **35** bearing the dimethyl amino group as the only inactive compound within this subset.

**Table 4:**
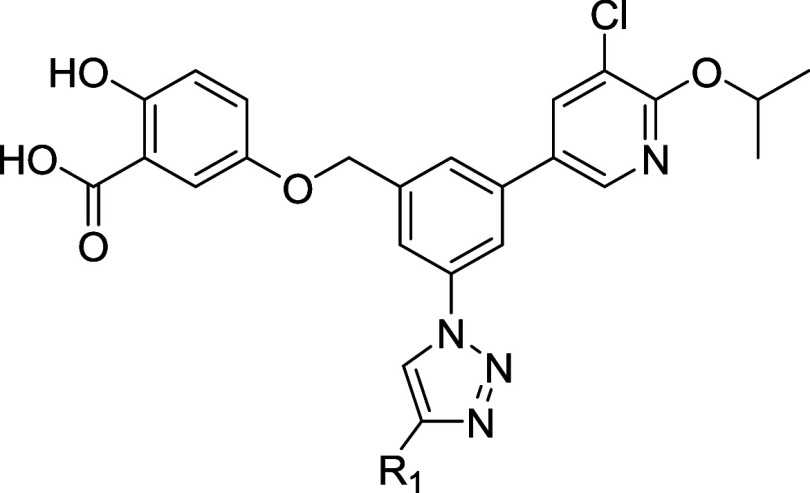

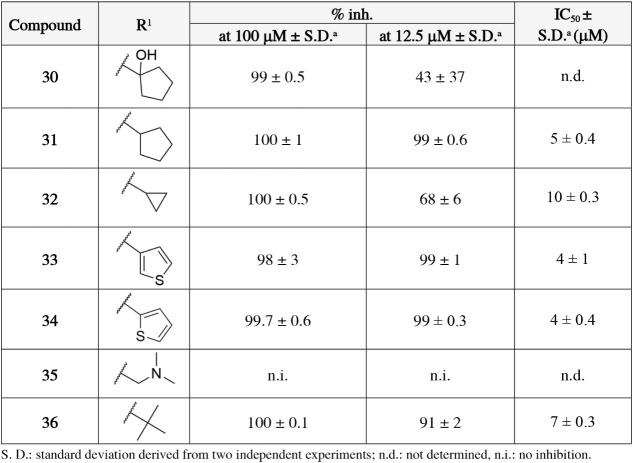
Inhibitory Potency and IC_50_ Determination of 30–36 in the Whole-Cell Uptake Assay with *Lactobacillus Casei* and Radioactively Labeled Substrate

### Docking Studies

To elucidate the binding mode of this series of compounds and exclude the involvement of the S-component, we docked **1**, **18**, **33**, and **36** into the pantothenate-binding pocket of the ECF transporter (ECF-PanT).^21^ Protein preparation, choice of the binding sites, and further details of the docking studies are described in the Supporting Information, Section 2.0. We began the docking investigation by building on our extensive former studies.^9,10^ Previously, the unbiased coarse-grained molecular-dynamics simulation ran on *L. delbrueckii* ECF-FolT2 and ECF-PanT suggested that **1** might bind at the interface between S-component and ECF-module, potentially interfering with the protein–protein interface.^9^ Following on that and to further evaluate the goodness of fit of the structural modifications introduced in the new compounds (**18**, **33**, and **36)**, we are presenting here once again the interactions formed by **1**. As reported in Figure 4A, compound **1** established a series of crucial interactions on the surface of the S-component (key salt bridge with Arg77, π–π stacking interactions with Phe28, and hydrophobic interactions with Leu24) as well as additional hydrophobic interactions with residues of the EcfT module (interactions with Thr28, Ala163, and Leu164).

**Figure 4:**
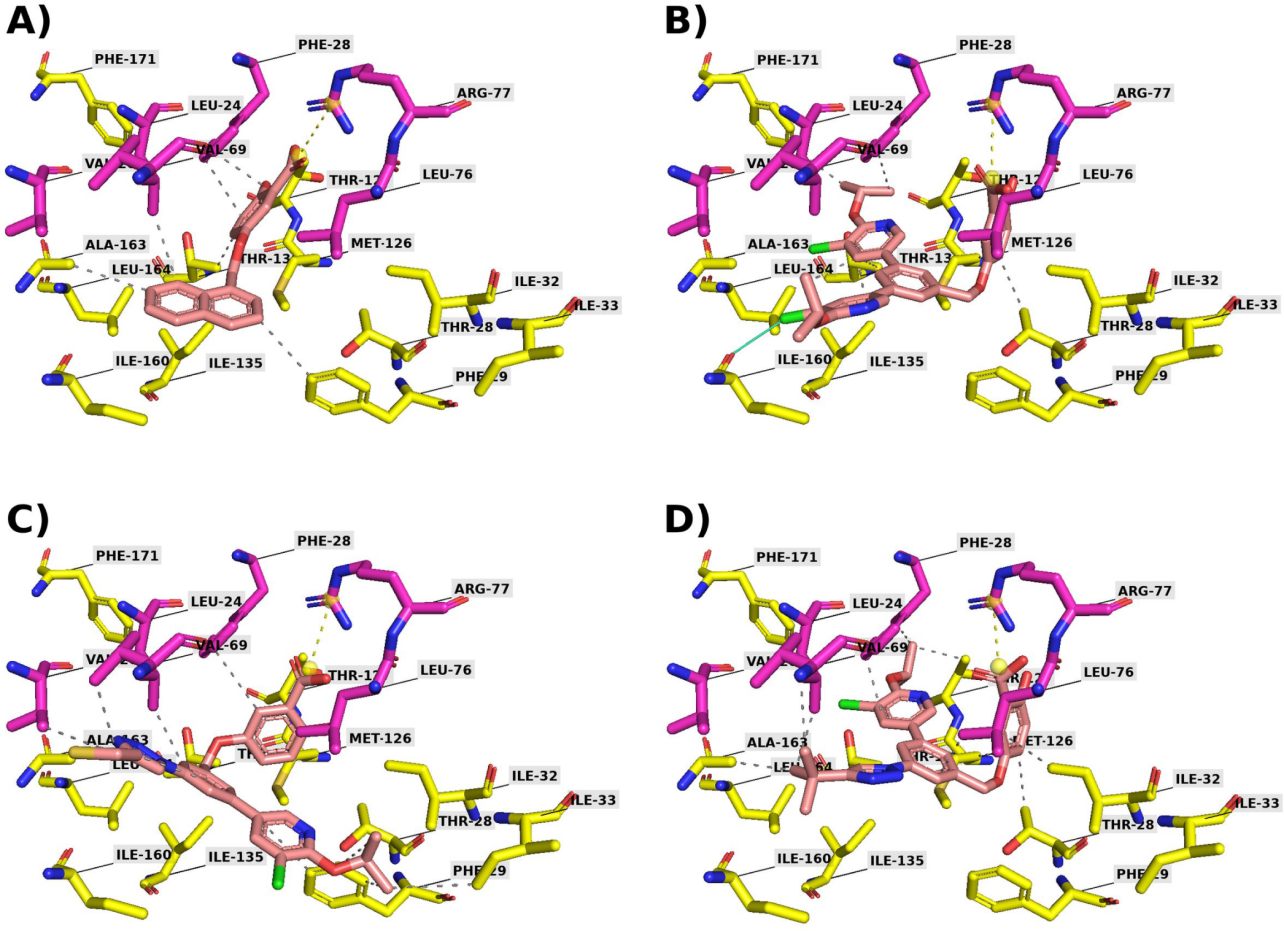
Docking studies by comparing the reference ligand **1** (A) with compounds **18** (B), **33** (C), and **36** (D), PDB ID: 6ZG3. Color code: ligands are in pink, S-component (chain C) is shown in magenta, and the EcfT module is yellow-colored. Salt-bridge contacts to Arg77 are highlighted with dashed, yellow lines, while hydrophobic contacts are indicated by dashed, gray lines, and hydrogen bonds are shown as blue lines.

Moving onward with the three new compounds (**18**, **33**, and **36**), we were pleased to see that all the selected poses were still predicted to preserve the interaction with Arg77, while the enhanced binding affinity are given by additional peripheral residues mainly in the ECF-T module, as shown in Figure 4B–D. Specifically, the chlorine atom of the 3-chloro-4-isopropoxypyridine in compound **18** formed a halogen bond with Ile160 and previously unexplored residues, including hydrophobic interactions with Thr28, Met126, and Phe171, are engaged. This expansion of the molecule toward new residues enables the ligand to be further anchored within the EcfT module (Figure 4B). Then, regarding compound **33**, we can see that it maintained existing hydrophobic interactions with Phe28 and Leu24 in the S-component and, at the same time, the thiophenyl ring is able to establish further hydrophobic interactions with residues Val20, Val69, and Leu76 always in the S-component (Figure 4C). Importantly, the compound also grows further within the EcfT module by establishing strong hydrophobic interactions with Phe29 and Ile33. Intriguingly, the triazolyl core connected to a tertiary butyl group in compound **36** formed dual hydrophobic interactions with Leu24 from the S-component and Ala163 within the EcfT module (Figure 4D). The isopropoxy substituent from the 3-chloro-4-isopropoxypyridine interacts with the previously unexplored Thr127 residue in the EcfT module while maintaining the hydrophobic interactions with Phe28 of the S-component. The central benzylic ring established hydrophobic interactions with Leu164 and Ile135. Overall, based on the docking study, the structural expansions in the three analyzed compounds allowed to explain that the boost in potency is due to additional ligand-target interaction, that exploit both S-component and EcfT module residues.

### Antibacterial Potency, Confirmation of On-Target Activity, and Time-Kill Kinetics of Selected Frontrunner Molecules

The most potent inhibitors identified through our extensive SAR study were evaluated for their antimicrobial profile against a panel of Gram-positive pathogens, including the resistant strains *S. pneumoniae* *DSM-11865* (penicillin-resistant; PRSP) and *E. faecium* *DSM-17050* (vancomycin-resistant; VRE). As reported in Table 5, by comparing the newly synthesized inhibitors **18**, **31**, **33**, **34** and **36** with the starting point of this study **3**, a clear improvement of up to 10-fold in antimicrobial activity was observed, reaching Minimum Inhibitory Concentration (MIC) values <1 μg/mL. By comparing the functional activity in the whole-cell uptake assay data with the MIC values, we observed a trend. In fact, the increasing potency also results in reduced MIC against most of the pathogens tested (Table S2 and Figure S2).

**Table 5:** Minimum Inhibitory Concentration (MIC) Determination against a Panel of Gram-Positive Bacteria and Proteoliposome Uptake Assay with *Lactobacillus delbrueckii* and Radiolabeled Substrate

	**MIC (μg/mL)**					**Proteoliposome uptake assay**	
Cmpd	*E. faecalis* *DSM-20478*	*E. faecium* *DSM-17050* ^a^	*E. faecium* *DSM-20477*	*S. pneumoniae* *DSM-11865* ^b^	*S. pneumoniae* *DSM- 20566*	ECF-FolT2% inh. at 150 μM ± S.D.	ECF-PanT % inh. at 150 μM ± S.D.
**3**	11	3–6	11	6	1–3	29.2^d^	65.4^d^
**18**	2–5	5–9	4.5	1–2	2	96.2	99.7
**31**	1–2	1–2	2	0.5	0.5–1	101	n.d.
**33**	1–2	2–4.5	1–2	0.3–0.6^c^	0.3–0.6^c^	74.7 (IC_50_= 69 μM)	97.5
**34**	2	2	2	1^c^	0.6	n.d.	n.d.
**36**	2–4	2	1–2	1	0.5–1	81.1 (IC_50_= 42.84 μM)	100.3

a VRE: Vancomycin-resistant *Enterococcus*.

b PRSP: Penicillin-resistant *Streptococcus pneumoniae*; S.D.: standard deviation derived from two independent experiments.

c Not fully inhibited; n.d. not determined.

d Measured at 100 μM.

As a next step, we tested the most promising on-target inhibitors in the proteoliposome uptake assay using ECF transporters from *Lactobacillus delbrueckii* for the transportation of radioactively labeled folic (ECF-FolT2) and pantothenate (ECF-PanT) acids.^21^ This assay was designed to further confirm that we are targeting group-II ECF transporters and to provide insight into the allosteric mode of action of our chemical class of compounds, which are able to inhibit the uptake of ECF-FolT2 and ECF-PanT in a similar manner (Table 5). At a concentration of 150 μM, analogues **18**, **31**, **33**, and **36** showed an inhibition range of 74–100%, which is a comparable inhibition profile to that found in the whole-cell uptake assay. Nevertheless, it should be noted that a comprehensive comparison between the two assays is difficult to draw because the target proteins used are not identical, and there are inherent assay differences.^7,21^ Further, to learn more about the antibacterial activity following inhibition of ECF transporters, we selected analogue **36** due to its high antibacterial potency and efficient target inhibition as a representative molecule and studied its time-kill kinetics in the multidrug-resistant model organism *E. faecium* ATCC51559. The MIC of **36** against this strain was determined as 8.6 μg/mL, and time-kill kinetics were assessed over 24 h at the 2-, 4-, and 8-fold MIC in comparison to ciprofloxacin (CIP) that showed a similar MIC against this strain (8 μg/mL) (Figure 5).

**Figure 5:**
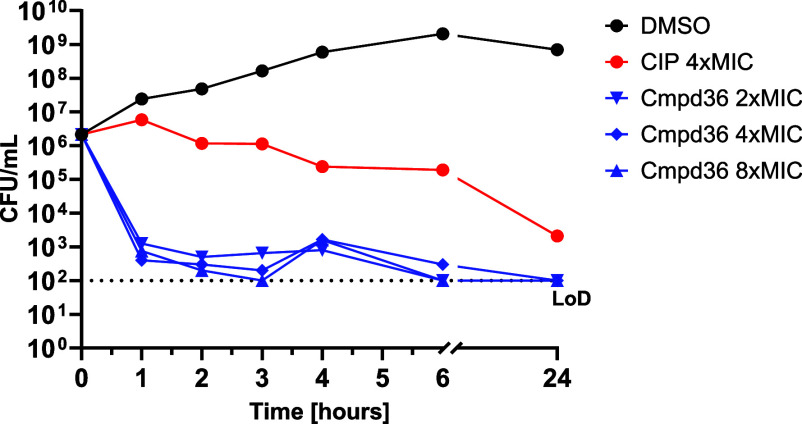
Time-kill curve (TKC) of *Enterococcus faecium* treated with **36** at 2×, 4×, and 8× MIC. DMSO served as solvent/growth control (1%, *v/v*) CIP at 4x MIC was used as the reference antibiotic. LOD: limit of detection (10^2^ CFU/mL).

Intriguingly, **36** exhibits rapid bactericidal killing of *E. faecium* ATCC51559 already after 1 h at all tested concentrations, followed by sustained bactericidal activity with more than 3-log reduction of colony-forming units (CFU/mL) within the 24-h observation period. 2x MIC of **36** is determined as the minimum bactericidal concentration, which corresponds to a tested concentration of 17.2 μg/mL.

### *In Vitro* ADMET Profiling

As anticipated, a goal of this work was the optimization of the drug-like properties of compound **3**. In order to understand whether our synthetic work resulted in a clear improvement of the *in vitro* ADMET profile, we made a direct comparison of **3** with compounds **18**, **31**, **33,** and **36** as the most potent ones in the series. As reported in Table 6, compound **3** undergoes rapid metabolism in mouse (Cl_int_: 27 ± 14 μL/min/mg) and human liver (Cl_int_: 264 ± 183 μL/min/mg) microsomes. Nevertheless, we were pleased to see that the newly synthesized derivatives showed an increased half-life in both murine and human microsomes (Table 6) and S9 fraction (Table S3) with **18**, **31**, **33**, and **36** being fully stable over 2 h. Notably, compound **36**, despite bearing a *tert*-butyl group as compound **3**, showed a higher half-life that could be attributed to the reduced logD_7.4_ and higher S values (1.73 and >200 μM, respectively). Additionally, the compounds do not show stability issues in plasma. Furthermore, we determined their toxicity against HepG2 cells and did not observe any notable difference from the already moderate cytotoxicity observed for **3** and calculated the selectivity index (SI). Compound **3** showed moderate cytotoxicity against A549 cells (CC_50_ = 59 ± 6 μM), while the newer derivatives (**18**, **31**, **33**, **36**) had a low or no impact on A549 viability. Next, we also assessed the effects of a series of compounds on the potassium (hERG-K^+^) cardiac ion channel. At a concentration of 10 μM, all the compounds tested did not show any inhibition (Table 6).

**Table 6:** *In Vitro* ADMET Profile for Frontrunners

	**Compound**				
Parameters	3	18	31	33	36
logD_7.4_ ^a^	–0.43	4.66	2.01	3.40	1.73
Kinetic Solubility 1% DMSO/PBS [μM]	>200	108 ± 3	115 ± 3	116 ± 11	>200
HepG2 CC_50_ ^b^ [μM]	75 ± 22	93 ± 47	92 ± 31	88 ± 18	91 ± 25
MIC *E. faecium* DSM-20477 [μM]	30.9	7.7	3.7	2.7	2.8
SI CC_50 HepG2_/MIC_*E faecium*_	2.4	12.1	25.2	32.6	32.5
A549 viability at 100 μM [%]	59 ± 6 μM(CC_50_ ^b^)	70 ± 8	99 ± 19	83 ± 13	81 ± 19
Mouse Liver Microsomes *t*_1/2_ ^c^ [min]/Cl_int_ ^d^ [μL/min/mg]	59 ± 24/27 ± 14	>120/ <11.6	>120/<11.6	>120/<11.6	>120/<11.6
Human Liver Microsomes *t*_1/2_ ^c^[min]/Cl_int_ ^d^ [μL/min/mg]	7.6 ± 4.3/264 ± 183	>120/ <11.6	>120/<11.6	>120/<11.6	124 ± 11/11 ± 1
Mouse Plasma *t*_1/2_ ^b^ [min]	>240	>240	>240	>240	>240
Human Plasma *t*_1/2_ ^b^ [min]	>240	>240	>240	>240	>240
CiPA hERG-K^+^ ^e^ (% inh. @ 10 μM)	–14.8	–11.0	–3.8	n.d.	–6.8

a LogD_7.4_ was determined *via* chromatography.

b CC_50_ is the concentration at which cell viability is reduced by 50%.

c Half-life.

d Intrinsic clearance. Means ± standard deviation are shown for at least 2 independent experiments. SI (selectivity index) = CC_50_/MIC_*E. faecium*_.

e Verapamil has been used as a reference compound.

Taken together, these findings highlight that based on our multiparameter approach, we were able to improve both potency and metabolic stability while maintaining the good solubility of the initial compound, paving the way for further development toward a lead compound inhibiting ECF transporters.

### *In Vivo* Activity of Optimized ECF Inhibitors in *G. mellonella* (Greater Wax Moth) and *Danio rerio* (Zebrafish) Infection Models

Next, we investigated the effects of the ECF transporter inhibitors, which demonstrated an optimal balance between their inhibitory activity, *in vitro* ADMET profile, and their cytotoxicity profile on the survival of *G. mellonella* larvae infected with *S. pneumoniae*. Larvae were injected with a combination of an overnight culture of *S. pneumoniae* DSM-20566 and the compounds at a concentration of 50 μM, and we observed their survival for a period of 72 h. Under these conditions, the selected inhibitors **18**, **31**, **33,** and **36** showed a good inhibitory effect against this *S. pneumoniae* strain (Figure 6). We chose compounds **18** and **36** to assess toxicity at 200 μM and observed no effect (Figure S3).^22^

**Figure 6:**
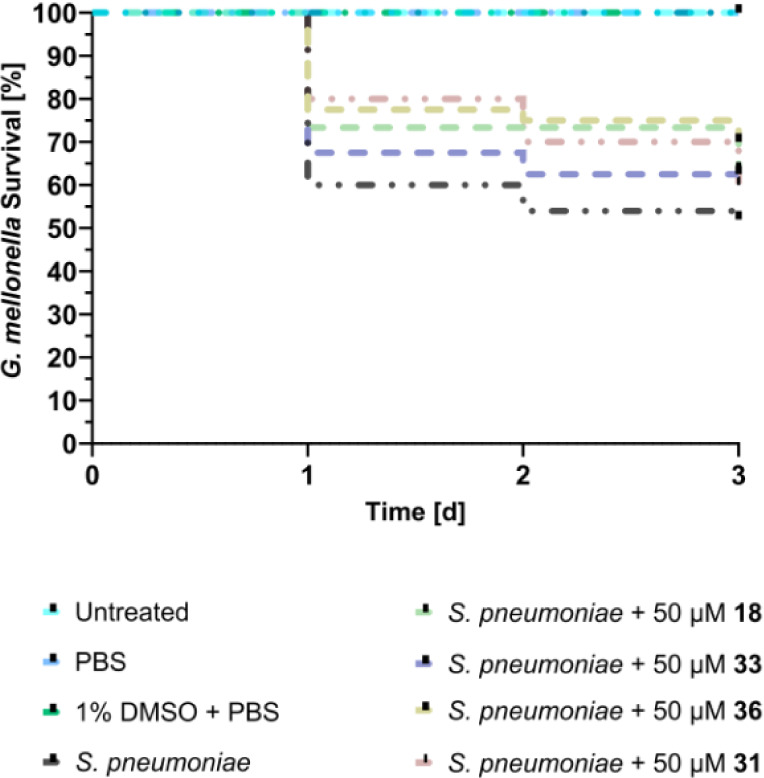
Simple survival analysis (Kaplan–Meier) was performed using GraphPad Prism. The *Galleria mellonella* larvae were injected with *Streptococcus pneumoniae* at an OD_600_ of 1.5 in the absence and presence of compounds (**18**, **31**, **33,** and **36**) at 50 μM. The control groups were injected with PBS or left untreated (no injection) and 1% DMSO.

Our findings showed that *S. pneumoniae* with an OD_600_ of 1.5 led to a reduction in larval survival to 50% after 3 days of incubation at 37 °C and 5% CO_2_ (Figure 6). Conversely, negative controls untreated with compounds, PBS, and 1% DMSO exhibited no impact on survival after the same duration. Treatment with 50 μM of compound **33** increased larval survival by 12%. Similarly, compounds **18** and **33** demonstrated an increase in survival up to 65%.

Given the superior bactericidal killing of *E. faecium* by **36** and its overall favorable properties, we set out to additionally assess this molecule in a vertebrate model. For this, we used zebrafish (*Danio rerio*) embryos that were infected at 2 dpf (days postfertilization) with 2 × 10^5^ CFU of *E. faecium* ATCC51559 through micro-injection into the caudal vein. Given the favorable data from the cytotoxicity evaluation in HepG2 cells (CC_50_ in the range of 100 μM), we were not particularly concerned about potential toxicity issues. Indeed, prior to testing of **36** in the Zebrafish infection model, we assessed potential *in vivo* toxicity up to a concentration of 1000 μM without observing any effect on embryo survival. However, precipitation was observed at the highest test concentrations, and we therefore used 50 μM of **36** in the infection model to make sure all compound was in solution. Infected embryos were then treated with 50 μM **36** through waterborne exposure. Survival rates were monitored daily up to 3 dpt (days post-treatment) with daily renewal of dosing solution. While survival of nontreated embryos decreased to 30%, treatment with linezolid (400 μM) as a reference drug increased survival to 90%. The ECF inhibitor **36** was not as effective as linezolid but significantly increased the survival rate to 40%. It should be noted however that this might be due to the fact that the concentration of **36** (50 μM) could not be increased further in the chosen setup, and it was well below the concentration used for linezolid (400 μM). The use of 50 μM concentration of **36** led to a statistically significant effect on survival (30% vs 40% survival in vehicle vs treated group), and we assume that better efficacy can be achieved by systemic compound administration (which cannot be easily implemented in the used zebrafish model) and by the use of a suitable formulation that helps solubilize the compound. Noteworthy, the zebrafish model used has some limitations when it comes to compound administration (waterborne exposure requires a compound to either passively diffuse and/or to be orally available) and dose limitation (compound from DMSO stock solution could only be added up to 50 μM to prevent precipitation). Thus, in general, we are encouraged by our initial findings in *Galleria* and zebrafish models, as these provide evidence that ECF frontrunners can be successfully tested in more advanced murine models in the future, while a preformulated compound for systemic administration would be preferred.

## Conclusions

In summary, we have conducted an extensive SAR study starting from **3** as our previously reported inhibitor series that suffered from poor metabolic stability given by its bis-*tert*-butyl moiety. We addressed that point exploiting a detailed SAR study with a subset of 31 novel compounds (**5**–**36**) where the introduction of the 1,4-triazolyl ring turned out to be particularly recommended.

The use of click chemistry as a versatile approach, led to a subset of molecules where the weakness of our starting point was overcome, as demonstrated by the superior profile of compounds **31**, **33,** and **36** in the *in vitro* ADME-T study. In addition, these new compounds possess a submicromolar cell activity in a panel of Gram-positive bacteria while keeping the on target activity. The boost in potency has been rationalized by docking studies, which reveal were additional ligand-target interactions within the ECF module and S-component. For the first time, we studied the time-kill kinetics over 24 h in *E. faecium* and found bactericidal activity for compound **36**. Then, we confirmed the activity of the frontrunner compounds in *Galleria mellonella* (greater wax moth) as previously reported for compounds **3** and **4**, but here we also checked their behavior in zebrafish infection models. Even though no superb efficacy has been observed, our study sets the stage for the future development of this chemical class. The statistically significant effect between the vehicle-treated and **36**-treated groups provides evidence that compound **36** targets the ECF transporters and does *in vivo* activity.

The results described above represent an important milestone in the development of anti-infectives targeting the ECF transporters as well as marking a significant advance in the field of AMR.

Our future work will focus on further optimization of the presented inhibitors with respect to their potency and *in vivo* efficacy.

## Experimental Section

This research did not involve human or animal participants.

All experiments involving zebrafish (*Danio rerio*) were performed on embryos and larvae younger than 120 h postfertilization (hpf). At this developmental stage, zebrafish are not considered protected animals under applicable ethical regulations (Directive 2010/63/EU) because they have not yet developed fully functional nervous systems or the capacity for independent feeding. Accordingly, no formal ethical approval was required. All procedures were conducted in accordance with institutional guidelines and the principles of the 3Rs (Replacement, Reduction, and Refinement). Embryos were maintained under optimal conditions, and humane end points were applied.

### Chemicals, Materials, and Methods

NMR experiments were run on a Bruker Avance Neo 500 MHz spectrometer. Spectra were acquired at 300 K, using deuterated solvents. Chemical shifts for ^1^H and ^13^C spectra were recorded in parts per million (ppm) using the residual nondeuterated solvent as the internal standard. Coupling constants (*J*) are given in Hertz (Hz). Data are reported as follows: chemical shift, multiplicity (s = singlet, d = doublet, t = triplet, m = multiplet, br = broad and combinations of these), coupling constants, and integration. Flash chromatography was performed using the automated flash chromatography system CombiFlash Rf+ (Teledyne Isco, Lincoln, NE, USA), equipped with RediSepRf silica columns (Axel Semrau, Sprockhövel Germany). TLC was performed with aluminum-backed silica TLC plates (Macherey–Nagel MN ALUGRAM Sheets SIL G/UV 254 20 × 20 cm, 818133) with a suitable solvent system and was visualized using UV fluorescence (254 and 365 nm). All reactions were carried out in oven-dried glassware under an atmosphere of argon. Anhydrous DMF was purchased from Aldrich and used directly.

Liquid chromatography–mass spectrometry was performed on an LC-MS system, consisting of a Dionex UltiMate 3000 pump, autosampler, column compartment and MWD or DAD detector (Thermo Fisher Scientific, Dreieich, Germany) and an ESI quadrupole MS (MSQ Plus or ISQ EC, Thermo Fisher Scientific, Dreieich, Germany). Columns used: 1) Hypersil Gold column, 100 × 2.1 mm, 3 μm. At a flow rate of 700 μL/min, the gradient of H_2_O (0.1% FA) and ACN (0.1% FA) starting from 5% ACN and then increased to 100% over 7 min. 2) Hypersil Gold column, 100 × 1.9 mm, 2.1 μm. At a flow rate of 600 μL/min, the gradient of H_2_O (0.1% FA) and ACN (0.1% FA) starting from 5% ACN and then increased to 100% over 5.5 min. The mass spectrum was measured in positive and negative mode in a range from 100–600 *m*/*z*. The UV spectrum was recorded at 254 nm. High-resolution mass spectra (HR-MS) were recorded with a ThermoScientific system where a Dionex Ultimate 3000 RSLC was coupled to a Q Exactive Focus mass spectrometer with an electrospray ion (ESI) source. An Acquity UPLC BEH C8, 150 × 2.1 mm, 1.7 μm column equipped with a VanGuard Pre-Column BEH C8, 5 × 2.1 mm, 1.7 μm (Waters, Germany) was used for separation. At a flow rate of 250 μL/min, the gradient of (A) H_2_O + 0.1% FA and (B) ACN + 0.1% FA was held at 10% B for 1 min and then increased to 95% B over 4 min. It was held there for 1.2 min before the gradient was decreased to 10% B over 0.3 min, where it was held for 1 min. The mass spectrum was measured in positive and negative mode in a range from 120–1000 *m*/*z*. UV spectrum was recorded at 254 nm.

Preparative RP-HPLC was performed using an UltiMate 3000 Semi-Preparative System (Thermo Fisher Scientific) with nucleodur C18 Gravity (250 mm × 16 mm, 5 μm). Separation was done using gradient 5–100% CH_3_CN + 0.05% HCOOH in water +0.05% HCOOH in 53 min at a flow rate of 10 mL/min and end with a 5 min step at 100% CH_3_CN. The sample was dissolved in DMSO and manually injected into the HPLC system. All compounds are >95% pure by HPLC analysis.

### General Procedures

#### General Procedure A (GP-A): Ether Synthesis

A solution of methyl-2,5-dihydroxybenzoate (1.0 equiv) and K_2_CO_3_ (1.2 equiv) was stirred in acetone (0.3 M) for 30 min at room temperature, and then the corresponding benzyl bromide (1.0–1.1 equiv) was added. The reaction mixture was stirred at room temperature until LC-MS analysis showed completion of the reaction. The solution was concentrated under vacuum, the residue was suspended in H_2_O/EtOAc (1:1), and the aqueous phase was extracted with ethyl acetate (3×). The combined organic layers were dried over MgSO_4_, filtered, concentrated *in vacuo,* and purified by flash chromatography.

#### General Procedure B (GP-B): Basic Ester Hydrolysis

To a stirred solution of ester (1.0 equiv) in CH_3_OH (0.2 M), 10% aq. NaOH (10 equiv) was added. The solution was stirred at 65 °C for 12 h until LC-MS analysis showed the complete consumption of the starting material. Then, the solvent was reduced under vacuum, and the residue was dissolved in EtOAc. Then, a solution of HCl 2 M was added to reach pH 2–3, and the aqueous phase was extracted with EtOAc (3×). The combined organic layers were dried over MgSO_4_, filtered, concentrated *in vacuo,* and purified by flash chromatography.

#### General Procedure C (GP-C): Suzuki Miyara Cross-Coupling

In a microwave sealed-tube, the aryl halide (1.0 equiv.), the organoboron specie (1.2−2.5 equiv., as stated), K_2_CO_3_ (3.0−5.0 equiv.) and [Pd­(PPh_3_)_4_] or [Pd­(dppf)­Cl_2_] as stated (0.05 equiv.) were placed under vacuum and flushed with N_2_. The mixture was dissolved in 1,4-dioxane/H_2_O (6:1, 0.2 M), and the reaction was stirred in microwave at 90 °C for 1 h. After completion of the reaction, the 1,4-dioxane was removed under vacuum, and the aqueous phase was diluted with H_2_O and extracted with EtOAc (3×). The combined organic layers were dried over MgSO_4_, filtered, concentrated in vacuo and purified by flash chromatography.

#### General Procedure D (GP-D): Bromination of Benzylic Alcohol

Under argon atmosphere, the corresponding alcohol (1.0 equiv.) was dissolved in DCM (0.3 M) and PBr_3_ (5.0 equiv.) was added at 0 °C. After 10 min, pyridine was added, and the reaction mixture was stirred at rt overnight. Once the reaction was completed, the mixture was cooled to 0 °C and quenched with H_2_O. The aqueous phase was extracted with DCM (3×). The combined organic layers were dried over MgSO_4_, filtered, concentrated in vacuo and purified by flash chromatography.

#### General Procedure E (GP-E): Nitro Reduction

To a suspension of the corresponding nitro compound (1.0 equiv.) in ethanol/H_2_O (3:1, 0.2 M) iron powder (6.0 equiv.) was added at refluxing temperature. After 20 min, NH_4_Cl (6.0 equiv.) was added, and the mixture was stirred for 1 h at reflux. Once the reaction was completed, the mixture was filtered over celite to remove iron residue and washed several times with EtOAc. The filtrate was partitioned with saturated Na_2_CO_3_, and the basic layer was further extracted with ethyl acetate (3×). The combined organic layers were dried over MgSO_4_, filtered, concentrated in vacuo and purified by flash chromatography.

#### General Procedure F (GP-F): Amine-to-Azide Conversion

To a solution of the amine (1.0 equiv.) in 1,4-dioxane/HCl 6 M (0.15 M), NaNO_2_ (3 equiv.) was added at 0 °C, and the mixture was stirred for 20 min. Then, NaN_3_ (4 equiv.) was added slowly at 0 °C and the reaction was stirred at room temperature for 30 min. After removal of 1,4-dioxane under vacuum, the aqueous phase was neutralised with saturated (sat.) Na_2_CO_3_ solution and then extracted with ethyl acetate (3×). The combined organic layers were dried over MgSO_4_, filtered, concentrated in vacuo and used in the next step without further purification.

#### General Procedure G (GP-G): Copper-Catalyzed Azide–Alkyne Cycloaddition (CuAAC)

The azide (1.0 equiv.) and alkyne (1.0 equiv.) derivatives were dissolved in (THF/H_2_O: 2:1, 0.4 M). Sodium ascorbate (0.4 equiv.) was added, followed by the addition of copper (II) sulfate pentahydrate (0.2 equiv.). The resulting reaction was vigorously stirred for 20 min –3 h at room temperature. Then, THF was removed under vacuum, and the mixture diluted with EtOAc. The aqueous phase was acidified with HCl (2 M) until pH 2–3 and extracted with EtOAC (3×). The organic solvent was dried over MgSO_4_ and evaporated under reduced pressure. Purification was done by semi-preparative HPLC.

### Synthesis and Characterization

#### 5-((3,5-*di*Chlorobenzyl)­oxy)-2-hydroxybenzoic Acid (5)

According to **GP-B**, using **42** (200 mg, 0.66 mmol) and NaOH (in aq. 10%, 0.8 mL) afforded after purification by flash chromatography (c-Hex/EtOAc 90:10) **5** as a white solid (170 mg, 90%). ^**1**^ **H NMR** (500 MHz, DMSO-*d*_6_) δ 7.65 (t, 1H, *J* = 1.8 Hz), 7.50 (s, 2H), 7.33 (d, 1H, *J* = 3.2 Hz), 6.98 (br, 1H, *J* = 6.3 Hz), 6.68 (d, 1H, *J* = 9.0 Hz), 5.04 (s, 2H). ^**13**^ **C NMR** (126 MHz, DMSO-*d*_6_) δ 171.6, 157.0, 149.6, 142.5, 134.5, 127.7, 126.4, 121.5, 117.4, 115.1, 68.7. **HR-MS** calcd for C_14_H_9_Cl_2_O_4_ [*M*-H]^−^:310.9883, found 310.9886.

#### 5-((3-Bromo-5-chlorobenzyl)­oxy)-2-hydroxybenzoic Acid (6)

According to **GP-B**, using **43** (200 mg, 0.66 mmol) and NaOH (sol. aq. 10%, 0.8 mL) afforded, after purification by flash chromatography (c-Hex/EtOAc 90:10) **6** as an off-white solid (170 mg, 89%). ^**1**^ **H NMR** (500 MHz, DMSO-*d*_6_) δ 7.69 (t, 1H, *J* = 1.8 Hz), 7.64 (s, 1H), 7.55 (s, 1H), 7.35 (d, 1H, *J* = 3.2 Hz), 7.25 (dd, 1H, *J* = 3.1, 9.1 Hz), 6.92 (d, 1H, *J* = 9.0 Hz), 5.09 (s, 2H). ^**13**^ **C NMR** (126 MHz, DMSO-*d*_6_) δ 171.9, 156.2, 150.6, 142.2, 134.7, 130.5, 129.4, 126.9, 124.7, 122.8, 118.7, 114.4, 113.2, 68.6. **HR-MS** (ESI) calcd for C_14_H_9_BrClO_4_ [*M*-H]^−^: 354.9378, found 354.9368.

#### 5-((3,5-*di*Bromobenzyl)­oxy)-2-hydroxybenzoic Acid (7)

According to **GP-B**, using **44** (600 mg, 0.96 mmol) and NaOH (sol. aq. 10%, 1.70 mL) afforded after flash chromatography (c-Hex/EtOAc 88:12) **7** as an off-white solid (550 mg, 95%). ^**1**^ **H NMR** (500 MHz, DMSO-*d*_6_) δ 10.90 (s, 1H), 7.80 (t, *J* = 1.7 Hz, 1H), 7.68 (d, *J* = 1.7 Hz, 2H), 7.34 (d, *J* = 3.2 Hz, 1H), 7.24 (dd, *J* = 9.0, 3.2 Hz, 1H), 6.92 (d, *J* = 9.0 Hz, 1H), 5.08 (s, 2H). ^**13**^ **C NMR** (126 MHz, DMSO-*d*_6_) δ 171.4, 155.7, 150.1, 141.9, 132.6, 129.3, 124.2, 122.5, 118.2, 113.9, 112.7, 68.1. **HR-MS** (ESI) calcd for C_14_H_9_Br_2_O_4_ [*M*+H]^+^: 398.8873, found 398.8852.

#### 5-((3,5-Bis­(trifluoromethyl)­benzyl)­oxy)-2-hydroxybenzoic Acid (8)

According to GP-B, using **45** (600 mg, 0.96 mmol) and NaOH (in aq. 10%, 1.70 mL) afforded after preparative HPLC (90% ACN) **8** as a pale yellow solid (550 mg, 96%). ^**1**^ **H NMR** (500 MHz, DMSO-*d*_6_) δ 8.18 (s, 2H), 8.10 (s, 1H), 7.41 (d, 1H, *J* = 3.2 Hz), 7.30 (dd, 1H, *J* = 3.2, 9.0 Hz), 6.94 (d, 1H, *J* = 9.0 Hz), 5.28 (s, 2H). ^**13**^ **C NMR** (126 MHz, DMSO-*d*_6_) δ 171.9, 156.3, 150.6, 141.2, 130.8 (q, *J* = 33.1 Hz), 128.7 (d, *J* = 3.7 Hz), 124.7, 122.0 (br t, *J* = 5.5 Hz), 123.79 (q, *J* = 273.0 Hz), 118.7, 114.5, 113.2, 68.8. ^**19**^ **F NMR** (470 MHz, DMSO-*d*_6_) δ −61.29, −208.27. **HR**-**MS** (ESI) calcd for C_16_H_9_F_6_O_4_ [*M*–H]^−^: 379.0410, found 379.0411.

#### 5-((3,5-*di*Cyclopropylbenzyl)­oxy)-2-hydroxybenzoic Acid (9)

According to **GP-C**, using **7** (60 mg, 0.15 mmol), cyclopropylboronic acid (44 mg, 0.34 mmol), K_3_PO_4_ (143 mg, 0.68 mmol), PCy_3_ (4.2 mg, 0.01 mmol, 0.1 equiv) and [Pd­(OAc)_2_] (1.3 mg, 0.007 mmol, 0.05 equiv) in DMF/water (20:1, 0.8 M) for 48 h at 80 °C, afforded after preparative HPLC (90% ACN) **9** as an off-white solid (8 mg, 16%). ^**1**^ **H NMR** (500 MHz, DMSO-*d*_6_) δ 7.33 (t, *J* = 6.5 Hz, 1H), 7.20 (dd, *J* = 9.0, 3.2 Hz, 1H), 6.89 (d, *J* = 8.9 Hz, 3H), 6.73 (s, 1H), 4.94 (s, 2H), 1.87 (tt, *J* = 10.0, 5.1 Hz, 2H), 0.95–0.88 (m, 4H), 0.68–0.61 (m, 4H). ^**13**^ **C NMR** (126 MHz, DMSO-*d*_6_) δ 171.6, 155.5, 150.6, 143.7, 136.9, 124.2, 122.11, 121.75, 118.07, 113.86, 112.70, 70.02, 14.98, 9.27. **HR-MS** (ESI) calcd for C_20_H_19_O_4_ [*M*–H]^−^: 323.1288, found 323.1282.

#### 5-((3,5-Bis­(2,2,2-trifluoroethyl)­benzyl)­oxy)-2-hydroxybenzoic Acid (10)

According to **GP-C**, using **7** (90 mg, 0.225 mmol) (2,2,2-trifluoroethyl)­boronic acid (78 mg, 1.35 mmol), K_2_CO_3_ (187 mg, 0.92 mmol,) and [Pd­(PPh_3_)_4_] (10 mg, 0.009 mmol) in 1,4-dioxane/H_2_O (6:1, 0.2 M) under microwave irradiation at 90 °C for 1 h, afforded after preparative HPLC (90% ACN) **10** as a white solid (10 mg, 11%). ^**1**^ **H NMR** (500 MHz, DMSO-*d*_6_) δ 7.63 (s, 1H), 7.61 (s, 1H), 7.39 (d, 2H, *J* = 2.9 Hz), 7.24 (d, 1H, *J* = 8.6 Hz), 6.90 (d, 1H, *J* = 9.0 Hz), 5.10 (s, 2H), 2.9–2.9 (m, 2H), 2.6–2.7 (m, 2H). ^**13**^ **C NMR** (126 MHz, DMSO-*d*_6_) δ 171.9, 156.1, 151.0, 139.6, 137.7, 129.0, 128.2, 126.3, 127.7, 124.3, 118.4, 114.5, 113.7, 70.3, 34.3, 27.8. ^**19**^ **F NMR** (DMSO-*d*_6_, 470 MHz) δ −64.71, −64.73, −64.76. **HR**-**MS** (ESI) calcd for C_18_H_13_F_6_O_4_ [*M*–H]^−^: 407.0723, found 407.0704.

#### 5-((3,5-*di*(Furan-3-yl)­benzyl)­oxy)-2-hydroxybenzoic Acid (11)

According to **GP-C**, using **7** (80 mg, 0.19 mmol), furan-3-ylboronic acid (109 mg, 0.59 mmol), K_2_CO_3_ (61 mg, 0.44 mmol,) and [Pd­(PPh_3_)_4_] (12 mg, 0.01 mmol) in 1,4-dioxane/H_2_O (6:1, 0.2 M) under microwave irradiation at 90 °C for 1 h, afforded after preparative HPLC (72% ACN) **11** as a white solid (27 mg, 35%). ^**1**^ **H NMR** (500 MHz, MeOD) δ 7.97 (s, 2H), 7.68 (s, 1H), 7.56 (t, *J* = 1.6 Hz, 2H), 7.50 (dd, *J* = 10.1, 2.2 Hz, 3H), 7.21 (dd, *J* = 9.0, 3.2 Hz, 1H), 6.87 (d, *J* = 9.0 Hz, 3H), 5.07 (s, 2H). ^**13**^ **C NMR** (126 MHz, MeOD) δ 173.2, 157.8, 152.5, 145.1, 140.4, 139.8, 134.7, 127.5, 125.5, 124.6, 123.6, 119.1, 115.5, 113.8, 109.7, 71.7. **HR-MS** (ESI) calcd for C_22_H_15_O_6_ [*M*–H]^−^: 375.0874, found 375.0869.

#### 5-((3,5-Bis­(3-(methoxycarbonyl)­furan-2-yl)­benzyl)­oxy)-2-hydroxybenzoic Acid (12 a)

According to **GP-C**, using **7** (80 mg, 0.19 mmol) (3-(methoxycarbonyl)­furan-2-yl)­boronic acid (45 mg, 0.50 mmol), K_2_CO_3_ (61 mg, 0.48 mmol,) and [Pd­(PPh_3_)_4_] (12 mg, 0.01 mmol) in 1,4-dioxane/H_2_O (6:1, 0.2 M) under microwave irradiation at 90 °C for 1 h, afforded **12a** that was used directly in the next step without any purification.

#### 2,2’-(5-((3-Carboxy-4-hydroxyphenoxy)­methyl)-1,3-phenylene)­bis­(furan-3-carboxylic Acid) (12)

According to **GP-B**, using **12a** (200 mg, 0.668 mmol) and NaOH (sol. aq. 10%, 0.8 mL), afforded after preparative HPLC (65% ACN) **12** as an off-white solid (30 mg, 85%). ^**1**^ **H NMR** (600 MHz, DMSO-*d*_6_) δ 7.97 (d, *J* = 1.7 Hz, 2H), 7.84 (s, 1H), 7.73 (d, *J* = 1.4 Hz, 2H), 7.39 (d, *J* = 3.2 Hz, 1H), 7.25 (dd, *J* = 9.0, 3.2 Hz, 1H), 6.93–6.88 (m, 3H), 5.10 (s, 2H). ^**13**^ **C NMR** (126 MHz, DMSO-*d*_6_) δ 171.59, 159.8, 155.61, 150.6, 146.0, 139.2, 136.5, 132.8, 131.6, 129.8, 128.5, 124.0, 118.1, 114.4, 114.1, 112.9, 69.8. **HR-MS** (ESI) calcd for C_24_H_15_O_10_ [*M*–H]^−^: 463.0670, found 463.0676.

#### 5-((3,5-*di*(Thiophen-3-yl)­benzyl)­oxy)-2-hydroxybenzoic Acid (13)

According to **GP-C**, using **7** (80 mg, 0.19 mmol) thiophen-3-ylboronic acid (64 mg, 0.50 mmol), K_2_CO_3_ (61 mg, 0.48 mmol,) and [Pd­(PPh_3_)_4_] (12 mg, 0.01 mmol) in 1,4-dioxane/H_2_O (6:1, 0.2 M) under microwave irradiation at 90 °C for 1 h, afforded after preparative HPLC (75% ACN) **13** as a light brown solid (30 mg, 37%). ^**1**^ **H NMR** (500 MHz, DMSO-*d*_6_) δ 10.86 (br, 1H), 8.0–8.0 (m, 3H), 7.72 (s, 2H), 7.7–7.7 (m, 4H), 7.42 (d, 1H, *J* = 3.1 Hz), 7.28 (d, 1H, *J* = 8.8 Hz), 6.92 (d, 1H, *J* = 9.0 Hz), 5.14 (s, 2H). ^**13**^ **C NMR** (126 MHz, DMSO-*d*_6_) δ 171.5, 155.5, 150.6, 141.1, 138.3, 135.9, 127.1, 126.4, 124.2, 123.3, 121.6, 118.2, 114.0, 112.7, 69.9. **HR-MS** (ESI) calcd for C_22_H_15_O_4_S_2_ [*M*–H]^−^: 407.0417, found 407.0397.

#### 5-((3,5-Bis­(5-(((*tert*-butoxycarbonyl)­amino)­methyl)­thiophen-3-yl)­benzyl)­oxy)-2-hydroxybenzoic Acid (14)

According to **GP-C**, using **7** (80 mg, 0.19 mmol) 5­(((*tert*butoxycarbonyl)­amino)­methyl)­thiophen-3-yl)­boronic acid (127 mg, 0.50 mmol), K_2_CO_3_ (61 mg, 0.47 mmol,) and [Pd­(PPh_3_)_4_] (12 mg, 0.01 mmol) in 1,4-dioxane/H_2_O (6:1, 0.2 M) under microwave irradiation at 90 °C for 1 h, afforded after preparative HPLC (82% ACN) **14** as a pink solid (39 mg, 30%). ^**1**^ **H NMR** (500 MHz, CDCl_3_-*d*) δ 10.43 (br s, 1H), 7.65 (s, 1H), 7.55 (br s, 3H), 7.36 (d, 2H, *J* = 1.0 Hz), 7.16 (dd, 1H, *J* = 3.1, 9.0 Hz), 6.90 (d, 1H, *J* = 9.2 Hz), 5.08 (s, 2H), 5.0–5.1 (m, 1H), 4.51 (br d, 4H, *J* = 5.6 Hz), 1.49 (s, 18H). ^**13**^ **C NMR** (126 MHz, CDCl_3_-*d*) δ 172.1, 156.8, 155.9, 150.8, 142.8, 141.6, 137.8, 136.6, 125.4, 125.1, 124.0, 120.1, 118.6, 114.0, 111.4, 80.3, 70.8, 39.6, 28.4. **HR-MS** (ESI) calcd for C_34_H_37_N_2_O_8_S_2_ [*M*–H]^−^: 665.1996, found 665.2003.

#### 5-((3,5-Bis­(5-(aminomethyl)­thiophen-3-yl)­benzyl)­oxy)-2-hydroxybenzoic Acid (15)

To a stirring solution of **14** (35 mg, 0.525 mmol) in DCM (106 μL, 0.44 M), trifluoroacetic acid (14 μL, mmol) was added at 0 °C. The reaction mixture was stirred at rt for 2 h. After completion of the reaction, the mixture was cooled to 0 °C and quenched with water. The aqueous phase was extracted with DCM (3 x). The combined organic layers were dried over MgSO_4_, filtered, concentrated *in vacuo* followed by purification with preparative HPLC (60% ACN) to afford **15** (10 mg, 41%). ^**1**^ **H NMR** (500 MHz, DMSO-*d*_6_) δ 7.90 (d, *J* = 13.2 Hz, 3H), 7.65 (d, *J* = 17.4 Hz, 4H), 7.41 (d, *J* = 3.2 Hz, 1H), 6.89 (dd, *J* = 8.7, 3.3 Hz, 1H), 6.58 (d, *J* = 8.7 Hz, 1H), 5.07 (d, *J* = 8.1 Hz, 2H), 4.18 (s, 4H). ^**13**^ **C NMR** (DMSO-*d*_6_, 126 MHz) δ 170.8, 156.9, 148.7, 140.6, 139.1, 135.6, 135.4, 129.5, 125.9, 123.9, 122.4, 121.3, 119.5, 116.0, 115.6, 69.8, 38.4. **HR-MS** (ESI) calcd for C_24_H_21_N_2_S_2_O_4_ [*M*–H]^−^: 465.0948, found 465.0943.

#### 5-((3,5-*di*(Pyridin-3-yl)­benzyl)­oxy)-2-hydroxybenzoic Acid (16)

According to **GP-C**, using **7** (50 mg, 0.12 mmol) pyridin-3-ylboronic acid (46 mg, 0.37 mmol), K_2_CO_3_ (103 mg, 0.74 mmol,) and [Pd­(PPh_3_)_4_] (7 mg, 0.006 mmol) in 1,4-dioxane/H_2_O (6:1, 0.2 M) under microwave irradiation at 90 °C for 1 h, afforded after preparative HPLC (55% ACN) **16** as a white solid (10 mg, 20%). ^**1**^ **H NMR** (500 MHz, DMSO-*d*_6_) δ 9.03 (d, *J* = 2.4 Hz, 2H), 8.62 (dd, *J* = 4.7, 1.6 Hz, 2H), 8.23 (dt, *J* = 7.9, 2.0 Hz, 2H), 8.04 (d, *J* = 1.9 Hz, 1H), 7.87 (d, *J* = 1.7 Hz, 2H), 7.53 (dd, *J* = 7.9, 4.8 Hz, 2H), 7.44 (d, *J* = 3.2 Hz, 1H), 7.31–7.25 (m, 1H), 6.93–6.88 (m, 1H), 5.22 (s, 2H). ^**13**^ **C NMR** (DMSO-*d*_6_, 126 MHz) δ 150.8, 149.6, 148.2, 141.1, 140.0, 135.0, 130.3, 129.3, 125.7, 124.7, 123.0, 118.7, 114.5, 69.3, 40.6, 40.4, 40.2, 40.1, 39.9. **HR-MS** (ESI) calcd for C_24_H_17_N_2_O_4_ [*M*–H]^−^: 397.1193, found 397.1192.

#### 5-((3,5-Bis­(6-*iso*propoxypyridin-3-yl)­benzyl)­oxy)-2-hydroxybenzoic Acid (17)

According to **GP-C**, using **7** (80 mg, 0.190 mmol) (6-isopropoxypyridin-3-yl)­boronic acid (109 mg, 0.569 mmol), K_2_CO_3_ (69 mg, 0.497 mmol,) and [Pd­(PPh_3_)_4_] (12 mg, 0.010 mmol) in 1,4-dioxane/H_2_O (6:1, 0.2 M) under microwave irradiation at 90 °C for 1 h, afforded after preparative HPLC (70% ACN) **17** as a white solid (30 mg, 30%). ^**1**^ **H NMR** (500 MHz, DMSO-*d*_6_) δ 8.57 (d, *J* = 2.6 Hz, 2H), 8.10 (dd, *J* = 8.6, 2.6 Hz, 2H), 7.86 (s, 1H), 7.71 (s, 2H), 7.42 (d, *J* = 3.2 Hz, 1H), 7.28 (dd, *J* = 9.0, 3.2 Hz, 1H), 6.91 (d, *J* = 9.0 Hz, 1H), 6.85 (d, *J* = 8.6 Hz, 2H), 5.34–5.27 (m, 2H), 5.18 (s, 2H), 1.32 (d, *J* = 6.2 Hz, 12H). ^**13**^ **C NMR** (126 MHz, DMSO-*d*_6_) δ 171.5, 162.6, 155.6, 150.5, 145.0, 138.7, 138.0, 137.8, 128.5, 124.5, 124.20, 123.8, 118.2, 114.0, 112.8, 111.2, 69.7, 67.7, 21.9. **HR-MS** (ESI) calcd for C_30_H_29_N_2_O_6_ [*M*–H]^−^: 513.2031, found 513.2019.

#### 5-((3,5-Bis­(5-chloro-6-*iso*propoxypyridin-3-yl)­benzyl)­oxy)-2-hydroxybenzoic Acid (18)

According to **GP-C**, using **7** (80 mg, 0.189 mmol) (5-chloro-6-isopropoxypyridin-3-yl)­boronic acid (108 mg, 0.497 mmol), K_2_CO_3_ (61 mg, 0.437 mmol,) and [Pd­(PPh_3_)_4_] (12 mg, 0.010 mmol) in 1,4-dioxane/H_2_O (6:1, 0.2 M) under microwave irradiation at 90 °C for 1 h, afforded after preparative HPLC (90% ACN) **18** as a white solid (46 mg, 40%). ^**1**^ **H NMR** (500 MHz, DMSO-*d*_6_) δ 8.57 (d, *J* = 2.2 Hz, 2H), 8.39 (d, *J* = 2.2 Hz, 2H), 7.98 (s, 1H), 7.80 (d, *J* = 1.3 Hz, 2H), 7.42 (d, *J* = 3.2 Hz, 1H), 7.28 (dd, *J* = 9.0, 3.2 Hz, 1H), 6.91 (d, *J* = 9.0 Hz, 1H), 5.36 (dq, *J* = 12.4, 6.2 Hz, 2H), 5.16 (s, 2H), 1.36 (d, *J* = 6.2 Hz, 12H). ^**13**^ **C NMR** (126 MHz, DMSO-*d*_6_) δ 171.5, 157.5, 155.6, 150.5, 143.1, 138.8, 137.2, 136.7, 129.6, 125.1, 124.2, 118.1, 117.5, 114.0, 112.9, 69.7, 69.4, 21.8. **HR-MS** (ESI) calcd for C_30_H_27_Cl_2_N_2_O_6_ [*M*–H]^−^: 581.1251, found 581.1248.

#### 5-((3-(*tert*-Butyl)­benzyl)­oxy)-2-hydroxybenzoic Acid (19)

According to **GP-B**, using **47** (80 mg, 0.25 mmol) and NaOH (in aq. 10%, 0.5 mL) afforded after preparative HPLC **19** as an off–white solid (68 mg, 0.25 mmol, 90%). ^**1**^ **H NMR** (500 MHz, DMSO-*d*_6_) δ 7.47 (s, 1H), 7.3–7.4 (m, 3H), 7.2–7.3 (m, 2H), 6.90 (d, 1H, *J* = 9.0 Hz), 5.04 (s, 2H), 1.28 (s, 9H). ^**13**^ **C NMR** (126 MHz, DMSO-*d*_6_) δ 171.6, 155.5, 150.7, 150.6, 136.6, 128.1, 125.0, 124.7, 124.7, 124.3, 118.1, 113.9, 112.6, 70.3, 34.4, 31.1. **HR-MS** (ESI) calcd for C_16_H_19_O_4_ [*M*–H]^−^: 299.1288, found 299.1285.

#### 5-((3-(Furan-3-yl)­benzyl)­oxy)-2-hydroxybenzoic Acid (20)

According to **GP-C**, using **50** (80 mg, 0.25 mmol) furan-3-ylboronic acid (42 mg, 0.37 mmol), K_2_CO_3_ (75 mg, 0.55 mmol) and [Pd­(PPh_3_)_4_] (14 mg, 0.01 mmol) in 1,4-dioxane/H_2_O (6:1, 0.2 M) under microwave irradiation at 90 °C for 1 h afforded after preparative HPLC (70% ACN) **20** as a white solid (27 mg, 35%). ^**1**^ **H NMR** (500 MHz, DMSO-*d*_6_) δ 8.20 (s, 1H), 7.75 (s, 1H), 7.69 (s, 1H), 7.57 (d, 1H, *J* = 7.6 Hz), 7.6–7.4 (m, 2H), 7.5–7.3 (m, 1H), 7.25 (dd, 1H, *J* = 3.1, 9.0 Hz), 6.97 (s, 1H), 6.91 (d, 1H, *J* = 9.0 Hz), 5.12 (s, 2H). ^**13**^ **C NMR** (126 MHz, DMSO-*d*_6_) δ 171.5, 155.5, 150.6, 144.4, 139.5, 137.6, 132.1, 128.9, 126.3, 125.6, 125.0, 124.9, 124.2, 118.1, 113.9, 112.7, 108.7, 69.8. **HR-MS** (ESI) calcd for C_18_H_13_O_5_ [*M*–H]^−^: 309.07685, found: 309.07692.

#### 2-Hydroxy-5-((3-(thiophen-3-yl) benzyl)­oxy)­benzoic Acid (21)

According to **GP-C**, using **50** (100 mg, 0.31 mmol) thiophen-3-ylboronic acid (64 mg, 0.5 mmol), K_2_CO_3_ (128 mg, 0.93 mmol,) and [Pd­(PPh_3_)_4_] (18 mg, 0.02 mmol) in 1,4-dioxane/H_2_O (6:1, 0.2 M) under microwave irradiation at 90 °C for 1 h afforded after preparative HPLC (75% ACN) **21** as a white solid (20 mg, 20%). ^**1**^ **H NMR** (500 MHz, DMSO-*d*_6_) δ 7.90 (dd, *J* = 3.1, 1.3 Hz, 1H), 7.80 (s, *J* = 1.8 Hz, 1H), 7.68 (dt, *J* = 7.7, 1.6 Hz, 1H), 7.66 (dd, *J* = 5.0, 2.9 Hz, 1H), 7.57 (dd, *J* = 5.0, 1.4 Hz, 1H), 7.44 (t, *J* = 7.6 Hz, 1H), 7.41–7.35 (m, 2H), 7.26 (dd, *J* = 9.0, 3.2 Hz, 1H), 6.92 (d, *J* = 9.0 Hz, 1H), 5.11 (s, 2H). ^**13**^ **C NMR** (126 MHz, DMSO-*d*_6_) δ 171.6, 155.5, 150.6, 141.2, 137.7, 135.2, 129.0, 127.2, 126.4, 126.2, 125.6, 125.4, 124.2, 121.2, 118.2, 113.9, 112.7, 69.8. **HR-MS** (ESI) calcd for C_18_H_13_O_4_S [*M*–H]^−^: 325.0540, found 325.0539.

#### 5-((3-(1*H*-1,2,3-Triazol-1-yl)­benzyl)­oxy)-2-hydroxybenzoic Acid (22)

According to **GP-F**, using **55** (30 mg, 0.11 mmol) sodium ascorbate (8.3 mg, 0.04 mmol), CuSO_4_(H_2_O)_5_ (5.2 mg, 0.02 mmol) and ethynyltrimethylsilane (22 μL, 0.11 mmol), afforded after preparative HPLC (35% ACN) **22** as a white solid (14.6 mg, 28%). ^**1**^ **HNMR** (500 MHz, DMSO-*d*_6_) δ 8.85 (d, *J* = 1.2 Hz, 1H), 8.02 (st, *J* = 2.1 Hz, 1H), 7.98 (sd, *J* = 1.1 Hz, 1H), 7.90–7.84 (m, 1H), 7.62 (t, *J* = 7.8 Hz, 1H), 7.56 (d, *J* = 7.7 Hz, 1H), 7.39 (d, *J* = 3.2 Hz, 1H), 7.27 (dd, *J* = 9.0, 3.2 Hz, 1H), 6.92 (d, *J* = 9.0 Hz, 1H), 5.19 (s, 2H). ^**13**^ **CNMR** (126 MHz, DMSO-*d*_6_) δ 171.5, 155.7, 150.4, 139.3, 136.8, 134.5, 130.0, 127.6, 124.2, 123.2, 119.5, 119.1, 118.2, 114.0, 112.7, 69.2. **HR-MS** (ESI) calcd for C_16_H_12_N_3_O_4_ [*M*–H]^−^: 310.0833, found 310.0831.

#### 2-Hydroxy-5-((3-(4-((3-(hydroxymethyl)­phenoxy)­methyl)-1*H*-1,2,3-triazol-1-yl)­benzyl)­oxy)­benzoic Acid (23)

According to **GP-F**, using **55** (30 mg, 0.11 mmol), sodium ascorbate (8.3 mg, 0.042 mmol), CuSO_4_(H_2_O)_5_ (5.2 mg, 0.02 mmol) and (4-(prop-2-yn-1-yloxy)­phenyl)­methanol (17.1 mg, 0.11 mmol), afforded after preparative HPLC **23** as a white solid (14 mg, 36%). ^**1**^ **H NMR** (500 MHz, DMSO-*d*_6_) δ 8.97 (s, 1H), 8.03 (st, *J* = 1.9 Hz, 1H), 7.87 (dt, *J* = 8.0, 1.6 Hz, 1H), 7.63 (t, *J* = 7.8 Hz, 1H), 7.57 (d, *J* = 7.7 Hz, 1H), 7.39 (d, *J* = 3.2 Hz, 1H), 7.28–7.24 (m, 2H), 7.02 (s, *J* = 2.0 Hz, 1H), 6.96 – 6.88 (m, 3H), 5.23 (s, 2H), 5.19 (s, 2H), 4.48 (s, 2H). ^**13**^ **C NMR** (126 MHz, DMSO-*d*_6_) δ 171.5, 158.0, 155.7, 150.4, 144.4, 144.1, 139.3, 136.7, 130.1, 129.2, 127.7, 124.2, 122.8, 119.5, 119.1, 119.0, 118.2, 114.0, 112.9, 112.8, 112.7, 69.2, 62.7, 60.9. **HR-MS** (ESI) calcd for C_24_H_20_N_3_O_6_ [*M*–H]^−^: 446.1357, found 446.1353.

#### 5-((3-(4-(3-Carboxypropyl)-1*H*-1,2,3-triazol-1-yl)­benzyl)­oxy)-2-hydroxybenzoic Acid (24)

According to **GP-F**, using **55** (30 mg, 0.11 mmol), sodium ascorbate (8.3 mg, 0.04 mmol), CuSO_4_(H_2_O)_5_ (5.2 mg, 0.02 mmol) and hex-5-ynoic acid (14.3 μL, 0.11 mmol), afforded after preparative HPLC **24** as a white solid (25 mg, 55%). ^**1**^ **H NMR** (500 MHz, DMSO-*d*_6_) δ 8.83 (s, 1H), 8.02 (m, 1H), 7.86 (dt, *J* = 7.9, 1.7 Hz, 1H), 7.61 (t, *J* = 7.8 Hz, 1H), 7.56 (d, *J* = 7.6 Hz, 1H), 7.39 (d, *J* = 3.2 Hz, 1H), 7.27 (dd, *J* = 9.0, 3.2 Hz, 1H), 6.92 (d, *J* = 9.0 Hz, 1H), 5.18 (s, 2H), 4.63 (s, 2H), 3.66–3.61 (m, 2H), 3.59–3.54 (m, 2H), 3.49 (t, *J* = 5.2 Hz, 2H), 3.42 (t, *J* = 5.2 Hz, 2H). ^**13**^ **C NMR** (126 MHz, DMSO-*d*_6_) δ 171.5, 155.6, 150.4, 145.2, 139.3, 136.7, 130.0, 127.6, 124.2, 122.2, 119.4, 119.0, 118.2, 114.0, 112.8, 72.4, 69.7, 69.2, 69.1, 63.4, 60.2. **HR-MS** (ESI) calcd for C_20_H_18_N_3_O_6_ [*M*–H]^−^: 396.1201, found 396.1197.

#### 5-((3-(4-(*tert*-Butyl)-1*H*-1,2,3-triazol-1-yl)­benzyl)­oxy)-2-hydroxybenzoic Aacid (25)

According to **GP-F**, using **55** (30 mg, 0.105 mmol), sodium ascorbate (8.3 mg, 0.04 mmol), CuSO_4_(H_2_O)_5_ (5.2 mg, 0.02 mmol) and 3,3-dimethylbut-1-yne (8.6 mg, 0.11 mmol), afforded after preparative HPLC **25** as a white solid (15 mg, 37%). ^**1**^ **HNMR** (500 MHz, DMSO-*d*_6_) δ 8.61 (s, 1H), 8.00 (st, *J* = 1.9 Hz, 1H), 7.87–7.83 (m, 1H), 7.59 (t, *J* = 7.8 Hz, 1H), 7.53 (dt, *J* = 7.7, 1.4 Hz, 1H), 7.39 (d, *J* = 3.2 Hz, 1H), 7.27 (dd, *J* = 9.0, 3.2 Hz, 1H), 6.92 (d, *J* = 9.0 Hz, 1H), 5.17 (s, 2H), 1.35 (s, 9H). ^**13**^ **C NMR** (124 MHz, DMSO-*d*_6_) δ 171.5, 157.5, 155.7, 150.4, 139.2, 137.0, 130.0, 127.2, 124.2, 119.1, 118.8, 118.3, 118.2, 114.0, 112.7, 69.3, 30.6, 30.2 (3C). **HR-MS** (ESI) calcd for C_20_H_20_N_3_O_4_ [*M*–H]^−^: 366.1459, found 366.1455.

#### 5-((3-(4-Cyclopentyl-1*H*-1,2,3-triazol-1-yl)­benzyl)­oxy)-2-hydroxybenzoic Acid (26)

According to **GP-F**, using **55** (30 mg, 0.11 mmol), sodium ascorbate (8.3 mg, 0.04 mmol), CuSO_4_(H_2_O)_5_ (5.2 mg, 0.02 mmol) and ethynylcyclopentane (17.1 mg, 0.11 mmol), afforded after preparative HPLC **26** as a white solid (14 mg, 35%). ^**1**^ **HNMR** (500 MHz, DMSO-*d*_6_) δ 8.61 (s, 1H), 7.99 (st, *J* = 1.9 Hz, 1H), 7.83 (dd, *J* = 7.1, 2.1 Hz, 1H), 7.59 (t, *J* = 7.8 Hz, 1H), 7.53 (d, *J* = 7.6 Hz, 1H), 7.39 (d, *J* = 3.2 Hz, 1H), 7.27 (dd, *J* = 9.0, 3.2 Hz, 1H), 6.92 (d, *J* = 9.0 Hz, 1H), 5.17 (s, 2H), 3.19 (m, 1H), 2.04 (ddt, *J* = 11.1, 8.9, 2.1 Hz, 2H), 1.78–1.61 (m, 6H). ^**13**^ **CNMR** (126 MHz, DMSO-*d*_6_) δ 171.5, 155.7, 152.6, 150.4, 139.2, 136.9, 130.0, 127.2, 124.2, 119.2, 119.1, 118.7, 118.2, 114.0, 112.7, 69.3, 36.2, 32.7 (2C), 24.7 (2C). **HR-MS** (ESI) *m*/*z* calcd for C_21_H_20_N_3_O_4_ [*M*–H]^−^: 378.1459, found 378.1457.

#### 2-Hydroxy-5-((3-(pyridin-3-yl)­benzyl)­oxy)­benzoic Acid (27)

According to **GP-C**, using **50** (80 mg, 0.25 mmol), pyridin-3-ylboronic acid (46 mg, 0.37 mmol), K_2_CO_3_ (75 mg, 0.54 mmol,) and [Pd­(PPh_3_)_4_] (14 mg, 0.01 mmol) afforded after preparative HPLC (60% ACN) **27** as a white solid (32 mg, 40%). ^**1**^ **H NMR** (500 MHz, DMSO-*d*_6_) δ 8.91 (br s, 1H), 8.59 (br s, 1H), 8.09 (br d, 1H, *J* = 7.9 Hz), 7.81 (s, 1H), 7.70 (d, 1H, *J* = 7.2 Hz), 7.5–7.6 (m, 3H), 7.39 (d, 1H, *J* = 3.1 Hz), 7.26 (dd, 1H, *J* = 3.1, 9.0 Hz), 6.91 (d, 1H, *J* = 9.0 Hz), 5.15 (s, 2H). ^**13**^ **C NMR** (126 MHz, DMSO-*d*_6_) δ 171.5, 150.5, 148.6, 147.6, 138.1, 137.2, 135.4, 134.2, 129.3, 127.4, 126.4, 126.2, 124.2, 118.1, 114.0, 112.8, 69.7. **HR-MS** (ESI) calcd for C_19_H_14_NO_4_ [*M*–H]^−^: 320.0928, found: 320.0908.

#### 2-Hydroxy-5-((3-(6-*iso*propoxypyridin-3-yl)­benzyl)­oxy)­benzoic Acid (28)

According to **GP-C**, using **50** (80 mg, 0.25 mmol), (6-isopropoxypyridin-3-yl)­boronic acid (90 mg, 0.49 mmol), K_2_CO_3_ (103 mg, 0.75 mmol,) and [Pd­(PPh_3_)_4_] (14 mg, 0.01 mmol) afforded after preparative HPLC (65% ACN) **28** as a white solid (41 mg, 44%). ^**1**^ **H NMR** (500 MHz, DMSO-*d*_6_) δ 8.46 (d, *J* = 2.6 Hz, 1H), 7.98 (dd, *J* = 8.6, 2.6 Hz, 1H), 7.72 (s, 1H), 7.61 (d, *J* = 7.6 Hz, 1H), 7.50–7.41 (m, 2H), 7.38 (d, *J* = 3.2 Hz, 1H), 7.25 (dd, *J* = 9.0, 3.2 Hz, 1H), 6.91 (d, *J* = 9.0 Hz, 1H), 6.83 (d, *J* = 8.6 Hz, 1H), 5.29 (hept, *J* = 6.1 Hz, 1H), 5.12 (s, 2H), 1.31 (d, *J* = 6.2 Hz, 6H). ^**13**^ **C NMR** (126 MHz, DMSO-*d*_6_) δ 171.6, 162.5, 155.6, 150.6, 144.7, 137.9, 137.6, 137.2, 129.2, 128.6, 126.6, 125.8, 125.6, 124.2, 118.2, 114.0, 112.8, 111.3, 69.79, 67.7, 21.9. **HR-MS** (ESI) calcd for C_22_H_20_NO_5_ [*M*–H]^−^: 378.1347, found: 378.1340.

#### 2-Hydroxy-5-((3-(quinolin-4-yl)­benzyl)­oxy)­benzoic Acid (29)

According to **GP-C**, using **50** (80 mg, 0.25 mmol) quinolin-4-ylboronic acid (86 mg, 0.5 mmol), K_2_CO_3_ (103 mg, 0.75 mmol,) and [Pd­(PPh_3_)_4_] (14 mg, 0.012 mmol) afforded after preparative HPLC (70% ACN) **29** as a white solid (44 mg, 48%). ^**1**^ **H NMR** (500 MHz, DMSO-*d*_6_) δ 9.36 (s, 1H), 8.48 (s, 1H), 8.24 (d, *J* = 9.0 Hz, 1H), 7.86–7.69 (m, 3H), 7.66–7.46 (m, 4H), 7.41 (s, 1H), 7.24 (d, *J* = 5.3 Hz, 1H), 6.90 (d, *J* = 8.3 Hz, 1H), 5.22 (s, 2H). ^**13**^ **C NMR** (126 MHz, DMSO-*d*_6_) δ 171.5, 155.7, 152.1, 150.3, 142.4, 137.7, 136. 5, 133.1, 132.3, 131.2, 129.3, 129.0, 129.0, 128.2, 128.0, 127.6, 127.3, 124.0, 118.0, 114.2, 113.4, 69.7. **HR-MS** (ESI) calcd for C_23_H_18_NO_4_ [*M*+H]^+^: 372.1230, found: 372.1218.

#### 5-((3-(5-Chloro-6-isopropoxypyridin-3-yl)-5-(4-(1-hydroxycyclopentyl)-1*H*-1,2,3-triazol-1-yl)­benzyl)­oxy)-2-hydroxybenzoic Acid (30)

According to **GP-G**, using **62** (80 mg, 0.18 mmol), 1-ethynylcyclopentan-1-ol (19 mg, 0.18 mmol), sodium ascorbate (14 mg, 0.07 mmol) and copper­(II) sulfate pentahydrate (9 mg, 0.03 mmol), afforded after preparative HPLC (75% ACN) **30** as a white solid (27 mg, 27%). ^**1**^ **H NMR** (500 MHz, DMSO-*d*_6_) δ 8.82 (s, 1H), 8.61 (d, 1H, *J* = 2.3 Hz), 8.42 (d, 1H, *J* = 2.3 Hz), 8.17 (s, 1H), 8.06 (s, 1H), 7.91 (s, 1H), 7.42 (d, 1H, *J* = 3.2 Hz), 7.30 (dd, 1H, *J* = 3.2, 9.0 Hz), 6.92 (d, 1H, *J* = 9.0 Hz), 5.38 (m, 1H, *J* = 6.2 Hz), 5.23 (s, 2H), 5.17 (s, 1H), 2.0–2.1 (m, 2H), 1.8–2.0 (m, 4H), 1.7–1.8 (m, 2H), 1.37 (d, 6H, *J* = 6.3 Hz). ^**13**^ **C NMR** (126 MHz, DMSO-*d*_6_) δ 171.5, 157.8, 155.7, 155.7, 150.4, 143.3, 140.0, 137.6, 137.5, 137.2, 128.7, 125.1, 124.2, 119.8, 118.2, 117.9, 117.6, 116.9, 114.1, 112.9, 77.4, 69.6, 69.2, 40.7, 23.3, 21.8. **HR-MS** (ESI) calcd for C_29_H_28_ClN_4_O_6_ [*M*–H]^−^: 563.1702, found: 563.1701.

#### 5-((3-(5-Chloro-6-isopropoxypyridin-3-yl)-5-(4-cyclopentyl-1*H*-1,2,3-triazol-1-yl)­benzyl)­oxy)-2-hydroxybenzoic Acid (31)

According to **GP-G**, using **62**(80 mg, 0.18 mmol), ethynylcyclopentane (20 μL, 0.18 mmol), sodium ascorbate (14 mg, 0.07 mmol) and copper­(II) sulfate pentahydrate (9 mg, 0.03 mmol), afforded after preparative HPLC (85% ACN) **31** as a white solid (55 mg, 57%). ^**1**^ **H NMR** (500 MHz, DMSO-*d*_6_) δ 8.76 (s, 1H), 8.60 (d, 1H, *J* = 2.1 Hz), 8.38 (d, 1H, *J* = 2.3 Hz), 8.13 (s, 1H), 8.04 (s, 1H), 7.90 (s, 1H), 7.42 (d, 1H, *J* = 3.2 Hz), 7.30 (dd, 1H, *J* = 3.1, 9.0 Hz), 6.93 (d, 1H, *J* = 9.0 Hz), 5.38 (m, 1H, *J* = 1.0 Hz), 5.22 (s, 2H), 3.22 (m, 1H), 2.0–2.1 (m, 2H), 1.6–1.8 (m, 6H), 1.37 (d, 6H, *J* = 6.1 Hz)^.**13**^ **C NMR** (126 MHz, DMSO-*d*_6_) δ 171.5, 157.8, 155.7, 152.6, 150.4, 143.2, 140.0, 137.6, 137.4, 137.1, 128.7, 125.1, 124.2, 119.4, 118.2, 117.8, 117.6, 116.8, 114.1, 112.8, 69.6, 69.2, 38.9, 36.1, 32.7, 24.7, 21.8. **HR-MS** (ESI) calcd for C_29_H_28_ClN_4_O_5_ [*M*–H]^−^: 547.1753, found: 547.1749.

#### 5-((3-(5-Chloro-6-isopropoxypyridin-3-yl)-5-(4-cyclopropyl-1*H*-1,2,3-triazol-1-yl)­benzyl)­oxy)-2-hydroxybenzoic Acid (32)

According to **GP-G**, using **62** (65 mg, 0.14 mmol), ethynylcyclopropane (12 μL, 0.14 mmol), sodium ascorbate (11 mg, 0.05 mmol) and copper­(II) sulfate pentahydrate (7 mg, 0.03 mmol). As LC-MS analysis did not show any progress, 2 eq. of ethynyl cyclopropane (24 μL, 0.28 mmol), 0.2 eq of sodium ascorbate (11 mg, 0.05 mmol) and 0.4 eq. of copper­(II) sulfate pentahydrate (7 mg, 0.03 mmol) were added and the reaction mixture was stirred overnight. The crude material was purified by preparative HPLC (78% ACN) and **33** was isolated as a white solid (22 mg, 30%). ^**1**^ **H NMR** (500 MHz, DMSO-*d*_6_) δ 8.70 (s, 1H), 8.59 (d, 1H, *J* = 2.1 Hz), 8.37 (d, 1H, *J* = 2.1 Hz), 8.10 (s, 1H), 8.00 (s, 1H), 7.90 (s, 1H), 7.42 (d, 1H, *J* = 3.2 Hz), 7.30 (dd, 1H, *J* = 3.2, 9.0 Hz), 6.93 (d, 1H, *J* = 9.0 Hz), 5.38 (m, 1H), 5.22 (s, 2H), 2.0–2.1 (m, 1H), 1.37 (d, 6H, *J* = 6.3 Hz), 1.0–1.2 (m, 2H), 0.8–1.0 (m, 2H). ^**13**^ **C NMR** (126 MHz, DMSO-*d*_6_) δ 171.5, 157.8, 155.7, 150.4, 150.4, 143.2, 140.0, 137.5, 137.4, 137.1, 128.7, 125.2, 124.2, 119.1, 118.2, 117.8, 117.6, 116.8, 114.1, 112.7, 69.6, 69.2, 21.8, 7.8, 6.5. **HR-MS** (ESI) calcd for C_27_H_26_ClN_4_O_5_ [*M*+H]^+^: 521.1586, found 521.1582.

#### 5-((3-(5-Chloro-6-isopropoxypyridin-3-yl)-5-(4-(thiophen-3-yl)-1*H*-1,2,3-triazol-1-yl)­benzyl)­oxy)-2-hydroxybenzoic Acid (33)

According to **GP-G**, using **62** (65 mg, 0.14 mmol), 3-ethynylthiophene (14 μL, 0.14 mmol), sodium ascorbate (11 mg, 0.05 mmol) and copper­(II) sulfate pentahydrate (7 mg, 0.03 mmol) reacted for 3 h. The crude material was purified by preparative HPLC (80% ACN) and **33** was isolated as a white solid (20 mg, 25%). ^**1**^ **H NMR** (500 MHz, DMSO-*d*_6_) δ 9.31 (s, 1H), 8.61 (d, 1H, *J* = 2.3 Hz), 8.40 (d, 1H, *J* = 2.3 Hz), 8.19 (s, 1H), 8.09 (s, 1H), 7.9–8.0 (m, 2H), 7.72 (dd, 1H, *J* = 3.0, 5.0 Hz), 7.59 (dd, 1H, *J* = 1.2, 5.0 Hz), 7.44 (d, 1H, *J* = 3.2 Hz), 7.31 (dd, 1H, *J* = 3.1, 9.0 Hz), 6.93 (d, 1H, *J* = 9.0 Hz), 5.39 (m, 1H), 5.25 (s, 2H), 1.37 (d, 6H, *J* = 6.3 Hz). ^**13**^ **C NMR** (126 MHz, DMSO-*d*_6_) δ 171.5, 157.8, 155.7, 150.4, 143.9, 143.2, 140.1, 137.5, 137.4, 137.1, 131.4, 128.6, 127.5, 125.7, 125.5, 124.2, 121.5, 119.5, 118.2, 118.0, 117.6, 117.1, 114.1, 112.8, 69.6, 69.2, 21.8. **HR-MS** (ESI) calcd for C_28_H_22_ClN_4_O_5_S [*M*–H]^−^: 561.1004, found: 561.1001.

#### 5-((3-(5-Chloro-6-isopropoxypyridin-3-yl)-5-(4-(thiophen-2-yl)-1*H*-1,2,3-triazol-1-yl)­benzyl)­oxy)-2-hydroxybenzoic Acid (34)

According to **GP-F**, using **62** (65 mg, 0.14 mmol), 2 ethynylthiophene (14 μL, 0.14 mmol), sodium ascorbate (11 mg, 0.05 mmol) and copper­(II) sulfate pentahydrate (7 mg, 0.03 mmol) reacted for 3 h. The crude material was purified by preparative HPLC (80% ACN) and **34** has been isolated as a white solid (21 mg, 26%). ^**1**^ **H NMR** (DMSO-*d*_6_, 500 MHz) δ 9.36 (s, 1H), 8.62 (d, 1H, *J* = 2.1 Hz), 8.40 (d, 1H, *J* = 2.1 Hz), 8.21 (s, 1H), 8.10 (s, 1H), 7.96 (s, 1H), 7.62 (d, 1H, *J* = 5.0 Hz), 7.54 (d, 1H, *J* = 3.5 Hz), 7.44 (d, 1H, *J* = 3.1 Hz), 7.31 (dd, 1H, *J* = 3.1, 9.0 Hz), 7.20 (dd, 1H, *J* = 3.8, 4.7 Hz), 6.93 (d, 1H, *J* = 9.0 Hz), 5.39 (m, 1H), 5.25 (s, 2H), 1.37 (d, 6H, *J* = 6.3 Hz). ^**13**^ **C NMR** (DMSO-*d*_6_, 126 MHz) δ 171.5, 157.8, 155.7, 150.3, 143.3, 142.7, 140.2, 137.5, 137.2, 137.1, 132.3, 128.6, 128.1, 126.0, 125.6, 124.6, 124.1, 119.1, 118.2, 118.0, 117.6, 117.1, 114.1, 112.9, 69.6, 69.2, 21.8. **HR-MS** (ESI) calcd for C_28_H_22_ClN_4_O_5_S [*M*–H]^−^: 561.1004, found: 561.0976.

#### 5-((3-(5-Chloro-6-isopropoxypyridin-3-yl)-5-(4-((*di*methylamino)­methyl)-1*H*-1,2,3-triazol-1-yl)­benzyl)­oxy)-2-hydroxybenzoic Acid (35)

According to **GP-G**, using **62** (80 mg, 0.18 mmol), *N*,*N*-dimethylprop-2-yn-1-amine (19 μL, 0.18 mmol), sodium ascorbate (14 mg, 0.07 mmol) and copper­(II) sulfate pentahydrate (9 mg, 0.03 mmol), afforded after preparative HPLC (60% ACN) **35** as a white solid (37 mg, 39%). ^**1**^ **H NMR** (500 MHz, DMSO-*d*_6_) δ 9.04 (s, 1H), 8.57 (d, 1H, *J* = 2.1 Hz), 8.37 (d, 1H, *J* = 2.1 Hz), 8.15 (s, 1H), 8.00 (s, 1H), 7.90 (s, 1H), 7.39 (d, 1H, *J* = 3.2 Hz), 6.97 (dd, 1H, *J* = 3.2, 8.7 Hz), 6.64 (d, 1H, *J* = 8.7 Hz), 5.37 (m, 1H), 5.15 (s, 2H), 4.24 (s, 2H), 2.66 (s, 6H), 1.36 (d, 6H, *J* = 6.3 Hz). ^**13**^ **C NMR** (126 MHz, DMSO-*d*_6_) δ 171.2, 163.1, 157.8, 156.7, 149.0, 143.2, 140.8, 137.4, 137.2, 137.1, 128.6, 125.6, 124.4, 120.4, 118.1, 117.6, 117.2, 116.6, 114.7, 69.6, 69.0, 51.5, 42.7, 21.8. **HR-MS** (ESI) calcd for C_27_H_27_ClN_5_O_5_ [*M*–H]^−^: 536.1706, found: 536.1704.

#### 5-((3-(4-(*Tert*-butyl)-1*H*-1,2,3-triazol-1-yl)-5-(5-chloro-6-isopropoxypyridin-3-yl)­benzyl)­oxy)-2-hydroxybenzoic Acid (36)

According to **GP-G**, using **62** (80 mg, 0.18 mmol), 3,3dimethylbut-1-yne (22 μL, 0.18 mmol), sodium ascorbate (14 mg, 0.07 mmol) and copper­(II) sulfate pentahydrate (9 mg, 0.03 mmol), afforded after preparative HPLC (78% ACN) **36** as a white solid (61 mg, 65%). ^**1**^ **H NMR** (500 MHz, DMSO-*d*_6_) δ 8.76 (s, 1H), 8.61 (d, 1H, *J* = 2.1 Hz), 8.38 (d, 1H, *J* = 2.1 Hz), 8.14 (s, 1H), 8.04 (s, 1H), 7.90 (s, 1H), 7.42 (d, 1H, *J* = 3.2 Hz), 7.30 (dd, 1H, *J* = 3.1, 9.0 Hz), 6.93 (d, 1H, *J* = 9.0 Hz), 5.38 (m, 1H), 5.23 (s, 2H), 1.3–1.4 (m, 15H). ^**13**^ **C NMR** (126 MHz, DMSO-*d*_6_) δ 171.5, 157.8, 157.5, 155.7, 150.4, 143.3, 140.0, 137.6, 137.4, 137.1, 128.7, 125.1, 124.2, 118.5, 118.2, 117.9, 117.6, 116.8, 114.1, 112.8, 69.6, 69.2, 30.6, 30.2, 21.8. **HR-MS** (ESI) calcd for C_28_H_28_ClN_4_O_5_ [*M*–H]^−^: 535.1753, found: 535.1748.

## Supplementary Material





## Notes

Experimental procedures and compound characterization data (NMR spectra and HPLC purity), molecular docking procedure, biological data and protocols (PDF)
Molecular formula strings (CSV)

## Abbreviations

AMR: antimicrobial resistance
CC_50_: concentration causing 50% cytotoxicity
CFU: colony-forming units
CO_2_: carbon dioxide
CuAAC: copper-catalyzed azide–alkyne cycloaddition
ECF: energy-coupling factor
ECF-FolT2: folate-specific energy-coupling factor transporter
ECF-PanT: pantothenate-specific energy-coupling factor transporter
FA: formic acid
GP-A: general procedure A
GP-B: general procedure B
GP-C: general procedure C
GP-D: general procedure D
GP-E: general procedure E
GP-F: general procedure F
GP-G: general procedure G
LOD: limit of detection
NaOH: sodium hydroxide
PRSP: penicillin-resistant *Streptococcus pneumoniae*
SBVS: structure-based virtual screening
SI: selectivity index
tdDCC: target-directed dynamic combinatorial chemistry
TKC: time-kill curve
(aq.): Aqueous
(calcd): calculated
(equiv) hours (h): equivalents
HRMS: (high -resolution mass spectrometry)
(min): minutes
(rt): room temperature
on: (overnight)
(sat.): saturated
(ppm): part(s) per million
