# Supplementary material for: Hit-to-Lead Optimization of Energy-Coupling Factor (ECF) Transporter Inhibitors as Novel Antibiotic
Source: J Med Chem. 2026 Jun 6;69(12):14069–84. doi: 10.1021/acs.jmedchem.5c02721 (PMC13312448; doi:10.1021/acs.jmedchem.5c02721)

Supporting Information

Hit-to-Lead optimization of energy-coupling factor (ECF) transporters inhibitors as novel antibiotic

Ioulia Exapicheidou,[1,2] Aleksei Tsarenko,[4] Lena Zeller,[1,2] Hamza Ibrahim,[1,5] Carole Baumann,[1,3] Atanaz Shams,[1,2] Patrick A. Hoffmann, [1,2] Yue Li,[5] Andreas M. Kany,[1,3] Jennifer Hermann,[1,3] Dirk J. Slotboom,[4] Rolf Müller,[1,2,3]Andrea Volkamer,[1,3,5] Mostafa M. Hamed,[1,3] Eleonora Diamanti,[1,3] Anna K. H. Hirsch[*1,2,3]

[1]Helmholtz Institute for Pharmaceutical Research (HIPS) -Helmholtz Centre for Infection Research (HZI), Campus Building E 8.1, D-66123, Saarbrücken, Saarland, Germany.

[2] Saarland University, Department of Pharmacy, Campus E8.1, 66123, Saarbrücken, Germany.

[3]Saarland University, PharmaScienceHub (PSH), Campus E2.1, 66123, Saarbrücken, Germany.

[4]Groningen Biomolecular Sciences and Biotechnology Institute, University of Groningen, Nijenborgh 4, 9747AG Groningen, The Netherlands.

[5]Data Driven Drug Design, Center for Bioinformatics, Saarland Informatics Campus, Saarland University, 66123 Saarbrücken, Germany.

*Corresponding author
Prof. A. K. H. Hirsch
Helmholtz Institute for Pharmaceutical Research Saarland (HIPS)-Helmholtz Centre for Infection Research (HZI)
Department of Drug Design and Optimization
Campus Building E8.1, 66123 Saarbrüucken, Saarland, Germany
e-mail: anna.hirsch@helmholtz-hips.de

Contents

1.0 Synthesis and characterization of intermediates 42–45, 47, 49, 50, 52–55, 57–62. 1
2.0 Molecular docking 5
2.1 Protein Preparation 5
2.2 Docking details 5
3.0 Biological evaluation of synthesized compounds 6
3.1 Proteoliposome uptake assay 6
3.2 Whole-cell bacterial uptake assay (ECF-T assay) 6
3.3 Cytotoxicity assays 7
3.4 Antibacterial assays 7
3.5 Time-kill kinetics 8
4.0 *In vitro* ADME assays 9
4.1 Kinetic Turbidimetric Solubility 9
4.2 Lipophilicity Determination 9
4.3 Metabolic Stability in Liver S9 Fractions 9
4.4 Metabolic Stability in Liver Microsomes 10
4.5 Stability in Plasma 10
4.6 hERG Potassium Channel Assay 11
5.0 *In vivo* data 11
5.1 *Galleria mellonella* infection model 11
5.2 *Danio rerio* (zebrafish) infection models 12
5.2.1 Zebrafish husbandry and embryo handling 12
5.2.2 Zebrafish infection model 13
References 13
NMR spectra 15
Compound **30** 15
Compound **31** 16
Compound **32** 17
Compound **33** 18
Compound **36** 19
HPLC-MS purity 20
Compound **18** 20
Compound **30** 20
Compound **31** 21
Compound **32** 21

Compound **33** 22
Compound **36** 22

1.0 Synthesis and characterization of intermediates 42–45, 47, 49, 50, 52–55, 57–62.

Methyl 5-((3,5-dichlorobenzyl)oxy)-2-hydroxybenzoate (42)

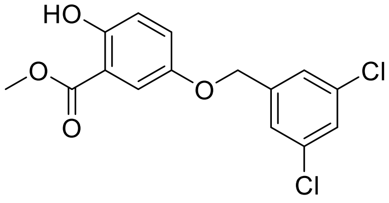

According to **GP-A**, **38** (670 mg, 2.60 mmol) was added to a solution of **37** (400 mg, 2.40 mmol) and K_2CO_3 (655 mg, 4.70 mmol) in acetone (17 mL) to afford after flash chromatography (c-Hex/EtOAc 95:5) **42** as a light brown solid (560 mg, 2.6 mmol, 64%). **^{1}H NMR** (500 MHz, DMSO-*d$_6$)* δ 10.12 (s, 1H), 7.58 (s, 1H), 7.52 (s, 2H), 7.35 (s, 1H), 7.26 (d, 1H, *J*=8.8 Hz), 6.96 (d, 1H, *J*=9.0 Hz), 5.10 (s, 2H), 3.9 (s, 3H). **^{13}C NMR** (126 MHz, DMSO-*d$_6$*) δ 169.3, 155.1, 150.8, 141.9, 134.6, 127.9, 126.6, 124.6, 119.1, 114.2, 113.3, 68.7, 53.0. **LC-MS** calcd for $C_{15}H_{13}Cl_2O_4$ [*M*+H]$^+$:327.0, found 326.91.

Methyl 5-((3-bromo-5-chlorobenzyl)oxy)-2-hydroxybenzoate (43)

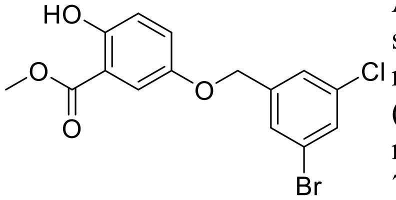

According to **GP-A**, **39** (670 mg, 2.60 mmol) was added to a solution of **37** (400 mg, 2.40 mmol) and K_2CO_3 (655 mg, 4.70 mmol) in acetone (17 mL) to afford after flash chromatography (c-Hex/EtOAc 95:5) **43** as a light brown solid (560 mg, 2.6 mmol, 65%). **^{1}H NMR** (500 MHz, DMSO-*d$_6$*) δ 10.12 (s, 1H), 7.69 (t, *J*=1.7 Hz, 1H), 7.64 (s, 1H), 7.55 (s, 1H), 7.34 (d, *J*= 3.2 Hz, 1H), 7.26 (dd, *J*=9.0, 3.2 Hz, 1H), 6.97 – 6.94 (m, 1H), 5.08 (s, 2H), 3.89 (s, 3H). **^{13}C NMR** (126 MHz, DMSO-*d$_6$*) δ 168.8, 154.6, 150.3, 141.6, 134.2, 130.1, 128.9, 126.5, 124.1, 122.31, 118.66, 113.81, 112.86, 68.19, 52.55. **LC-MS** calcd for $C_{15}H_{13}BrClO_4$ [*M*+H]$^+$: 372.996, found 373.0.

Methyl 5-((3,5-*di*bromobenzyl)oxy)-2-hydroxybenzoate (44)

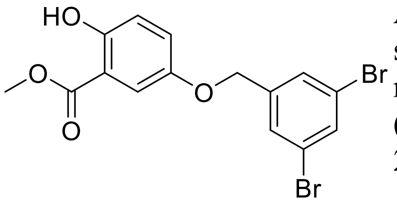

According to **GP-A**, **40** (670 mg, 2.60 mmol) was added to a solution of **37** (400 mg, 2.40 mmol) and K_2CO_3 (655 mg, 4.70 mmol) in acetone (17 mL) to afford after flash chromatography (c-Hex/EtOAc 92:8) **44** as a light brown solid (560 mg, 2.6mmol, 63%). **^{1}H NMR** (500 MHz, DMSO-*d$_6$)* δ 10.11 (s, 1H), 7.80 (t, *J*=1.7 Hz, 1H), 7.68 (d, *J*=1.6 Hz, 2H), 7.34 (d, *J* =3.2 Hz, 1H), 7.26 (dd, *J*=9.0, 3.2 Hz, 1H), 6.95 (d, *J*=9.0 Hz, 1H), 5.08 (s, 2H), 3.89 (s, 3H). **^{13}C NMR** (126 MHz, DMSO-*d$_6$)* δ 168.9, 154.7, 150.3, 141.9, 132.7, 129.4, 124.2, 122.5, 118.6, 113.8, 112.9, 68.1, 52.6. **LC-MS** calcd for $C_{15}H_{11}Br_2O_4$ [*M*–H]$^-$: 414.9, found 414.9.

Methyl 5-((3,5-bis(trifluoromethyl)benzyl)oxy)-2-hydroxybenzoate (45)

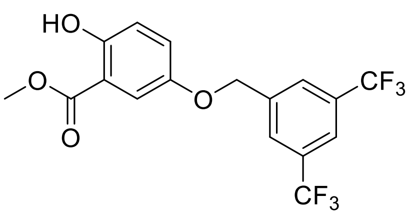

According to **GP-A**, **41** (670 mg, 2.60 mmol) was added to a solution of **37** (400 mg, 2.40 mmol) and K_2CO_3 (655 mg, 4.70 mmol) in acetone (17 mL) to afford after flash chromatography (c-Hex/EtOAc 95:5) **45** as a yellow oil (560 mg, 2.6 mmol, 66%). **^{1}H NMR** (500 MHz, DMSO-*d$_6$)* δ 10.13 (s, 1H), 8.18 (s, 2H), 8.10 (s, 1H), 7.40 (s, 1H), 7.31 (d, 1H, *J*=8.9 Hz), 6.97 (d, 1H, *J*=9.0 Hz), 5.28 (s, 2H), 3.9 (s, 3H). **^{13}C NMR** (126 MHz, DMSO-*d$_6$*) δ 169.3, 155.2,

150.7, 141.1, 131.2, 130.9, 130.7, 130.4, 128.7, 124.7, 122.1, 122.1, 123.8, 119.1, 114.3, 113.3, 68.8, 53.0. **^{19}F NMR** (470 MHz, DMSO-d_6) δ -61.31. **LC-MS** calcd for $C_{17}H_{11}F_6O_4$ [M–H]$^-$: 393.05, found 392.9.

Methyl 5-((3-(*tert*-butyl)benzyl)oxy)-2-hydroxybenzoate (47)

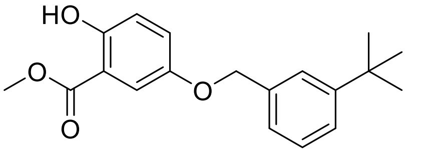

According to **GP-A**, **46** (101 mg, 0.45 mmol) was added to a solution of **37** (75 mg, 0.45 mmol) and K_2CO_3 (123 mg, 0.89 mmol) in acetone (2.8 mL) to give after flash chromatography (c-Hex/EtOAc 95:5) **47** as a light brown solid (92 mg, 0.45 mmol, 70%). **^{1}H NMR** (500 MHz, DMSO-d_6) δ 10.10 (s, 1H), 7.47 (s, 1H), 7.3-7.4 (m, 3H), 7.24 (d, 2H, J=7.5 Hz), 6.93 (d, 1H, J=9.2 Hz), 5.04 (s, 2H), 3.88 (s, 3H), 1.28 (s, 9H). **^{13}C NMR** (126 MHz, DMSO-d_6,) δ 169.0, 154.4, 150.8, 150.8, 136.5, 128.2, 125.0, 124.7, 124.7, 124.3, 118.5, 113.6, 112.6, 70.3, 52.5, 34.4, 31.1. LC-MS (ESI) calcd for $C_{19}H_{23}O_4$ [M+H]$^+$: 315.16, found: 315.92.

Methyl 5-((3-Bromobenzyl)oxy)-2-hydroxybenzoate (49)

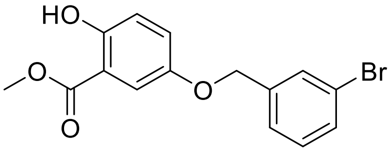

According to **GP-A**, **48** (670 mg, 2.6 mmol) was added to a solution of **37** (400 mg, 2.4 mmol) and K_2CO_3 (655 mg, 4.70 mmol) in acetone (4 mL) to give after flash chromatography (c-Hex/EtOAc 93:7) **49** as a light brown solid (521 mg, 2.4 mmol, 65%). **^{1}H NMR** (500 MHz, DMSO-d_6) δ 10.11 (s, 1H), 7.65 (s, 1H), 7.53 (d, J=7.9 Hz, 1H), 7.45 (d, J=7.6 Hz, 1H), 7.35 (dd, J=13.5, 5.5 Hz, 2H), 7.25 (dd, J=9.0, 3.1 Hz, 1H), 6.95 (d, J=9.0 Hz, 1H), 5.08 (s, 2H), 3.89 (s, 3H). **^{13}C NMR** (126 MHz, DMSO-d_6) δ 168.8, 154.5, 150.5, 139.8, 130.7, 130.2, 126.6, 124.1, 121.7, 118.6, 113.7, 112.8, 68.9, 52.5. LC–MS for $C_{14}H_{12}BrO_4$ [M+H]$^+$: found: 323.1.

5-((3-Bromobenzyl)oxy)-2-hydroxybenzoic acid (50)

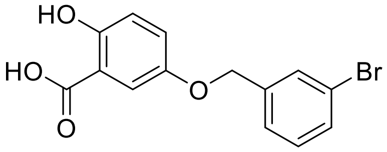

According to **GP-B**, using **49** (520 mg, 1.54 mmol) and NaOH (in aq. 10%, 0.3 mL) to give after flash chromatography (c-Hex/EtOAc 88:12) **50** as an off–white solid (458 mg, 1.54 mmol, 92%) **^{1}H NMR** (500 MHz, DMSO-d_6) δ 10.97 (s, 1H), 7.65 (s, 1H), 7.52 (d, J=8.0 Hz, 1H), 7.45 (d, J=7.7 Hz, 1H), 7.36 (d, J=7.8 Hz, 2H), 7.34 (d, J=3.1 Hz, 2H), 7.22 (dd, J=9.2, 2.3 Hz, 1H), 6.90 (d, J=9.0 Hz, 1H), 5.07 (s, 2H). **^{13}C NMR** (126 MHz, DMSO-d_6) δ 171.7, 155.8, 150.4, 140.1, 130.9, 130.8, 130.3, 126.7, 124.3, 121.9, 118.4, 114.2, 69.1. **HR-MS** (ESI) calcd for $C_{15}H_{11}BrO_4$ [M–H]$^-$: 320.97679, found: 320.97515.

Methyl 2-hydroxy-5-((3-nitrobenzyl)oxy)benzoate (52)

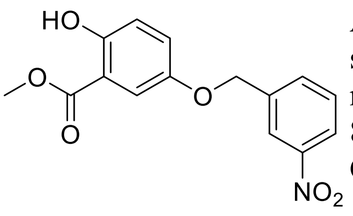

According to **GP-A, 51** (350 mg, 1.62 mmol) was added to a solution of **37** (300 mg, 1.78 mmol) and K_2CO_3 (269 mg, 1.94 mmol) in acetone to give after flash chromatography (*c*- Hex/EtOAc 85:15) **52** as a white solid (600 mg, 81%). **^{1}H-NMR** (500 MHz, $CDCl_3$-*d*) δ 10.41 (s, 1H), 8.33 (t, J=2.0 Hz, 1H), 8.20 (dd, J=7.6, 1.6 Hz, 1H), 7.77 (d, J=7.6 Hz, 1H), 7.58 (t, J=7.9 Hz, 1H), 7.40 (d, J=3.2 Hz, 1H), 7.17 (dd, J=9.1, 3.2 Hz, 1H), 6.95 (d, J=9.1 Hz, 1H), 5.12 (s, 2H), 3.96 (s, 3H).

^{13}C-NMR (126 MHz, $CDCl_3$-*d*) δ 170.2, 156.8, 150.7, 148.6, 139.2, 133.3, 129.8, 124.8, 123.2, 122.4, 119.0, 113.7, 112.2, 69.7, 52.6. **LC-MS** calcd for $C_{15}H_{14}NO_6$ $[M+H]^+$: 304.082, found 304.1.

Methyl 5-((3-aminobenzyl)oxy)-2-hydroxybenzoate (53)

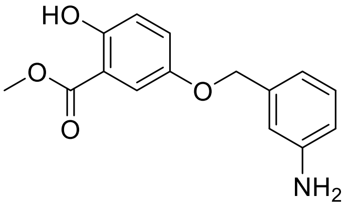

According to **GP-E**, using compound **52** (350 mg, 1.15 mmol), NH_4Cl (0.380 g, 6.92 mmol) and iron powder (387 mg, 6.92 mmol) afforded after flash chromatography (*c*-Hex/EtOAc 70:30) **53** as a yellow solid (240 mg, 70%). **^{1}H-NMR** (500 MHz, DMSO-d_6) δ 10.08 (s, 1H), 7.30 (d, *J*=3.1 Hz, 1H), 7.20 (dd, *J*=9.0, 3.2 Hz, 1H), 7.00 (t, *J*=7.7 Hz, 1H), 6.93 (d, *J*=9.0 Hz, 1H), 6.55 – 6.52 (m, 1H), 6.49 (dd, *J*=8.0, 1.9 Hz, 1H), 5.09 (s, 2H), 4.90 (s, 2H), 3.88 (s, 3H). **^{13}C-NMR** (126 MHz, DMSO-d_6) δ 169.0, 154.3, 150.9, 148.8, 137.5, 128.9, 124.1, 118.5, 114.9, 113.6, 113.4, 112.8, 112.7, 70.2, 52.5. **LC-MS** calcd for $C_{15}H_{16}NO_4$ $[M+H]^+$: 274.1017, found 274.2.

Methyl 5-((3-azidobenzyl)oxy)-2-hydroxybenzoate (54)

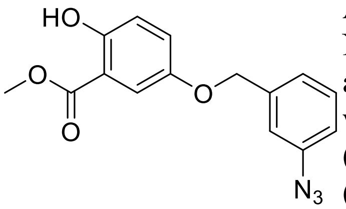

According to **GP-F**, using compound **53** (240 mg, 0.88 mmol), $NaNO_2$ (181 mg, 2.63 mmol) and NaN_3 (228 mg, 3.51 mmol), afforded after flash chromatography (*c*-Hex/EtOAc 80:20) **54** as a white solid (203 mg, 77%). **^{1}HNMR** (500 MHz, DMSO-d_6) δ 10.10 (s, 1H), 7.43 (t, *J*=7.8 Hz, 1H), 7.34 (d, *J*=3.2 Hz, 1H), 7.29–7.21 (m, 2H), 7.19 (st, *J*=2.0 Hz, 1H), 7.08 (dd, *J*=7.9, 2.3 Hz, 1H), 6.94 (d, *J*=9.0 Hz, 1H), 5.08 (s, 2H), 3.89 (s, 3H). **^{13}CNMR** (126 MHz, DMSO-d_6) δ 168.9, 154.5, 150.6, 139.5, 139.3, 130.1, 124.3, 124.1, 118.6, 118.5, 118.0, 113.7, 112.8, 69.2, 52.5. **LC-MS** calcd for $C_{15}H_{12}N_3O_4$ $[M–H]^-$: 298.083, found 298.1.

5-((3-Azidobenzyl)oxy)-2-hydroxybenzoic acid (55)

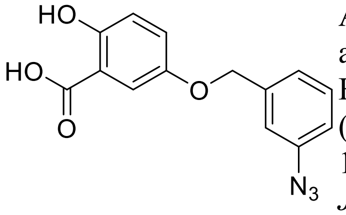

According to **GP-B**, using **54** (200 mg, 0.668 mmol) and NaOH (in aq. 10%, 0.8 mL), afforded after flash chromatography (c-Hex/EtOAc 70:30) **55** as an off-white solid (170 mg, 90%). **^{1}HNMR** (500 MHz, DMSO-d_6) δ 7.43 (t, *J*=7.8 Hz, 1H), 7.34 (d, *J*=3.2 Hz, 1H), 7.26 (d, *J*=7.6 Hz, 1H), 7.23 (dd, *J*=9.0, 3.2 Hz, 1H), 7.19 (d, *J*=2.0 Hz, 1H), 7.08 (dd, *J*=7.9, 2.4 Hz, 1H), 6.91 (d, *J*=9.0 Hz, 1H), 5.08 (s, 2H). **^{13}CNMR** (126 MHz, DMSO-d_6) δ 206.8, 171.5, 155.6, 150.4, 139.4, 130.1, 124.2, 124.2, 118.5, 118.2, 118.0, 113.9, 112.7, 69.2. **LC-MS** calcd for $C_{16}H_{12}N_3O_4$ $[M–H]^-$: 310.1, found 310.1.

1-Bromo-3-(bromomethyl)-5-nitrobenzene (57)

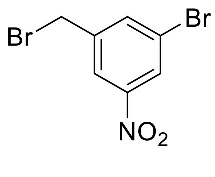

According to **GP-D**, **56** (2.00 g, 8.62 mmol), pyridine (0.7 mL, 8.62 mmol) and phosphorus tribromide (4.1 mL, 43.10 mmol) afforded after flash chromatography (*c*-Hex/EtOAc 13:1) **57** as a light yellow solid (3.30 g, 65%). **^{1}H NMR** (500 MHz, $CDCl_3$-*d*) δ 8.32 (s, 1H), 8.21 (s, 1H), 7.88 (s, 1H), 4.49 (s, 2H). **^{13}C NMR** (126 MHz, $CDCl_3$-*d*) δ 148.8, 141.3, 137.8, 126.5, 123.1, 122.6, 29.9. **LC-MS** calcd for $C_7H_4Br_2NO_2$ $[M–H]^-$: 291.9, found: 293.9.

Methyl 5-((3-bromo-5-nitrobenzyl)oxy)-2-hydroxybenzoate (58)

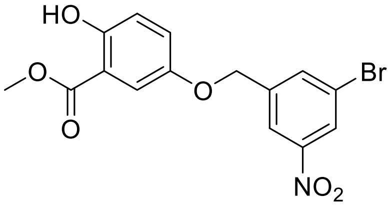

According to **GP-A**, using **57** (3.30 g, 11.19 mmol), **37** (1.71 g, 10.17 mmol) and K_2CO_3 (1.69 g, 12.21 mmol), afforded after flash chromatography (*c*-Hex/EtOAc 6:1) **58** as a yellow solid (3.00 g, 77%). **^{1}H NMR** (500 MHz, DMSO-d_6) δ 10.12 (s, 1H), 8.35 (t, 1H, *J*=1.8 Hz), 8.31 (s, 1H), 8.14 (s, 1H), 7.38 (s, 1H), 7.29 (dd, 1H, *J*=3.2, 9.0 Hz), 6.97 (d, 1H, *J*=9.0 Hz), 5.22 (s, 2H), 3.90 (s, 3H). **^{13}C NMR** (126 MHz, DMSO-d_6) δ 168.8, 154.7, 150.2, 148.6, 141.5, 136.4, 125.4, 124.1, 122.0, 121.2, 118.7, 113.9, 112.9, 68.0, 52.5. **LC-MS** calcd for $C_{15}H_{11}BrNO_6$ [*M*–H]$^-$: 380.0, found: 380.0.

Methyl 5-((3-(5-chloro-6-isopropoxypyridin-3-yl)-5-nitrobenzyl)oxy)-2-hydroxybenzoate (59)

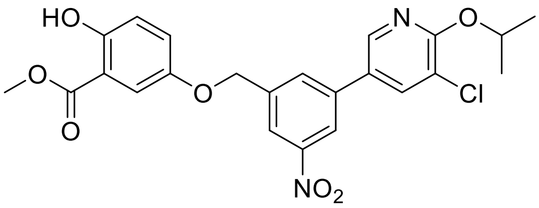

According to **GP-C**, using **58** (1.20 g, 3.14 mmol), (4-chloro-6-isopropoxypyridin-3-yl)boronic acid (0.81 g, 4.41 mmol), K_2CO_3 (1.48 g, 10.68 mmol) and [Pd(dppf)Cl_2] (0.11 g, 0.16 mmol), afforded after flash chromatography (*c*-Hex/EtOAc 6:1) **59** as a yellow solid (1.00 g, 68%). **^{1}H NMR** (500 MHz, DMSO-d_6) δ 10.12 (s, 1H), 8.58 (d, 1H, *J*=2.1 Hz), 8.49 (s, 1H), 8.40 (d, 1H, *J*=2.3 Hz), 8.29 (d, 2H, *J*=6.0 Hz), 7.42 (d, 1H, *J*=3.2 Hz), 7.32 (dd, 1H, *J*=3.1, 9.0 Hz), 6.97 (d, 1H, *J*=9.0 Hz), 5.38 (m, 1H), 5.27 (s, 2H), 3.90 (s, 3H), 1.37 (d, 6H, *J*=6.1 Hz). **^{13}C NMR** (126 MHz, DMSO-d_6) δ 168.8, 158.0, 154.6, 150.4, 148.6, 143.5, 140.1, 137.6, 137.4, 132.0, 127.8, 124.2, 121.1, 120.6, 118.7, 117.7, 113.9, 112.9, 69.7, 68.6, 52.5, 21.8. **LC-MS** calcd for $C_{23}H_{20}ClN_2O_7$ [*M*–H]$^-$: 471.1, found: 471.1.

Methyl 5-((3-amino-5-(5-chloro-6-isopropoxypyridin-3-yl)benzyl)oxy)-2-hydroxybenzoate (60)

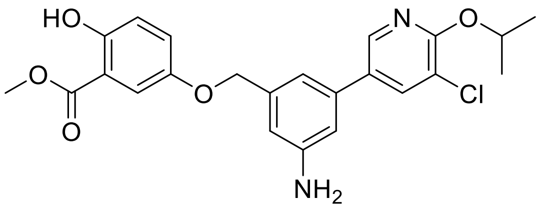

According to **GP-E**, using **59** (0.73 g, 1.54 mmol), NH_4Cl (0.50 g, 9.26 mmol) and iron powder (0.52 g, 9.26 mmol), afforded after flash chromatography (*c*-Hex/EtOAc 3:1) **60** as a brown oil (0.57 g, 83%). **^{1}H NMR** (500 MHz, DMSO-d_6) δ 10.09 (s, 1H), 8.30 (d, 1H, *J*=2.1 Hz), 8.02 (d, 1H, *J*=2.1 Hz), 7.34 (d, 1H, *J*=3.1 Hz), 7.23 (dd, 1H, *J*=1.0 Hz), 6.93 (d, 1H, *J*=9.0 Hz), 6.87 (s, 1H), 6.77 (s, 1H), 6.66 (s, 1H), 5.35-5.33 (m, 1H), 4.97 (s, 2H), 3.88 (s, 3H), 1.34 (d, 6H, *J*=6.1 Hz). **^{13}C NMR** (126 MHz, DMSO-d_6) δ 168.9, 157.2, 154.4, 150.8, 149.4, 142.4, 138.5, 136.6, 136.3, 130.8, 124.1, 118.5, 117.3, 113.7, 113.4, 112.7, 112.4, 111.2, 70.1, 69.3, 52.5, 21.8. **LC-MS** calcd for $C_{23}H_{24}ClN_2O_5$ [*M*+H]$^+$: 443.1, found: 443.1.

Methyl 5-((3-azido-5-(5-chloro-6-isopropoxypyridin-3-yl)benzyl)oxy)-2-hydroxybenzoate (61)

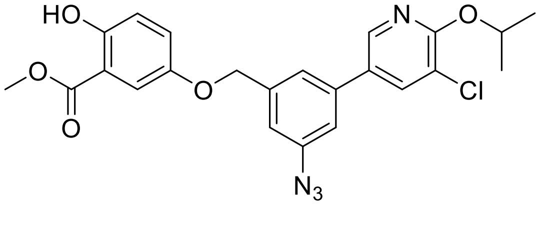

According to **GP-F**, using **60** (0.55 g, 1.25 mmol), $NaNO_2$ (0.26 g, 3.75 mmol) and NaN_3 (0.32 g, 4.99 mmol), afforded **61** that was used without further purification in the next step (0.50 g, 85%) **^{1}H NMR** (500 MHz, DMSO-d_6) δ 10.11 (s, 1H), 8.48 (d, 1H, *J*=2.1 Hz), 8.27 (d, 1H, *J*=2.1 Hz), 7.61 (s, 1H), 7.40

(s, 1H), 7.37 (d, 1H, *J*=3.2 Hz), 7.28 (dd, 1H, *J*=3.1, 9.0 Hz), 7.19 (s, 1H), 6.95 (d, 1H, *J*=9.2 Hz), 5.36 (m, 1H), 5.12 (s, 2H), 3.89 (s, 3H), 1.35 (d, 6H, *J*=6.1 Hz). **^{13}C NMR** (126 MHz, DMSO-d_6) δ 168.9, 157.7, 154.5, 150.5, 143.1, 140.5, 139.9, 137.6, 137.1, 128.9, 124.2, 122.5, 118.6, 117.5, 117.3, 116.6, 113.8, 112.8, 69.5, 69.2, 52.5, 21.8. **LC-MS** calcd for $C_{23}H_{22}ClN_4O_5$ [M+H]$^+$: 469.1 found: 469.1.

5-((3-Azido-5-(5-chloro-6-isopropoxypyridin-3-yl)benzyl)oxy)-2-hydroxybenzoic acid (62)

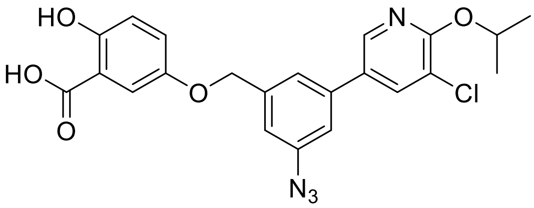

According to **GP-B**, using **61** (0.62 g, 1.33 mmol) and NaOH (sol. 10%, 5.3 mL), afforded after flash chromatography (*c*-Hex/EtOAc 9:1) **62** as a light brown solid (0.50 g, 84%). **^{1}H NMR** (500 MHz, DMSO-d_6) δ 8.47 (d, 1H, *J*=2.1 Hz), 8.26 (d, 1H, *J*=2.1 Hz), 7.60 (s, 1H), 7.40 (s, 1H), 7.38 (d, 1H, *J*=3.2 Hz), 7.26 (dd, 1H, *J*=3.1, 8.9 Hz), 7.19 (s, 1H), 6.91 (d, 1H, *J*=9.0 Hz), 5.35 (m, 1H), 5.12 (s, 2H), 1.35 (d, 6H, *J*=6.1 Hz). **^{13}C NMR** (126 MHz, DMSO-d_6) δ 171.5, 157.7, 155.7, 150.4, 143.1, 140.5, 140.1, 137.7, 137.1, 129.0, 124.2, 122.5, 118.2,117.6, 117.4, 116.7, 114.1, 112.9, 69.6, 69.2, 21.9. **LC-MS** calcd for $C_{22}H_{18}ClN_4O_5$ [M–H]$^-$: 453.1 found: 453.1.

2.0 Molecular docking

2.1 Protein Preparation

The protein structure was retrieved in the apo crystal structure (PDB ID: 6ZG3, 2.80 Å) and pre-processed with Protoss[1] to add missing hydrogens and assign protonation and tautomeric states.
Two binding sites were identified based on prior unbiased coarse-grained molecular dynamics (CG-MD) simulations.[2] Among them, we selected the P9 site, located at the interface of the S-component and the EcfT module, based on short MD simulations comparing the two sites.[3] P9 is located between the S-component and the EcfT of the cell membrane.

2.2 Docking details

Docking was performed using the FlexX docking engine version 6.0.[4] A total of 30 poses were generated for each compound, which were subsequently evaluated using the HYDE scoring function.[5] The pose chosen for each compound was the one that best aligned with the pose of **1** within the binding site, even if it had a lower HYDE score than the top-ranked pose (Table S1). For compound **33** it has been selected the pose number 27 as it best aligned with the pose of **1** within the binding site.

Protein–ligand interactions between the poses and the ECF-PanT structure were assessed using the protein-ligand interaction profiler (PLIP)[6] and, additionally, inspected in PyMOL Molecular Graphics System version 3.[7]

Table S1. Top scoring pose for compounds **1**, **18**, **33** and **36**.

Compound	Top pose score	Selected pose score [nM] (pose nr and RMSD to top pose [Å])
**1**	1818.6	1818.6 (1, 0.0)
**18**	0.001	0.001 (1, 0.0)
**33**	0.071	59.83 (27, 3.6)
**36**	0.005	0.005 (1, 0.0)

3.0 Biological evaluation of synthesized compounds

3.1 Proteoliposome uptake assay

To assess the effectiveness of the compounds inhibiting the ECF proteins, we performed an uptake assay based on the one developed by Swier *et al.*[8] In this assay, the ECF transporters for folate (ECF-FolT2) and pantothenate (ECF-PanT) from *Lactobacillus delbrueckii* were used and reconstituted into lipid vesicles known as proteoliposomes. Figure S1 exhibits the percentage of inhibition of transport for each compound at 150 µM (S1A, ECF-FolT2 and S1B, ECF-PanT).

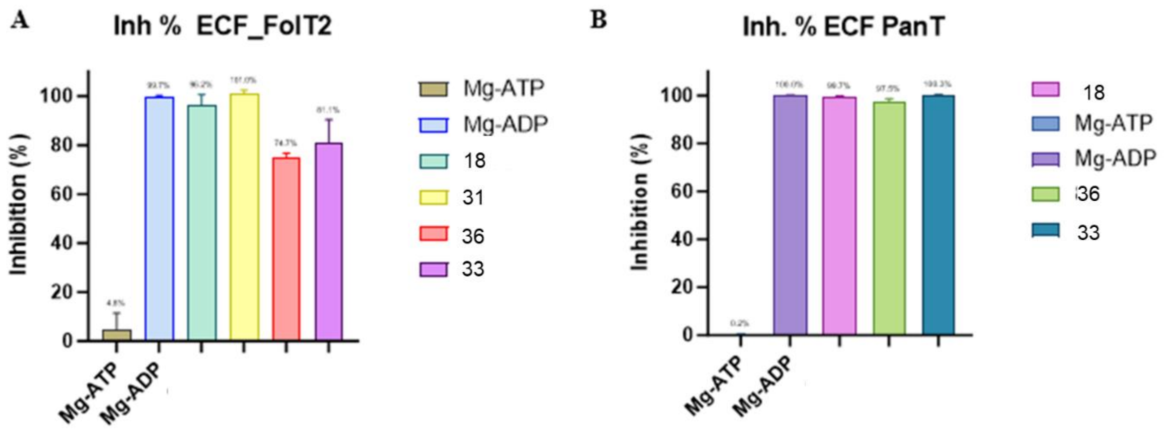

Figure S1: The graphs display inhibition percentages for the compounds at a concentration of 150 µM, obtained through the proteoliposome uptake assay. A) Percentage of inhibition by indicated compounds of folate transport catalized by ECF-FolT2 from *Lactobacillus delbrueckii* and B Percentage of inhibition by indicated compounds of pantothenate transport by ECF-PanT from *Lactobacillus delbrueckii*.

3.2 Whole-cell bacterial uptake assay (ECF-T assay)

The ECF inhibitors and their inhibitory effect on folate uptake in the Gram-positive model organism *Lactobacillus casei* were studied in a whole-cell uptake assay as recently published in our group.[9] For this purpose, 20 µL of inhibitor was added to the wells of a MultiScreen HTS Filter Plate containing 175 µL of *L. casei* culture diluted in citrate buffer. The blank was determined with 185 µL citrate buffer and 10 µL of DMSO. 5 µL of radiolabeled folic acid was added to each well (2 µM, Moravek Biochemicals, Brea, CA). Using this method, we determined the percentage of inhibition at 12.5, 50, 100 and 200 µM for the compounds and the IC_{50} values for our best compounds. The

assay was done in both technical and biological duplicate ensuring the reliability and consistency of the data. Subsequently, the gathered data were analyzed using GraphPad Prism, a robust statistical software tool.

3.3 Cytotoxicity assays

To obtain information regarding the toxicity of our compounds, their impact on the viability of human cells was investigated as reported previously[10] with some adaptions. HepG2 or A549 cells ($2x10^4$ cells per well) were seeded in 96-well, flat-bottomed culture plates in 100 μL culture medium (DMEM containing 10% fetal calve serum, 1% penicillin-streptomycin). Twenty-four hours after seeding the cells, medium was removed and replaced by medium containing test compounds in a final DMSO concentration of 1%. Compounds were tested in duplicates at a single concentration or, for CC_{50} determination, at 8 concentrations that were prepared via 2-fold serial dilutions in 1% DMSO/medium. Epirubicin and doxorubicin were used as positive controls in serial dilutions starting from 10 μM, and rifampicin was used as a negative control (at 100 μM). The living cell mass was determined 48 h after treatment with compounds by adding 0.1 volumes of 3-(4,5-dimethylthiazol-2-yl)-2,5-diphenyltetrazolium bromide (MTT) solution (5 mg/mL sterile PBS) (Sigma, St. Louis, MO) to the wells. After incubating the cells for 30 min at 37 °C (atmosphere containing 5% CO_2), medium was removed and MTT crystals were dissolved in 75 μL of a solution containing 10% SDS and 0.5% acetic acid in DMSO. The optical density (OD) of the samples was determined photometrically at 570 nm in a PHERAstar Omega plate reader (BMG labtech, Ortenberg, Germany). To obtain percent viability for each sample, their ODs were related to those of DMSO controls. At least two independent measurements were performed for each compound. The calculation of CC_{50} was performed using the nonlinear regression function of GraphPad Prism 10 (GraphPad Software, San Diego, CA, USA).

3.4 Antibacterial assays

All microorganisms were obtained from the German Collection of Microorganisms and Cell Cultures (DSMZ) or the American Tpye Culture Collection (ATCC) and were handled according to standard procedures. Bacteria were inoculated into tryptic soy broth (TSB) to obtain a final inoculum of 10^5 colony-forming units (CFU)/mL.

The tested compounds were prepared as DMSO stocks (20 mM). Serial dilutions of derivatives in the growth medium (0.06 to 128 μM) were prepared in sterile 96-well plates and the bacterial suspensions were added. Growth inhibition was assessed after static incubation at 37 °C for 24 h. *Streptococcus pneumoniae* was grown at 5% CO_2. Minimum inhibitory concentrations (MIC) are defined as the lowest compound concentration where no visible growth is observed.

Tables S2. Data plot of IC_{50} in the whole-cell uptake assay [µM] *vs* Minimum Inhibitory Concentration (MIC) [µM]

		Minimum Inhibitory Concentration (MIC) [µM]				
Code	IC_{50} (µM)	*E. faecium* DSM-20478	*E. faecium* DSM-17050	*E. faecium* DSM-20477	*S. pneumoniae* DSM 11865	*S. pneumoniae* DSM 20566
**3**	16	11	6	11	6	3
**23**	n.i.	>128	>128	>128	>128	>128
**24**	n.i.	>128	>128	>128	>128	>128
**18**	4	5	9	5	2	2
**31**	5	2	2	2	1	1
**33**	4	2	5	2	1	1
**34**	4	2	2	2	1	1
**36**	7	6	2	2	1	1
R^2 of correlation IC_{50} *vs* MIC		0.8482	0.0247	0.7861	0.8695	0.6570

n.i.: no inhibition

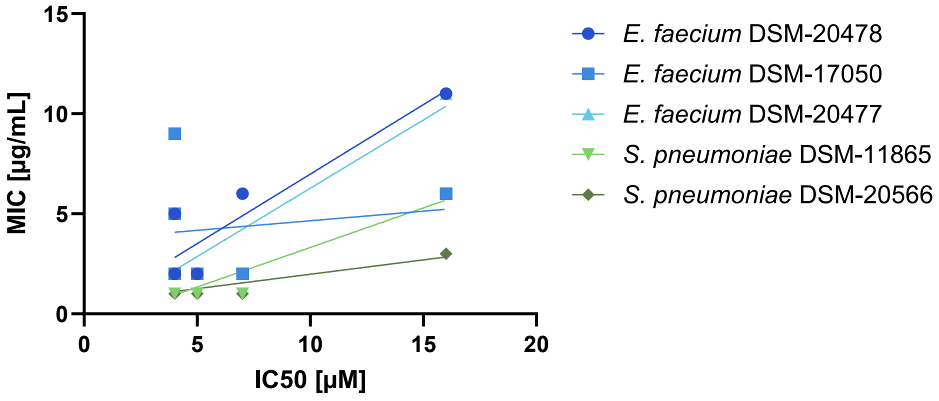

Figure S2: Correlation of Minimum Inhibitory Concentration (MIC)values *vs* IC_{50} in whole-cell assays.

3.5 Time-kill kinetics

For studying time-kill kinetics with *Enterococcus faecium* ATCC51559, an overnight bacterial culture was prepared from single colonies grown on CASO-Agar in sterile fresh medium (Tryptic Soy Broth, TSB) and placed in a shaking incubator at 180 rpm at 37 °C. The overnight bacterial culture was diluted 1/100 in sterile fresh medium TSB. After 3-4 hours of incubation, the OD_{600} was determined and the initial inoculum was adjusted to 5 x 10^6 colony forming units (CFU)/mL. Compound **36** was dissolved in DMSO (dimethylsulfoxide, Sigma) and the concentrations were adjusted to achieve 1% (*v/v*) DMSO for all samples in the assay. Time-kill kinetics were determined at 2x, 4x and 8x MIC of **36** and at 4x MIC of Ciprofloxacin (CIP) as control antibiotic, respectively, and compared to 1% DMSO as growth control. The treated bacterial culture was incubated in a shaking incubator at 180 rpm at 37 °C. At the designated time points (0, 1, 2, 3, 4, 6, and 24 hours), aliquots were taken and CFU/mL were determined

by plating the culture on non-selective CASO agar in serial dilutions. Plates were incubated in a static incubator at 37°C for 24 hours. After incubation, colonies were counted and CFU/mL were determined and plotted as time-kill curve (TKC).

4.0 *In vitro* ADME assays

4.1 Kinetic Turbidimetric Solubility

The desired compounds were sequentially diluted in DMSO in a 96-well plate. 1.5 µL of each well were transferred into another 96-well plate and mixed with 148.5 µL of PBS. Plates were shaken for 5 min at 600 rpm at room temperature (r.t.), and the absorbance at 620 nm was measured. Absorbance values were normalized by blank subtraction and plotted using GraphPad Prism 8.4.2 (GraphPad Software, San Diego, CA, USA). Solubility (S) was determined based on the First X value of AUC function using a threshold of 0.005.

4.2 Lipophilicity Determination

$LogD_{7.4}$ was analyzed using an HPLC−based method. The UV retention time of reference compounds with known $LogD_{7.4}$ was determined and plotted toward their $LogD_{7.4}$. Linear regression was used to determine the $LogD_{7.4}$ of unknown compounds. Analysis was performed using a Vanquish Flex HPLC system with variable wavelength detector (Thermo Fisher, Dreieich, Germany) with the following conditions: EC150/2 NUCLEODUR C18 Pyramid column, 5 µM (Macherey Nagel, Düren, Germany); eluent A: 50 mM NH_4OAc pH 7.4, eluent B: acetonitrile, and flow: 0.6 mL/min. The gradient was set to 0−100% B from 0 to 2.5 min, 100% B from 2.5 to 3.0 min, 100−0% B from 3.0 to 3.2 min, and 0% B from 3.2−5.0

4.3 Metabolic Stability in Liver S9 Fractions

For the evaluation of combined phase I and phase II metabolic stability, the compound (1 µM) was incubated with 1 mg/mL pooled mouse liver S9 fraction (Xenotech, Kansas City, USA) or human liver S9 fraction (Corning, USA), 2 mM NADPH, 1 mM UDPGA, 10 mM $MgCl_2$, 5 mM GSH and 0.1 mM PAPS at 37 °C for 240 min. The metabolic stability of testosterone, verapamil and ketoconazole were determined in parallel to confirm the enzymatic activity of mouse S9 fractions, for human S9 testosterone, diclofenac and propranolol were used. The incubation was stopped after defined time points by precipitation of aliquots of S9 enzymes with 2 volumes of cold acetonitrile containing internal standard (150 nM diphenhydramine or 15 nM for Altis Plus measurements). Samples were stored on ice until the end of the incubation and precipitated protein was removed by centrifugation (15 min, 4 °C, 4,000 g). Concentration of the remaining test compound at the different time points was analyzed by HPLC-MS/MS (Ultimate 3000 coupled to TSQ Quantum Access MAX, or Vanquish Flex coupled to a TSQ Altis Plus, Thermo Fisher, Dreieich, Germany) and used to determine half-life ($t_{1/2}$).

Table S3. *In vitro* metabolic stability in mouse/human liver S9.

Parameters	Compound				
	**3**	**18**	**31**	**33**	**36**
Ligand efficiency (LE)[a]	0.26	0.26	0.19	0.19	0.19
Lipophilic ligand efficiency (LLE)[b]	5.23	0.74	3.29	2.00	3.42
Mouse liver S9 $t_{1/2}$[c] [min]/Cl_{int}[d] [µL/min/mg]	94±24/ 7.7±2.1	>120/ <5.8	>120/ <5.8	>120/ <5.8	>120/ <5.8
Human liver S9 $t_{1/2}$[c][min]/Cl_{int}[d] [µL/min/mg]	12.1/ 57.4	>120/ <5.8	>120/ <5.8	n.d.	>120/ <5.8

[a]Calculated via the following formula: 1.4 *pIC_{50}/number of heavy atoms; [b]LLE = pIC_{50} – logD; [c]Half-life; [d]Intrinsic clearance; n.d.: not determined. Means ± SD of at least 2 independent measurements are shown for mouse liver S9; human S9 was measured once.

4.4 Metabolic Stability in Liver Microsomes

For the evaluation of phase I metabolic stability, the compound (1 µM) was incubated with 0.5 mg/mL pooled mouse or human liver microsomes (Xenotech, Kansas City, USA), 2 mM NADPH, 10 mM $MgCl_2$ at 37 °C for 120 min on a microplate shaker (Eppendorf, Hamburg, Germany). The metabolic stability of testosterone, verapamil and ketoconazole were determined in parallel to confirm the enzymatic activity of mouse liver microsomes. For human liver microsomes, testosterone, diclofenac and propranolol were used. The incubation was stopped after defined time points by precipitation of aliquots of the incubation mixture with 2 volumes of cold acetonitrile containing internal standard (150 nM diphenhydramine or 15 nM for Altis Plus measurements). Samples were stored on ice until the end of the incubation and precipitated protein was removed by centrifugation (15 min, 4 °C, and 4,000 g). Concentration of the remaining test compound at the different time points was analyzed by HPLC-MS/MS (Ultimate 3000 coupled to TSQ Quantum Access MAX, or Vanquish Flex coupled to a TSQ Altis Plus, Thermo Fisher, Dreieich, Germany) and used to determine half-life ($t_{1/2}$).

4.5 Stability in Plasma

To determine stability in mouse plasma, the compound (1 µM) was incubated with pooled CD-1 mouse or human plasma (Neo Biotech, Nanterre, France). Samples were taken at defined time points by mixing aliquots with 4 volumes of acetonitrile containing internal standard (125 nM diphenhydramine or 12.5 nM for Altis Plus measurements). Samples were stored on ice until the end of the incubation and precipitated protein was removed by centrifugation (15 min, 4 °C, 4,000 g, 2 centrifugation steps). Concentration of the remaining test compound at the different time points was analyzed by HPLC-MS/MS (Ultimate 3000 coupled to TSQ Quantum Access MAX, or Vanquish Flex coupled to a TSQ Altis Plus, Thermo Fisher, Dreieich, Germany) and used to determine half-life ($t_{1/2}$). The plasma stability of procain, propantheline and diltiazem were determined in parallel to confirm the enzymatic activity.

4.6 hERG Potassium Channel Assay

Electrophysiological assay was conducted to profile the compounds for activities on the hERG Potassium Channel Assay using the Qube electrophysiological platform. The assay has been performed by Eurofins Discovery.
After whole cell configuration is achieved, the cell is held at -80mV. The cell is depolarized to +40mV for 500ms and then to -80mV over a 100ms ramp to elicit the hERG tail current. This paradigm is delivered once every 5s to monitor the current amplitude.
All compounds were tested in the presence of 0.1% Non-Ionic Surfactant and at approximately room temperature. The parameters measured were the maximum tail current evoked on stepping to 40mV and ramping back to -80mV from the test pulse. All data were filtered for seal quality, seal drop, and current amplitude. The peak current amplitude was calculated before and after compound addition and the amount of block was assessed by dividing the Test compound current amplitude by the Control current amplitude. Control data is the mean hERG current amplitude collected 15 seconds at the end of the control period. Test compound data is the mean hERG current amplitude collected 15 seconds at the end of each 5-minute test compound application for each concentration.

5.0 *In vivo* data

5.1 *Galleria mellonella* infection model

G. mellonella larvae were utilized as an in vivo model to assess the activity of selected ECF inhibitors, following the protocols established by Alhayek et al. (2022)[11] and adapted and optimized for ECF transporters by Shams *et al.* (2024)[12]. *Streptococcus pneumoniae DSM 20566* was cultured in Todd-Hewitt medium containing 0.1% choline at 37 °C with 5% CO_2 until reaching an OD600 of 0.6–1, after which it was concentrated to an OD600 of 1.5 or higher. Groups of 10–15 larvae were infected by injecting 10 µL of the respective test condition into the left proleg, and the larvae were incubated at 37 °C with 5% CO_2 for 72–96 hours. Survival was monitored daily. Control groups included larvae injected with PBS, PBS supplemented with 1% DMSO, or left uninjected. The assay was conducted in three independent experiments, using a total of 40 larvae. Larvae were sourced from VALOMOLIA Company, Strasbourg, France.

Toxicity assessment has been done with compounds **18** and **36** (Figure S3).

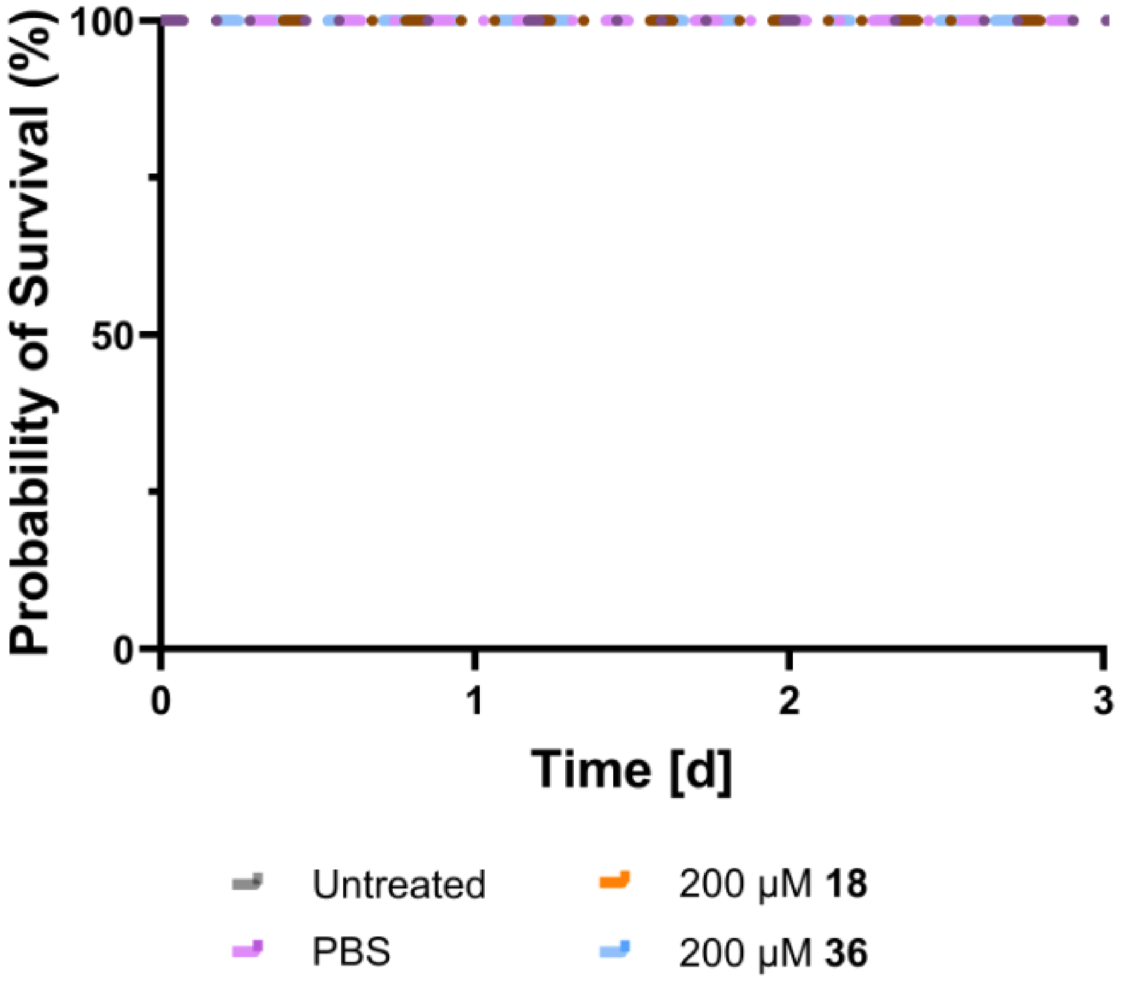

Figure S3. The larvae were injected in the absence and presence of compounds **18** and **36** at 200 µM. The control groups are with no injection and PBS.

5.2 *Danio rerio* (zebrafish) infection models

5.2.1 Zebrafish husbandry and embryo handling

The rearing of adult zebrafish and all experiments with zebrafish larvae were performed in accordance with EU Directive 2010/63/EU and internal guidelines based on the German Animal Welfare Act (§11 Abs. 1 TierSchG). The experiments were performed with the wild-type AB line that was originally purchased from the European Zebrafish Resource Centre at the Karlsruhe Institute of Technology (EZRC, KIT Karlsruhe, Germany). Zebrafish were bred in the original tank by adding a mating cage. Eggs were collected by pouring over a plastic sieve and maintained in fresh 0.3x Danieau's (17.4 mM NaCl, 0.21 mM KCl, 0.12 mM $MgSO_4$, 0.18 mM $Ca(NO_3)_2$, 1.5 mM HEPES, 1.2 µM methylene blue, pH 7.1-7.3) at 28°C. At 24 hpf, 0.3x Danieau's supplemented with 200 µM 1-phenyl-2-thiourea (PTU) (P7629, Sigma Aldrich) was added to the larvae to chemically prevent melanization by reducing tyrosinase activity. Zebrafish embryos were infected at 48 hpf. For this, embryos were anaesthetized by immersion in 0.25 mg/mL tricaine (MS-222, E10521, Sigma Aldrich). At a maximum of 120 hpf, embryos were euthanized by immersion in iced water to induce hypothermic shock.

5.2.2 Zebrafish infection model

A single colony of *E. faecium* ATCC51559 EGFP was picked from a selective plate (120 µg/mL spectinomycin, 30 µg/mL vancomycin) and grown overnight in tryptic soy broth (TSB) under the same selective pressure. To prepare a bacterial stock solution with a defined bacterial concentration, the solution was recultured (1/100) in TSB again supplemented with spectinomycin/vancomycin and incubated to grow to log phase OD600 = 0.6-0.8 and the cells were harvested by centrifugation at 4,000 rpm, 22 °C for 10 min. The pellet was resuspended in 4% PBS/PVP (m/v, phosphate buffered saline and polyvinylpyrrolidone, Sigma-Aldrich) and aliquots were stored at -80°C for further use. For the infection of zebrafish embryos, aliquots were thawed and mixed with 10 µL of phenol red (Sigma, P0290) to make a 1x injection solution (10,000 CFU/nL) and used immediately.

Microinjection needles were prepared using a micropipette puller (P-1000, Sutter Instrument, USA) and standard thin-walled glass capillaries (World Precision Instruments, TW100F-4) (parameters: pull 80, velocity 60, delay 90, pressure 200). For microinjection, a pulled glass capillary was filled with the bacterial suspension and fixed to the micromanipulator (M-152, Narishige, London, England). The tip was manually cut with a pair of tweezers, and the injection volume was calibrated to 2 nL by injecting single droplets into mineral oil on a microscale slide (Bresser, Rhede, Germany).

Zebrafish embryos were infected by microinjection of 20 000 CFU into the caudal vein (n = 10 embryos per infectious dose). Negative controls were untreated embryos.

Infected embryos were treated at 1 hour post infection (hpi) by adding the test items to the medium, which was renewed daily. Infected embryos were maintained in 0.3x Danieau's supplemented with 200 µM 1-phenyl-2-thiourea (PTU) at 28°C under different conditions, (i) non-treated, (ii) 400 µM Linezolid as positive control, (iii) 50 µM 36. Embryos were monitored daily for survival up to 72 hpi.

References

(1) Lippert, T.; Rarey, M. Fast Automated Placement of Polar Hydrogen Atoms in Protein–Ligand Complexes. *J. Cheminform.* **2009**, *1* (13) 1–13.
(2) Diamanti, E.; Souza, P. C. T.; Setyawati, I.; Bousis, S.; Monjas, L.; Swier, L. J. Y. M.; Shams, A.; Tsarenko, A.; Stanek, W. K.; Jäger, M.; et al. Identification of inhibitors targeting the energy-coupling factor (ECF) transporters. *Commun. Biol.* **2024**, *7* (1).
(3) Exapicheidou, I. A.; Shams, A.; Ibrahim, H.; Tsarenko, A.; Backenkohler, M.; Hamed, M. M.; Diamanti, E.; Volkamer, A.; Slotboom, D. J.; Hirsch, A.K.H. Hit optimization by dynamic combinatorial chemistry on *Streptococcus pneumoniae* energy-coupling factor transporter ECF-PanT. *Chem. Commun.* **2024**, *60* (7), 870-873.
(4) Rarey, M.; Kramer, B.; Lengauer, T.; Klebe, G. A Fast Flexible Docking Method using an Incremental Construction Algorithm. *J. Mol. Biol.* **1996**, *261* (3) 470–489.

(5) Reulecke, I.; Lange, G.; Albrecht, J.; Klein, R.; Rarey, M. Towards an Integrated Description of Hydrogen Bonding and Dehydration: Decreasing False Positives in Virtual Screening with the HYDE Scoring Function *ChemMedChem* **2008**, *3* (6), 885–897.

(6) Salentin, S.; Schreiber, S.; Haupt, J. V.; Adasme, M. F.; Schroeder, M. PLIP: fully automated protein–ligand interaction profiler *Nucleic Acids Res.* **2015**, *43*, (1).

(7) Schrödinger, LLC. The PyMOL Molecular Graphics System, Version 1.8. **2015**.

(8) Setyawati, I.; Stanek, W. K.; Majsnerowska, M.; Swier, L.; Pardon, E.; Steyaert, J.; Guskov, A.; Slotboom, D. J. In vitro reconstitution of dynamically interacting integral membrane subunits of energy-coupling factor transporters. *Elife* **2020**, *9,* e64389.

(9) Bousis, S.; Winkler, S.; Haupenthal, J.; Fulco, F.; Diamanti, E.; Hirsch, A.K.H. An Efficient Way to Screen Inhibitors of Energy-Coupling Factor (ECF) Transporters in a Bacterial Uptake Assay. *Int J Mol Sci* **2022**, *23* (5).

(10) Haupenthal, J.; Baehr, C.; Zeuzem, S.; Piiper, A. RNAse A-like enzymes in serum inhibit the anti-neoplastic activity of siRNA targeting polo-like kinase 1. *Int J Cancer* **2007**, *121* (1), 206-210.

(11) Alhayek, A.; Abdelsamie, A.S; Schönauer, E.; Camberlein, V.; Hutterer, E.; Posselt, G.; Serwanja, J.; Blöchl, C.; Huber, C. G.; Haupenthal, J.; Brandstetter, H.; Wessler, S.; Hirsch, A. K. H. Discovery and Characterization of Synthesized and FDA-Approved Inhibitors of Clostridial and Bacillary Collagenase *J. Med. Chem.* **2022** *65* (19), 12933-12955.

(12) Shams, A.; Bousis, S.; Diamanti, E.; Elgaher, W. A. M.; Zeimetz, L.; Haupenthal, J.; Slotboom, D. J.; Hirsch, A. K. H. Expression and characterization of pantothenate energy-coupling factor transporters as an anti-infective drug target. *Protein Sci.* **2024**, 33(11):e5195.

NMR spectra

Compound **30**

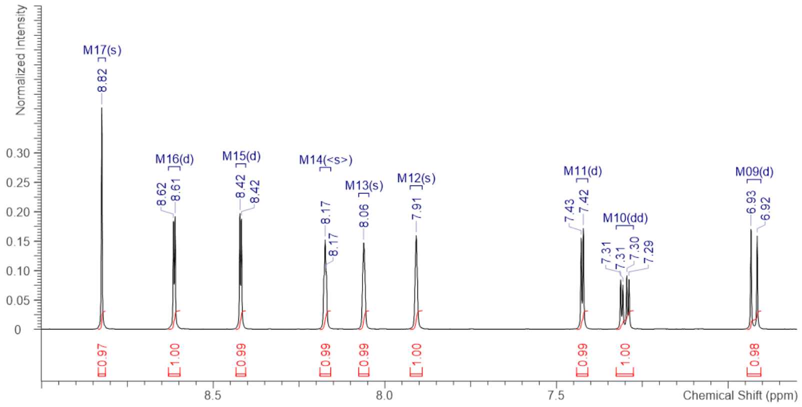

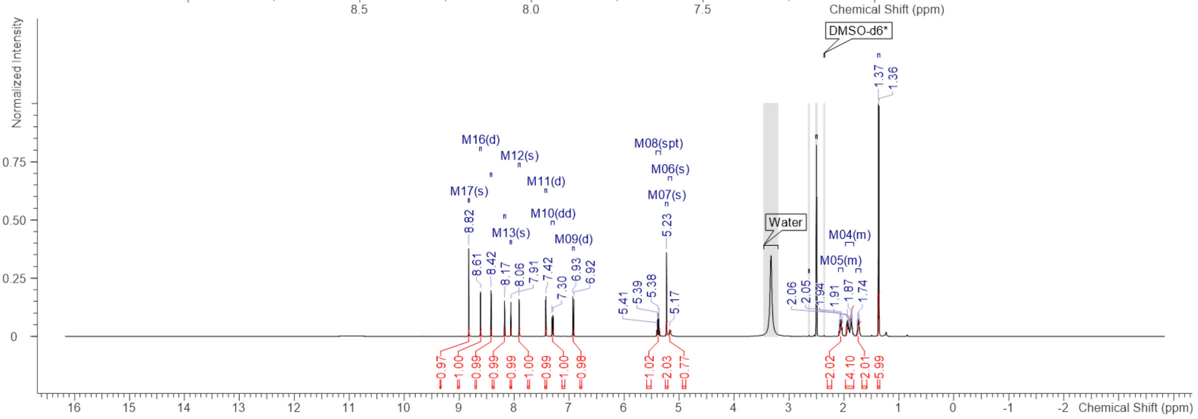

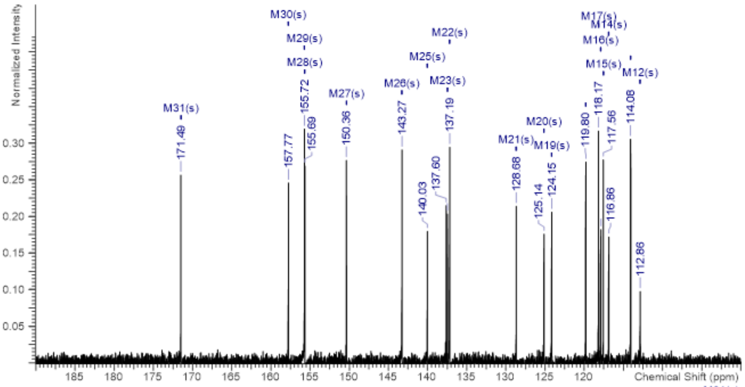

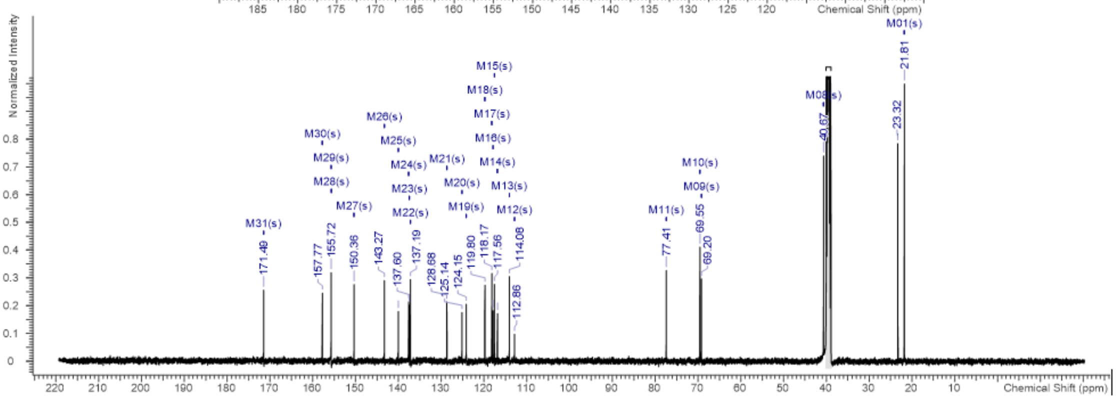

Compound **31**

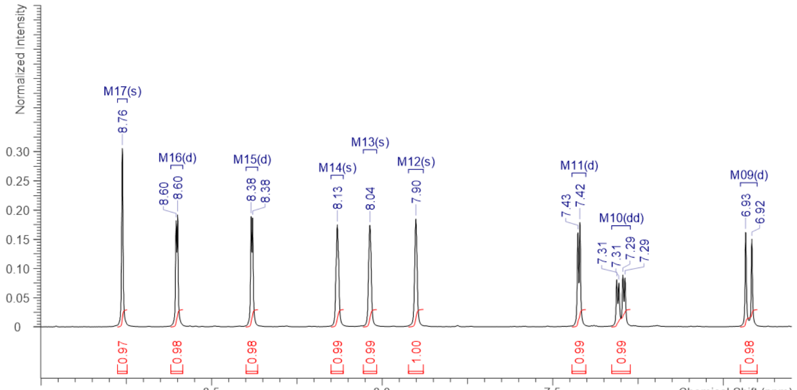
Normalized Intensity
M17(s)
M16(d)
M15(d)
M14(s)
M13(s)
M12(s)
M11(d)
M10(dd)
M09(d)
Chemical Shift (ppm)

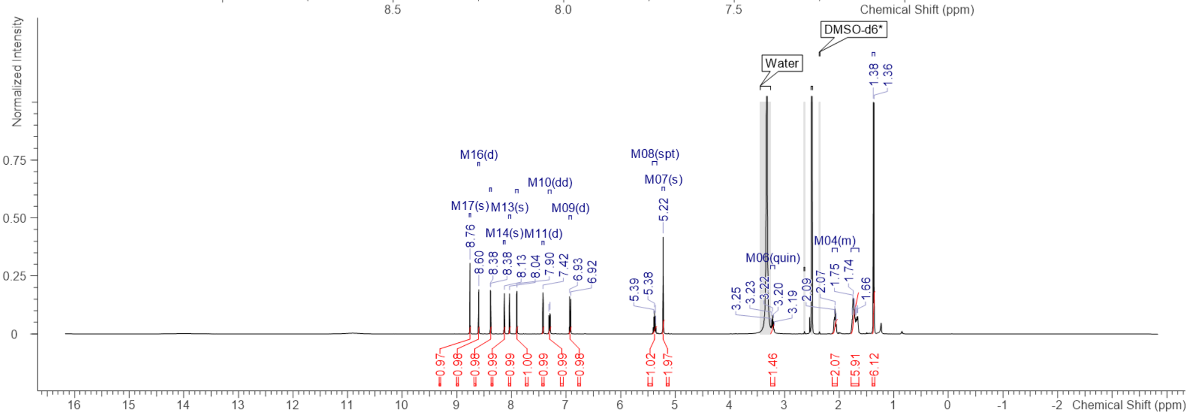
Normalized Intensity
DMSO-d6*
Water
M16(d)
M08(spt)
M07(s)
M17(s)
M13(s)
M10(dd)
M09(d)
M14(s)
M11(d)
M06(quin)
M04(m)
Chemical Shift (ppm)

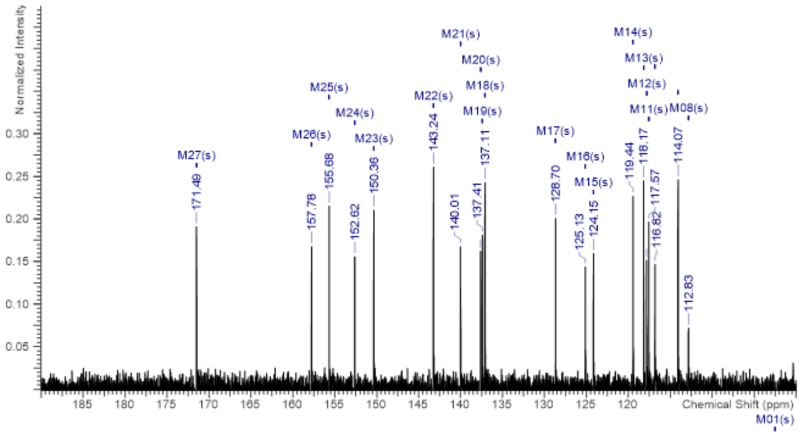
Normalized Intensity
M27(s)
M26(s)
M25(s)
M24(s)
M23(s)
M22(s)
M21(s)
M20(s)
M18(s)
M19(s)
M17(s)
M16(s)
M15(s)
M14(s)
M13(s)
M12(s)
M11(s)
M08(s)
Chemical Shift (ppm)

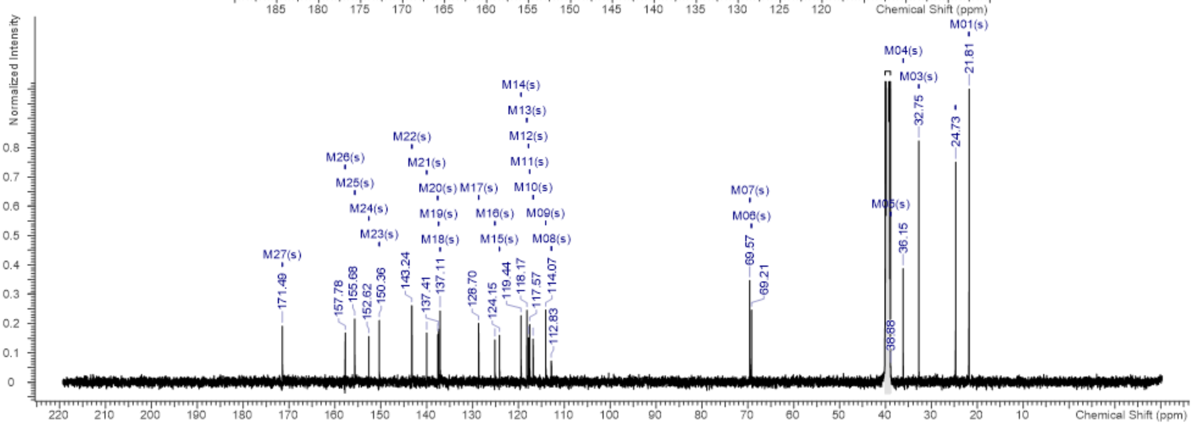
Normalized Intensity
M27(s)
M26(s)
M25(s)
M24(s)
M23(s)
M22(s)
M21(s)
M20(s)
M19(s)
M18(s)
M17(s)
M16(s)
M15(s)
M14(s)
M13(s)
M12(s)
M11(s)
M10(s)
M09(s)
M08(s)
M07(s)
M06(s)
M05(s)
M04(s)
M03(s)
M01(s)
Chemical Shift (ppm)

Compound **32**

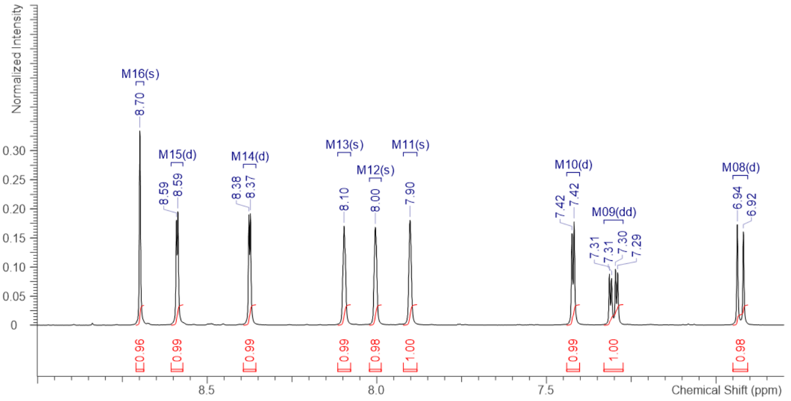
M16(s) 8.70
M15(d) 8.59 8.59
M14(d) 8.38 8.37
M13(s) 8.10
M12(s) 8.00
M11(s) 7.90
M10(d) 7.42 7.42
M09(dd) 7.31 7.31 7.30 7.29
M08(d) 6.94 6.92
0.96 0.99 0.99 0.99 0.98 1.00 0.99 1.00 0.98
Normalized Intensity
Chemical Shift (ppm)

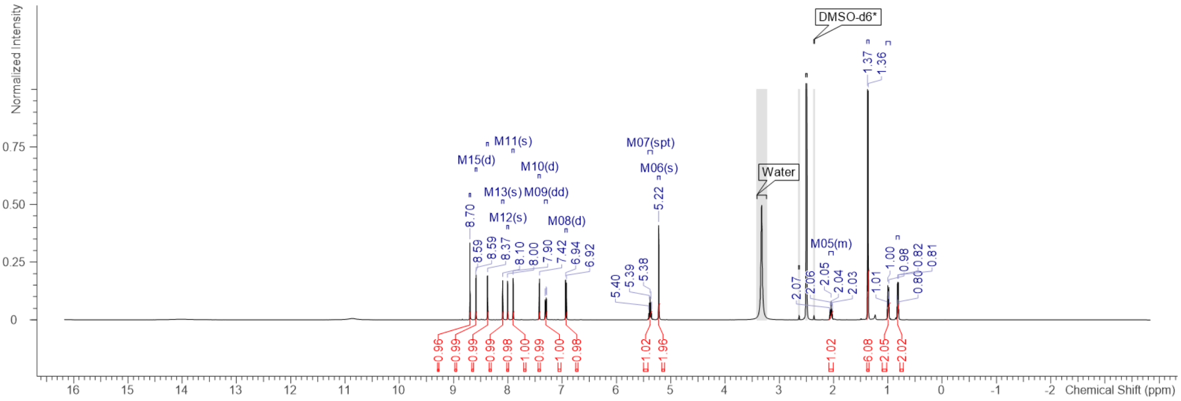
DMSO-d6*
Water
M15(d) M11(s) M10(d) M13(s) M09(dd) M12(s) M08(d)
M07(spt) M06(s)
M05(m)
8.70 8.59 8.59 8.37 8.10 8.00 7.90 7.42 6.94 6.92
5.40 5.39 5.38 5.22
2.07 2.06 2.05 2.04 2.03
1.37 1.36 1.01 1.00 0.98 0.82 0.81 0.80
0.96 0.99 0.99 0.99 0.98 1.00 0.99 1.00 0.98 1.02 1.96 1.02 6.08 2.05 2.02
Normalized Intensity
Chemical Shift (ppm)

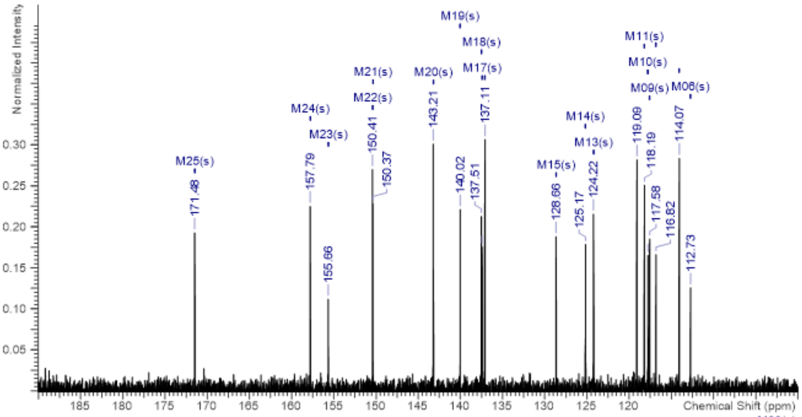
M25(s) 171.48
M24(s) M23(s) 157.79 155.66
M21(s) M22(s) 150.41 150.37
M20(s) 143.21
M19(s) M18(s) M17(s) 140.02 137.51 137.11
M15(s) 128.66
M14(s) M13(s) 125.17 124.22
M11(s) M10(s) M09(s) M06(s) 119.09 118.19 117.58 116.82 114.07 112.73
Normalized Intensity
Chemical Shift (ppm)

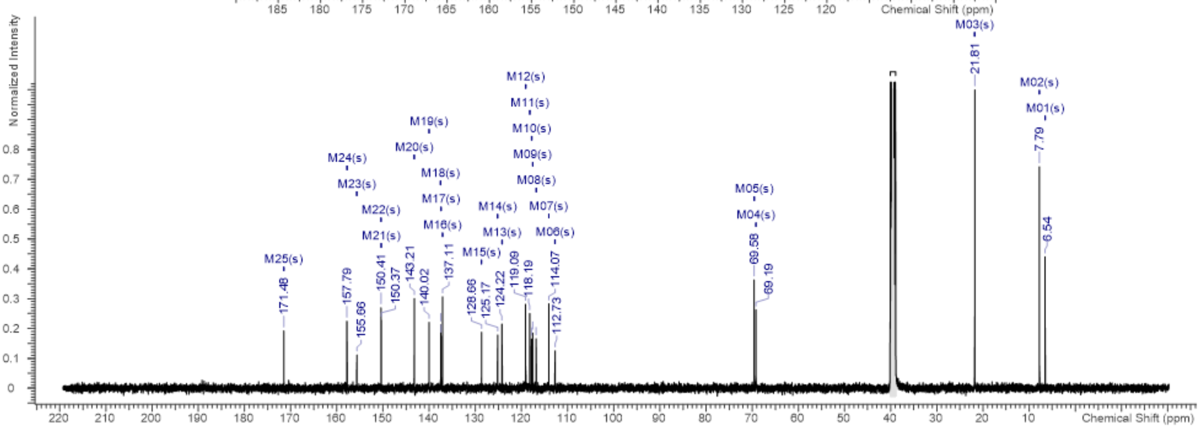
M25(s) 171.48
M24(s) M23(s) M22(s) M21(s) 157.79 155.66 150.41 150.37
M19(s) M20(s) M18(s) M17(s) M16(s) 143.21 140.02 137.11
M15(s) M14(s) M13(s) 128.66 125.17 124.22
M12(s) M11(s) M10(s) M09(s) M08(s) M07(s) M06(s) 119.09 118.19 114.07 112.73
M05(s) M04(s) 69.58 69.19
M03(s) 21.81
M02(s) M01(s) 7.79 6.54
Normalized Intensity
Chemical Shift (ppm)

Compound **33**

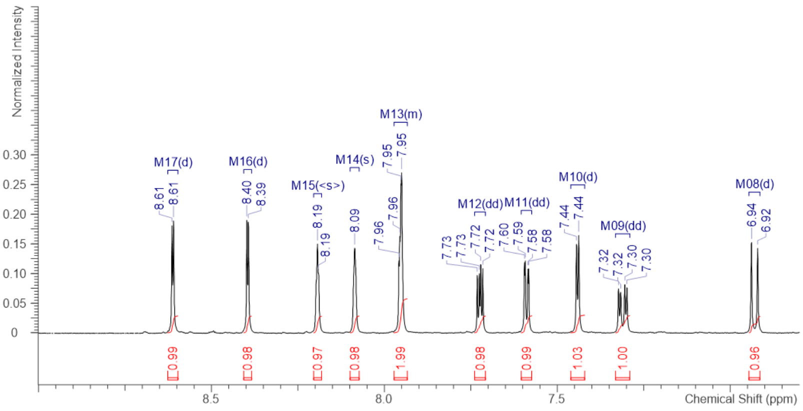
Normalized Intensity
M17(d)
8.61
8.61
M16(d)
8.40
8.39
M15(<s>)
8.19
8.19
M14(s)
8.09
M13(m)
7.96
7.96
7.95
7.95
M12(dd)
7.73
7.73
7.72
7.72
M11(dd)
7.60
7.59
7.58
7.58
M10(d)
7.44
7.44
M09(dd)
7.32
7.32
7.30
7.30
M08(d)
6.94
6.92
0.99
0.98
0.97
0.98
1.99
0.98
0.99
1.03
1.00
0.96
Chemical Shift (ppm)

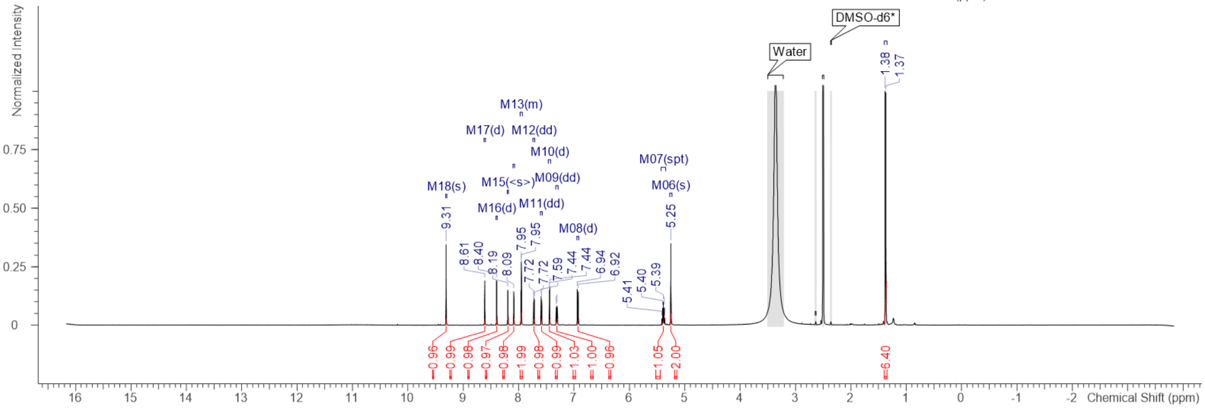
Normalized Intensity
M13(m)
M17(d)
M12(dd)
M10(d)
M18(s)
M15(<s>)
M09(dd)
M16(d)
M11(dd)
M08(d)
M07(spt)
M06(s)
9.31
5.25
5.41
5.40
5.39
Water
DMSO-d6*
1.38
1.37
6.40
2.00
1.05
Chemical Shift (ppm)

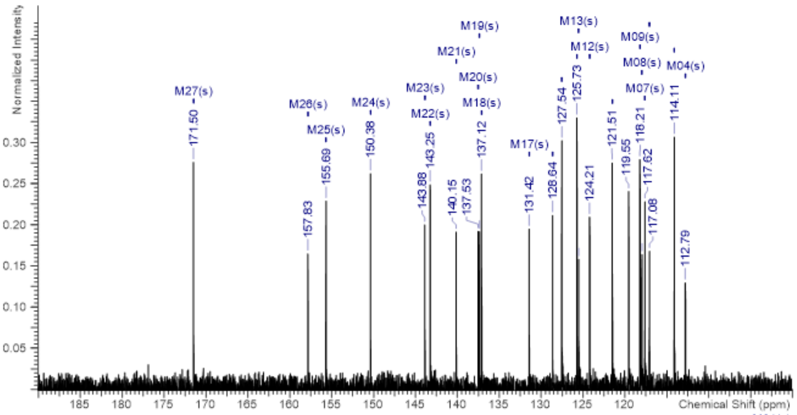
Normalized Intensity
M27(s)
171.50
M26(s)
M25(s)
157.83
155.69
M24(s)
150.38
M23(s)
M22(s)
143.88
143.25
M21(s)
140.15
M19(s)
M20(s)
M18(s)
137.53
137.12
M17(s)
131.42
128.64
M13(s)
M12(s)
127.54
125.73
124.21
121.51
M09(s)
M08(s)
M07(s)
119.55
118.21
117.62
117.08
M04(s)
114.11
112.79
Chemical Shift (ppm)

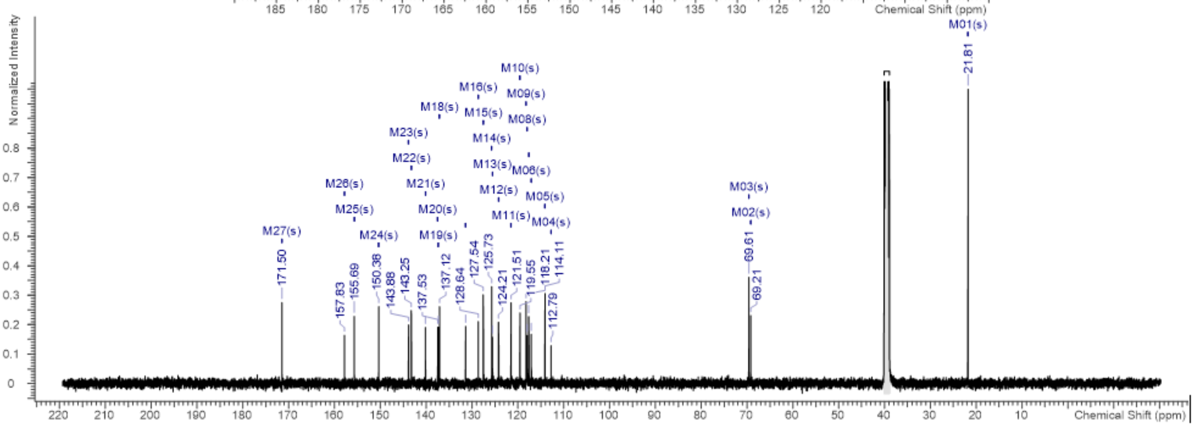
Normalized Intensity
M27(s)
171.50
157.83
155.69
150.38
143.88
143.25
137.53
137.12
128.64
127.54
125.73
124.21
121.51
119.55
118.21
114.11
112.79
M03(s)
M02(s)
69.61
69.21
M01(s)
21.81
Chemical Shift (ppm)

Compound **36**

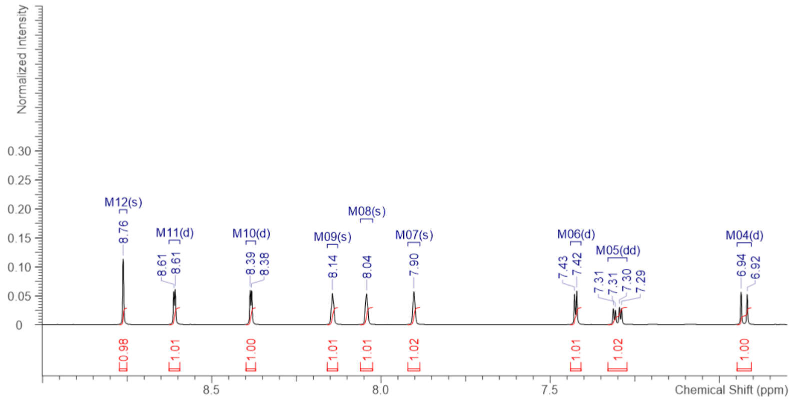
Normalized Intensity
0.30 0.25 0.20 0.15 0.10 0.05 0
M12(s) 8.76
M11(d) 8.61 8.61
M10(d) 8.39 8.38
M09(s) 8.14
M08(s) 8.04
M07(s) 7.90
M06(d) 7.43 7.42
M05(dd) 7.31 7.31 7.30 7.29
M04(d) 6.94 6.92
0.98 1.01 1.00 1.01 1.01 1.02 1.01 1.02 1.00
8.5 8.0 7.5
Chemical Shift (ppm)

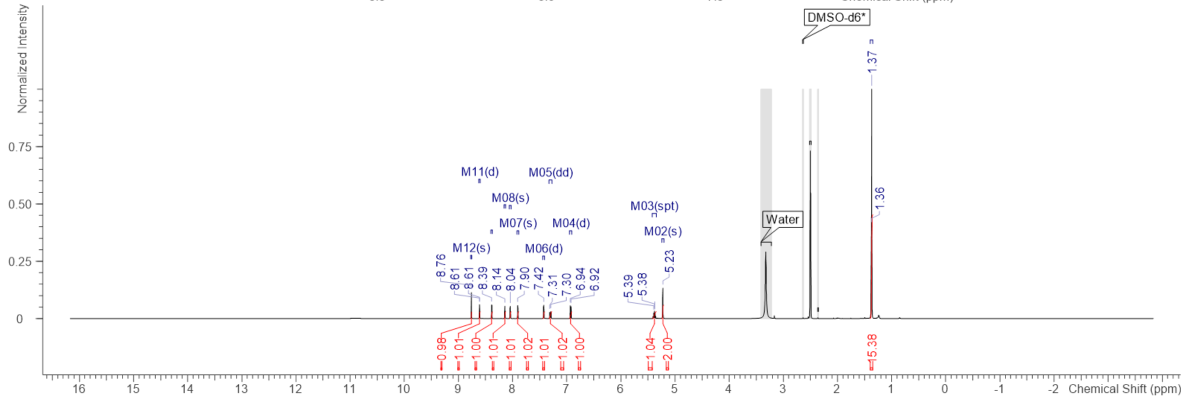
Normalized Intensity
0.75 0.50 0.25 0
DMSO-d6*
Water
M11(d) M05(dd) M08(s) M07(s) M04(d) M12(s) M06(d) M03(spt) M02(s)
8.76 8.61 8.61 8.39 8.14 8.04 7.90 7.42 7.31 7.30 6.94 6.92 5.39 5.38 5.23 1.37 1.36
0.98 1.01 1.00 1.01 1.01 1.02 1.01 1.02 1.00 1.04 2.00 15.38
16 15 14 13 12 11 10 9 8 7 6 5 4 3 2 1 0 -1 -2
Chemical Shift (ppm)

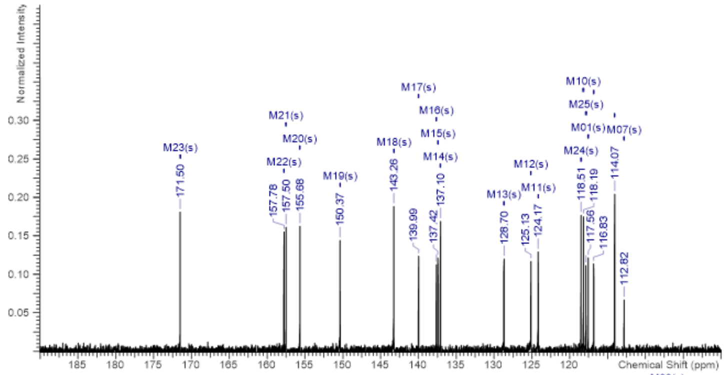
Normalized Intensity
0.30 0.25 0.20 0.15 0.10 0.05
M23(s) 171.50
M21(s) M20(s) M22(s) 157.78 157.50 155.68
M19(s) 150.37
M18(s) 143.26
M17(s) M16(s) M15(s) M14(s) 139.99 137.42 137.10
M13(s) 128.70
M12(s) M11(s) 125.13 124.17
M10(s) M25(s) M01(s) M07(s) M24(s) 118.51 118.19 117.56 116.83 114.07 112.82
185 180 175 170 165 160 155 150 145 140 135 130 125 120
Chemical Shift (ppm)

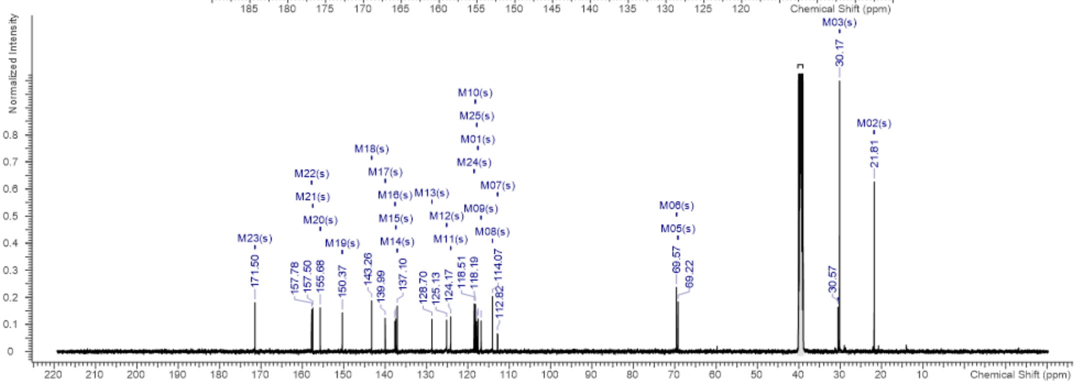
Normalized Intensity
0.8 0.7 0.6 0.5 0.4 0.3 0.2 0.1 0
M23(s) 171.50
M22(s) M21(s) M20(s) 157.78 157.50 155.68
M19(s) 150.37
M18(s) M17(s) M16(s) M15(s) M14(s) 143.26 139.99 137.10
M13(s) M12(s) M11(s) 128.70 125.13 124.17 118.51 118.19
M10(s) M25(s) M01(s) M24(s) M07(s) M09(s) M08(s) 114.07 112.82
M06(s) M05(s) 69.57 69.22
M03(s) 30.57 30.17
M02(s) 21.81
220 210 200 190 180 170 160 150 140 130 120 110 100 90 80 70 60 50 40 30 20 10
Chemical Shift (ppm)

HPLC-MS purity

Compound **18**

Peak Analysis

Injection Details

Injection Name:	**HIPS6458**	*Run Time (min):*	**7.07**
Vial Number:	**RD7**	*Injection Volume:*	**1.00**
Injection Type:	**Unknown**		
Calibration Level:			
Instrument Method:	**0.6ml_+ve_-ve_100-1000-7min**		
Processing Method:	**Processing Method - New (190-380)**	*Dilution Factor:*	**1.0000**
Injection Date/Time:	**04/Mar/24 15:30**	*Sample Weight:*	**1.0000**

UV

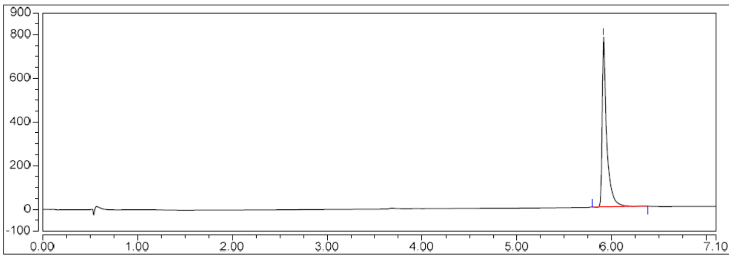

MS

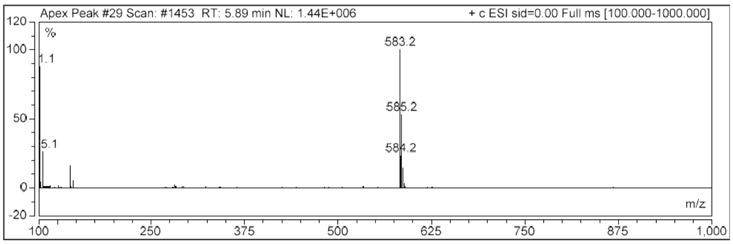

Compound **30**

Peak Analysis

Injection Details

Injection Name:	**HIPS8181**	*Run Time (min):*	**7.07**
Vial Number:	**RD6**	*Injection Volume:*	**4.00**
Injection Type:	**Unknown**		
Calibration Level:			
Instrument Method:	**0.6ml_+ve_-ve_100-600-7min**		
Processing Method:	**Processing Method**	*Dilution Factor:*	**1.0000**
Injection Date/Time:	**16/Nov/23 14:02**	*Sample Weight:*	**1.0000**

UV

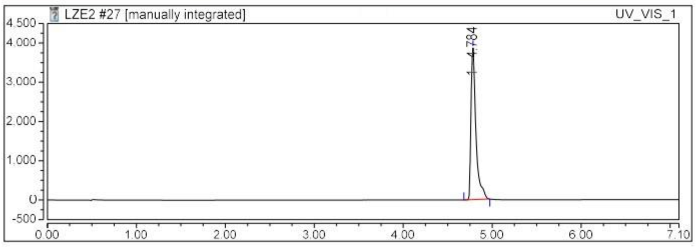

MS

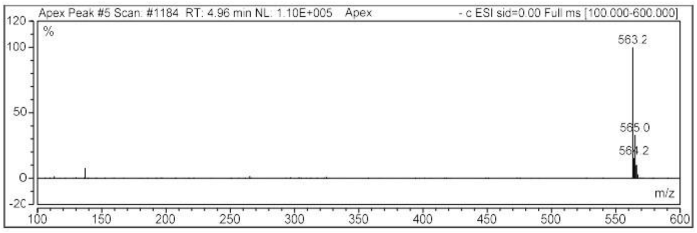

Compound **31**

Peak Analysis

Injection Details

Injection Name:	**HIPS8189**	*Run Time (min):*	**7.07**
Vial Number:	**RD5**	*Injection Volume:*	**2.00**
Injection Type:	**Unknown**		
Calibration Level:			
Instrument Method:	**0.6ml_+ve_-ve_100-600-7min**		
Processing Method:	**Processing Method**	*Dilution Factor:*	**1.0000**
Injection Date/Time:	**17/Nov/23 13:50**	*Sample Weight:*	**1.0000**

UV

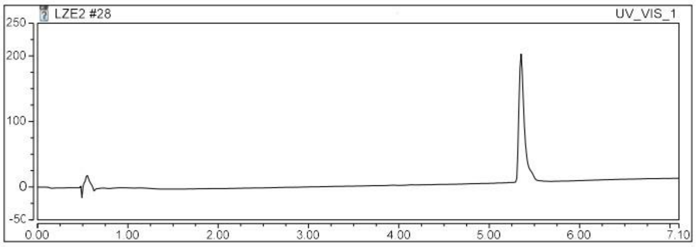

MS

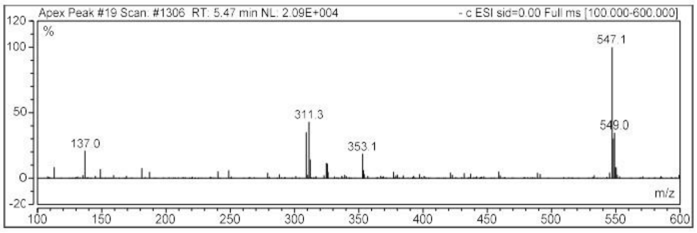

Compound **32**

Peak Analysis

Injection Details

Injection Name:	**HIPS8250**	*Run Time (min):*	**5.07**
Vial Number:	**RD6**	*Injection Volume:*	**2.00**
Injection Type:	**Unknown**		
Calibration Level:			
Instrument Method:	**0.6ml_+ve_-ve_100-1000**		
Processing Method:	**Processing Method**	*Dilution Factor:*	**1.0000**
Injection Date/Time:	**06/Mar/24 17:07**	*Sample Weight:*	**2.0000**

UV

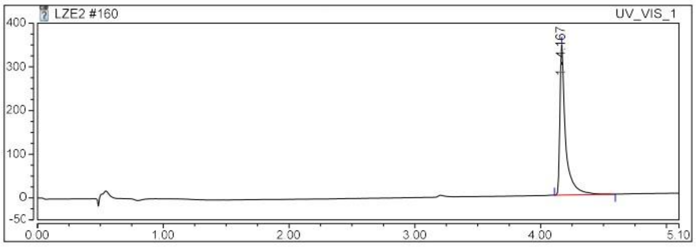

MS

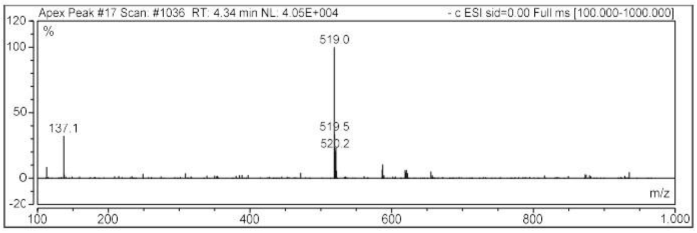

Compound **33**

Peak Analysis

Injection Details

Injection Name:	**HIPS8251**	*Run Time (min):*	**5.07**
Vial Number:	**RD6**	*Injection Volume:*	**3.00**
Injection Type:	**Unknown**		
Calibration Level:			
Instrument Method:	**0.6ml_+ve_-ve_100-1000**		
Processing Method:	**Processing Method**	*Dilution Factor:*	**1.0000**
Injection Date/Time:	**11/Jan/24 12:44**	*Sample Weight:*	**2.0000**

UV

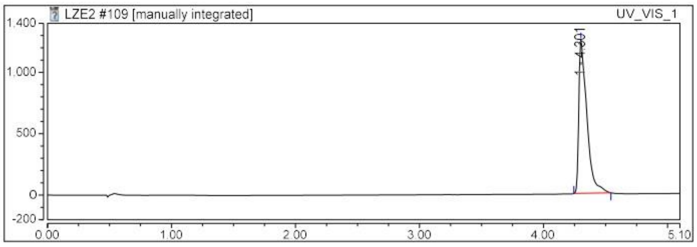

MS

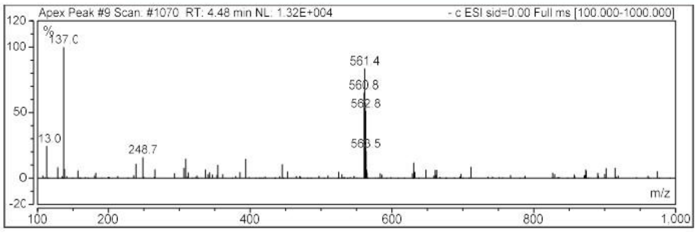

Compound **36**

Peak Analysis

Injection Details

Injection Name:	**HIPS8219**	*Run Time (min):*	**5.07**
Vial Number:	**RD6**	*Injection Volume:*	**4.00**
Injection Type:	**Unknown**		
Calibration Level:			
Instrument Method:	**0.6ml_+ve_-ve_100-1000**		
Processing Method:	**Processing Method**	*Dilution Factor:*	**1.0000**
Injection Date/Time:	**12/Dec/23 11:43**	*Sample Weight:*	**2.0000**

UV

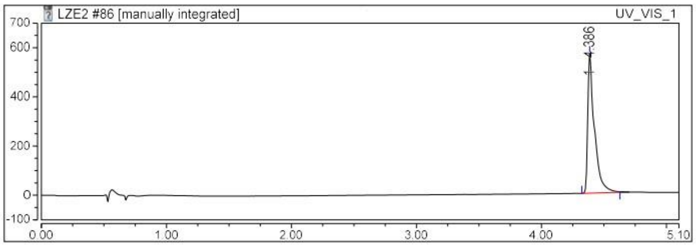

MS

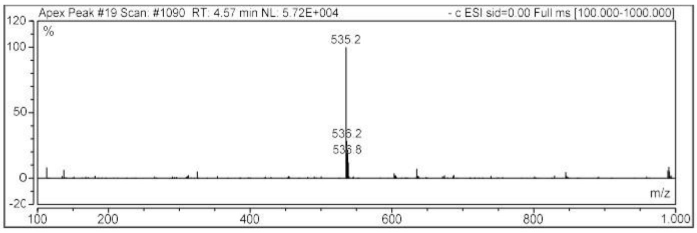

Supplement: Supplementary file 1 [file jm5c02721_si_001.pdf]
